# Tumor-associated macrophages/C-X-C motif chemokine ligand 1 promotes breast cancer autophagy-mediated chemoresistance via IGF1R/STAT3/HMGB1 signaling

**DOI:** 10.1038/s41419-024-07123-5

**Published:** 2024-10-11

**Authors:** Bowen Yang, Guanzhi Li, Shengqi Wang, Yifeng Zheng, Juping Zhang, Bo Pan, Neng Wang, Zhiyu Wang

**Affiliations:** 1 State Key Laboratory of Dampness Syndrome of Chinese Medicine, Chinese Medicine Guangdong Laboratory, The Second Affiliated Hospital of Guangzhou University of Chinese Medicine, Guangzhou, China; 2 Breast Disease Specialist Hospital of Guangdong Provincial Hospital of Chinese Medicine, The Second Clinical College of Guangzhou University of Chinese Medicine, Guangzhou, Guangdong China; 3 Research Centre of Basic Integrative Medicine, School of Basic Medical Sciences, Guangzhou University of Chinese Medicine, Guangzhou, Guangdong China; 4 Guangdong Provincial Key Laboratory of Clinical Research on Traditional Chinese Medicine Syndrome, Guangdong Provincial Academy of Chinese Medical Sciences, Guangdong Provincial Hospital of Chinese Medicine, Guangzhou, China; 5 Guangdong-Hong Kong-Macau Joint Lab on Chinese Medicine and Immune Disease Research, Guangzhou University of Chinese Medicine, Guangzhou, China

**Keywords:** Breast cancer, Autophagy

## Abstract

Autophagy-mediated chemoresistance is the core mechanism for therapeutic failure and poor prognosis in breast cancer. Breast cancer chemotherapy resistance is believed to be influenced by tumor-associated macrophages (TAMs), by which C-X-C motif chemokine ligand 1 (CXCL1) is the most abundant cytokine secreted. Yet, its role in mediating autophagy-related chemoresistance is still unknown. This study aimed to explore the molecular mechanisms by which TAMs/CXCL1 induced autophagy-mediated chemoresistance in breast cancer. It was found that TAMs/CXCL1 promoted chemoresistance of breast cancer cells through autophagy activation in vitro, and *CXCL1* silence could enhance the chemosensitivity of paclitaxel-resistant breast cancer cells via autophagy inhibition. A high-throughput quantitative PCR chip and subsequent target validation showed that CXCL1 induced autophagy-mediated chemoresistance by inhibiting VHL-mediated IGF1R ubiquitination. The elevated IGF1R then promoted STAT3/HMGB1 signaling to facilitate autophagy. Additionally, TAMs/*CXCL1* silence improved paclitaxel chemosensitivity by suppressing autophagy in breast cancer mice xenografts, and clinical studies further linked CXCL1 to IGF1R/HMGB1 signaling, as well as shorter free survival of recurrence. Taken together, these results not only uncover the crucial role of TAMs/CXCL1 signaling in mediating breast cancer chemoresistance through enhancing autophagy, but also shed novel light on the molecular mechanism of IGF1R/STAT3/HMGB1 pathway in regulating autophagy and its impact on cancer prognosis.

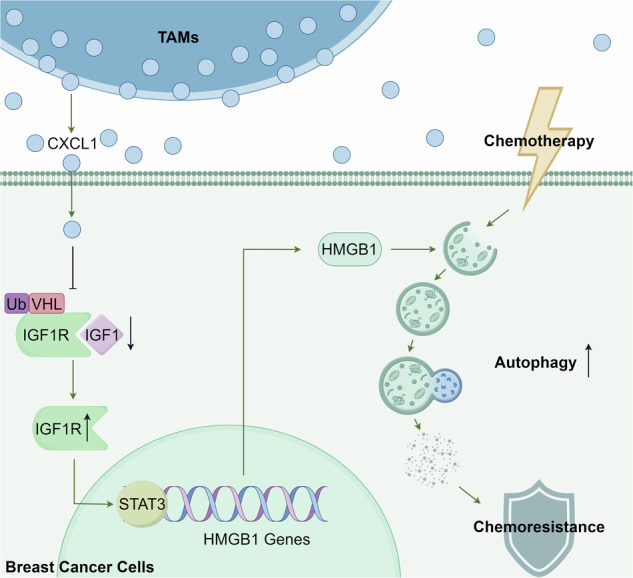

## Introduction

Breast cancer is the most common malignancy and ranks as the primary cause of cancer-related mortality in women globally [1]. It has been estimated that approximately 12% of new cancer cases and 7% of new cancer-related deaths are attributed to breast cancer among women worldwide. Despite the introduction of neoadjuvant chemotherapy, immunotherapy, and targeted therapy brings new hope to prolong overall survival, more than 680,000 individuals still die of breast cancer each year [1, 2]. The greatest threat to breast cancer patients is resistance to refractory tumors, leading to treatment failure and subsequent cancer death [3]. In particular, primary or acquired chemoresistance is responsible for 90% of all cancer-related deaths, with multiple factors identified as contributing to chemoresistance, including enhanced drug efflux, significant gene mutation, aberrant drug metabolism, and others [4, 5]. Adaptive stress serves as a core mechanism in the mediation of chemoresistance by protecting cancer cells from the cytotoxic effects of chemotherapy. Therefore, molecular elucidation of adaptive stress regulation is significant for developing therapeutic strategies against chemoresistance.

Autophagy, an essential catabolic process that regulates adaptive stress responses, plays a significant role in maintaining cellular homeostasis, modulates cellular signaling, and promotes cellular survival [6, 7]. Notably, autophagy displays a dual function in cancer chemotherapy. On one hand, chemotherapeutic drugs can induce autophagic death of cancer cells. On the other hand, autophagic activation is always concomitant with cancer chemoresistance by providing cancer cells with nutrients and energy to escape from an unfavorable tumor microenvironment (TME) [8]. ATP-binding cassette efflux transporter G2 (ABCG2), highly expressed in various tumor tissues, is considered as the most important mediator of cancer multidrug resistance [9]. Recent reports have shown a strong association between ABCG2 expression and autophagy activation. It was revealed that the upregulation of autophagy-related gene HMGB1 substantially elevated ABCG2 levels, thereby promoting chemoresistance in colorectal cancer. Besides, the autophagy inhibitor 3-Methyladenine (3-Ma) was found to suppress ABCG2 expression and enhance chemosensitivity [10]. Yet, further studies are needed to determine how autophagy regulates ABCG2 expression. In addition, accumulating evidence showed that the autophagic level of cancer cells might be induced by cytokines, hypoxia, oxidative stress, and other factors in TME [11]. For example, Wang *et al*. showed that TGF-β1 in TME could promote autophagy and inhibit apoptosis of breast cancer cells by inhibiting TP63 expression [12]. Several studies also highlighted TME in multi-drug resistance [13]. In breast cancer, collagens and fibronectin secreted by cancer-associated fibroblasts in TME had been shown to induce multi-drug resistance by activating PI3K/AKT and Ras/Raf/MEK/ERK1-2 pathways [14]. Factors such as CXCL1, CXCL2, and IL-8 released by cancer-associated mesenchymal stromal cells were also involved in the chemoresistance of ovarian cancer cells [15]. However, it remains to be clarified concerning the key factors in TME responsible for inducing chemoresistance through autophagy regulation.

As the most abundant immune cells within TME, tumor-associated macrophages (TAMs) play a crucial role in promoting tumor growth, metastasis, and chemoresistance through the secretions of growth factors, cytokines, or chemokines. A clinical study revealed a close correlation between high TAMs infiltration and shorter progression-free survival (PFS) as well as overall survival (OS) in non-small cell lung cancer, suggesting that TAMs enrichment could induce resistance to immune checkpoint blockers [16]. Meanwhile, TAMs, along with the cancer-associated fibroblasts and extracellular matrix, could protect cancer cells from chemotherapy by creating an outlayer barrier [17]. It was also found that TAMs could promote cancer cell chemoresistance by activating STAT3 signaling through secretion of IL-6 and TNF-α [18]. C-X-C motif chemokine ligand 1 (CXCL1) is the chemokine that TAMs secrete most abundantly [19], and TAMs/CXCL1 signaling has been demonstrated to aggravate breast cancer progression by enhancing the epithelial-mesenchymal transition and pre-metastatic niche formation [20]. In addition, CXCL1/HMGB1 autophagic axis might be triggered following chemotherapy in breast cancer [21]. Nevertheless, the precise mechanism by which TAMs/CXCL1 regulate chemoresistance remains unclear in breast cancer.

In the present study, it was demonstrated that TAMs/CXCL1 enhanced chemoresistance in parental or chemoresistant breast cancer cells through autophagic activation. IGF1R/STAT3/HMGB1 axis was identified as the downstream signaling involved in CXCL1-mediated autophagy. *CXCL1* knockdown in TAMs demonstrated a remarkable chemosensitizing effect on breast cancer xenografts, along with blockage of IGF1R/STAT3/HMGB1 signaling in vivo. Clinically, CXCL1 was shown to be an independent predictor of poor prognosis and to be positively correlated with IGF1R/STAT3/HMGB1 expression in breast cancer. Taken together, the results not only highlight the crucial role of TAMs/CXCL1 in regulating autophagy-induced chemoresistance but also shed novel light on the molecular mechanism of IGF1R/STAT3/HMGB1 pathway in autophagy modulation.

## Materials and methods

### Cell culture

The cell lines MDA-MB-231, MCF-7, and THP1 were obtained from Nanjing KeyGen Biotech (Nanjing, China). Authentication of these cells has been validated based on the results of short tandem repeat profiling. MDA-MB-231 and MCF-7 cell lines were cultured in Dulbecco’s Modified Eagle Medium (DMEM) supplemented with 10% fetal bovine serum (FBS) and 1% penicillin-streptomycin (PS). THP-1 cells were cultured in Roswell Park Memorial Institute (RPMI) 1640 medium supplemented with 10% FBS and 1% PS. All the cells were maintained at 37 °C in a humidified atmosphere with 5% CO_2_. For the pololization of TAMs, 1 μg/mL Phorbol-12-myristate-13-acetate was used to induce M0-TAMs in THP-1 cells for 24 h, while 10 ng/mL IL-4 was used to induce M2-TAMs in M0-TAMs for 72 h.

### Cell transfections

The *CXCL1*-shRNA lentivirus (The shRNA target sequence was 5′-GGTATGATTAACTCTACCTGC-3′) and *CXCL1*-overexpressing lentivirus were acquired from Genepharma (Shanghai, China), and the procedures of transfection and screening were carried out in accordance with manufacturer’s guidelines. si*ATG5* (Target sequence was 5′-AGCUUCUGAAUGAAAGGUCTT-3′, Genepharma, Shanghai, China), si*VHL* (Target sequence was 5′-GGAGCGCAUUGCACAUCAATT-3′, Vigene, Jinan, China), si*IGF1R* (Target sequence was 5′-AUUGUGCCGGUAAAGGUAG-3′, Vigene, Jinan, China), and their negative control were transfected into the cells by Lipo6000 transfection reagent (C0526, Beyotime, Shanghai, China), according to the manufacturer’s instructions.

### Cell counting assay and colony formation assay

The effect of TAMs/CXCL1 on the growth of breast cancer cells was detected using cell counting assay and colony formation assay as previously reported [21]. For cell counting assay, cells were seeded at a density of 2.5 × 10^5^ cells per well in 6-well plates. Following the indicated treatments, cells were counted using trypan blue and a Cellometer Mini device (Nexcelom, Boston, MA, USA). For the colony formation assay, cells were seeded at a density of 1 × 10^3^ cells per well in 6-well plates. After the indicated treatment for 4 h, the supernatant medium was replaced with fresh medium, and the cells were cultured for an additional two weeks. At the end of the experiment, the colonies were fixed with 4% paraformaldehyde, stained with Coomassie Blue solution, and photographed under a microscope for quantification. For CCK8 assay, cells were seeded at a density of 1 × 10^3^ cells per well in 96-well plates. After indicated treatments for 48 h, cell viability was assessed using the CCK8 Kit (C0038, Beyotime Biotechnology, Shanghai, China) following the manufacturer’s instructions.

### Cell immunofluorescence

Breast cancer cells were plated onto Laser confocal petri dishes at a concentration of 1 × 10^5^ cells per well for overnight attachment. Subsequently, the cells were exposed to mRFP-GFP-LC3 lentiviral vectors (HB-LP2100001, HanBio Technology, Shanghai, China) following the manufacturer’s instructions. Following the indicated treatments for 24 h, fluorescence images were captured using an LMS710 confocal microscope (Zeiss, Oberkochen, Germany) and quantitatively analyzed using Image J software.

### Flow cytometry analysis

APC-F4/80 (17-4801-82, eBioscience) antibody and PE-CD163 (12-1631-82, eBioscience) antibody were purchased from Thermo Fisher Scientific. A flow cytometry analysis of M2-TAMs was described in our previous study based on the NovoCyte Quanteon flow cytometer (ACEA Biosciences, San Diego, CA, USA) [22]. Analysis of the results was performed using NovoCyte analysis software (ACEA Biosciences, San Diego, CA, USA).

### Western blotting detection

Western blotting assay was performed as previously described [23]. Cells were treated with the indicated treatment for 24 h. This study involved primary antibodies including β-actin (60008-1-Ig, Proteintech), STAT3 (10253-2-AP, Proteintech), *p*-STAT3 (39596, Proteintech), LC3 (14600-1-AP, Proteintech), SQSTM1/p62 (18420-1-AP, Proteintech), HMGB1 (10829-1-AP, Proteintech), Ubiquitin (10201-2-AP, Proteintech), VHL (24756-1-AP, Proteintech), CXCL1 (AF5403, Affinity), IGF1 (DF6096, Affinity), ABCG2 (AF5177, Affinity), IGF1R (AF6125, Affinity), IGF1R (3027, Cell Signaling Technology), as well as secondary antibodies goat anti-rabbit IgG (SA00001-2, Proteintech) and goat anti-mouse IgG (SA00001-1, Proteintech). Analysis of protein expression levels was carried out using Image J software.

### Dual-Luciferase reporter gene assay

Dual-Luciferase reporter gene assay was performed as previously described [24]. HMGB1 dual-Luciferase reporter gene vector (HPRM43498-PG04, GeneCopoeia, Shanghai, China) and luciferase assay kit (LF031, GeneCopoeia, Shanghai, China) were purchased from GeneCopoeia.

### High-throughput quantitative PCR technique

Total RNA was isolated from from MDA-MB-231 cells (Sample A1, A2, A3), MDA-MB-231 cells treated with the conditional medium of M2-TAMs (TAMs-CM) (Sample B1, B2, B3), MDA-MB-231R cells (Sample C1, C2, C3), and MDA-MB-231 *CXCL1*^*OE*^ cells (Sample D1, D2, D3) using Trizol (15596026, Life Technologies). Subsequently, autophagy-related PCR arrays were detected by the Applied Biosystems ViiA^TM^ 7 Real-Time PCR System (Wcgene Biotechnology, Shanghai, China) as previously reported [21].

### RT-*q*PCR assay

Cells were seeded on 6-well plates with the indicated interventions. Next, the total RNA was extracted following the instructions of TIANGEN RNAsimple Total RNA Kit (DP419, TIANGEN). RT-*q*PCR detection was conducted with a cDNA reverse transcription kit (6210 A, TaKaRa) and real-time PCR kit (RR047A, TaKaRa) using a CFX96 Real-Time System (Bio-Rad, Hercules, CA, USA). PCR primer sequences were listed below: For HMGB1 primers: forward TAGCCACTAACCTTGCCTGG; reverse GCTGTGCACCAACAAGAACC; For β-actin primers: forward CCAGAGGCGTACAGGGATAG; reverse CCAACCGCGAGAAGATGA.

### Electron microscopy detection

For electron microscopy assay, cells were seeded into a 100 mm dish with indicated interventions, then trypsinized and fixed in glutaraldehyde fixative solution for 1 h at room temperature. Following 1% osmium tetroxide/1.5% potassium ferrocyanide treatment, 1% uranyl acetate was added, and the samples were dehydrated and embedded in epon-araldite. PhilipCM20 transmission electron microscopes were used to observe autophagosomes.

### Coimmunoprecipitation (CoIP) assay

CoIP assay was performed as previously described [25]. A total of 2000 μg of cellular protein was extracted from 1 × 10^7^ cells. A fraction of protein extracts was denatured with 5 × Laemmli SDS-sample buffer for 5 min and used as the input sample, while the remaining extracts were incubated overnight at 4 °C with the IGF1R antibody (3027, Cell Signaling Technology). Subsequently, the antibody-preincubated sample was transferred to a CoIP assay kit (635721, TaKaRa) and processed according to the manufacturer’s instructions.

### Chromatin immunoprecipitation (ChIP) assay

ChIP assay was performed as previously described [26]. The JASPAR database and hTFtarget predicted STAT3 binding sites (S1: −515 to −525 and S2: −1529 to −1521) at the promoter region of HMGB1. A ChIP assay kit (P2080S, Beyotime, Shanghai, China) and STAT3 antibody (10253-2-AP, Proteintech) were used for the ChIP assay. By using primers as follows, we amplified this region in the immune-precipitated DNA samples: (1) S1 forward 5′-TGGGCTCCCCAGTTCTTCTT-3′ and reverse 5′-TGACTTACTGTGCGAAGAGGG-3′. (2) S2 forward 5′-CACGCTCCTTGGAAAACGAAA-3′ and reverse 5′-ATCGACCCGGTACCAAGAAG-3′.

### Establishment of a human breast cancer xenotransplantation model in mice

The animal study was approved by the Institutional Animal Care and Use Committee at Guangzhou University of Chinese Medicine (Ethical number 2020034; 20231214002). 4-week-old female Balb/c-nu-nu mice were purchased from Guangdong Medical Laboratory Animal Center and kept in the Research Center of Basic Integrative Medicine, Guangzhou University of Chinese Medicine. Mice were randomly allocated to six groups. In particular, TAMs or TAMs *CXCL1*^*KD*^ cells (9 × 10^5^) were mixed with MDA-MB-231 cancer cells (3 × 10^5^), and injected into the mammary fat pads of mice. Then, 100 μL saline without or with paclitaxel (10 mg/kg/ 3 days) and 3-Ma (10 mg/kg/day) were given by intraperitoneal injection. Tumor volumes (V) were measured once per 3 days and calculated with the formula V = 0.5 × (length) × (width) × (width). At the end of the experiment on day 21, tumors from each group were collected, weighed, and compared. The investigator remained blinded to the group allocation of the mice throughout the experiment. The calculation of sample size was based on our previous studies [27, 28]. The sample size was specified in the respective figure legend.

### Immunohistochemistry (IHC) assay

Tissues were fixed in 4% paraformaldehyde for 24 h, embedded in paraffin, and chopped into 4 μm thickness. The following steps were performed according to an immunohistochemistry kit (PV-6000, Zhongshan Jinqiao, Beijing, China). Primary antibodies included in this study were Ki67 (AF1738, Beyotime), CXCL1 (AF5403, Affinity), IGF1R (AF6125, Affinity), ABCG2 (AF5177, Affinity), HMGB1 (10829-1-AP, Proteintech), CD163 (16646-1-AP, Proteintech), and LC3 (14600-1-AP, Proteintech).

### Tumor tissue microarrays (TMA) analysis

The tissue microarrays (HBreD140Su03) were brought from Shanghai Outdo Biotech Company (Shanghai, China), and were utilized for IHC staining. Images were scored according to the intensity of the staining for semi-quantitative analysis. Yellowish-brown or tan granules observed in the tissues were deemed positive. The scores of staining positive rates were fixed as follows: no points for nonpositive cells, one point for 1% ~ 25%, two points for 26% ~ 50%, three points for 51% ~ 75%, and four points for 76% ~ 100%. Based on the staining intensity scale, negative staining was scored as 0, weak staining 1, intermediate staining 2, and strong staining 3. Lastly, staining scores were determined by positive rates scores × intensity scores: low-expression group, < 8; high-expression group, ≥ 8.

### ELISA detection

M2-TAMs and MDA-MB-231 cells were seeded at a density of 5 × 10^5^ cells per well in 6-well plates, respectively. The conditioned medium of both cell types were then collected, and the CXCL1 secretion levels were measured using a CXCL1 ELISA kit (Cloud-Clone, Wuhan, China, SEA041Hu) according to the instructions.

### Statistical analysis

SPSS 17.0 software (Abbott Laboratories, Chicago, IL, USA) and GraphPad Prism 8.0 (GraphPad Software, San Diego, CA, USA) were used for statistical analyses. Data were represented as the mean ± SD from three independent experiments performed in triplicate at least. A two-sided Student *t*-test was used to evaluate comparisons between the two groups. Three or more groups were compared using one-way ANOVA with Tukey’s or Dunnett’s post-hoc analysis. Repeated measures analysis of variance (ANOVA) was performed to determine differences between tumor growth curves. GraphPad Prism 8.0 was used to generate Kaplan-Meier curves, Pearson correlations, and *P* values. Cox regression analysis was performed using SPSS17.0 software. All tests statistical significance was determined by a *P* < 0.05.

## Results

### TAMs enhanced the chemoresistance of breast cancer cells through activation of autophagy

**Fig. 1:**
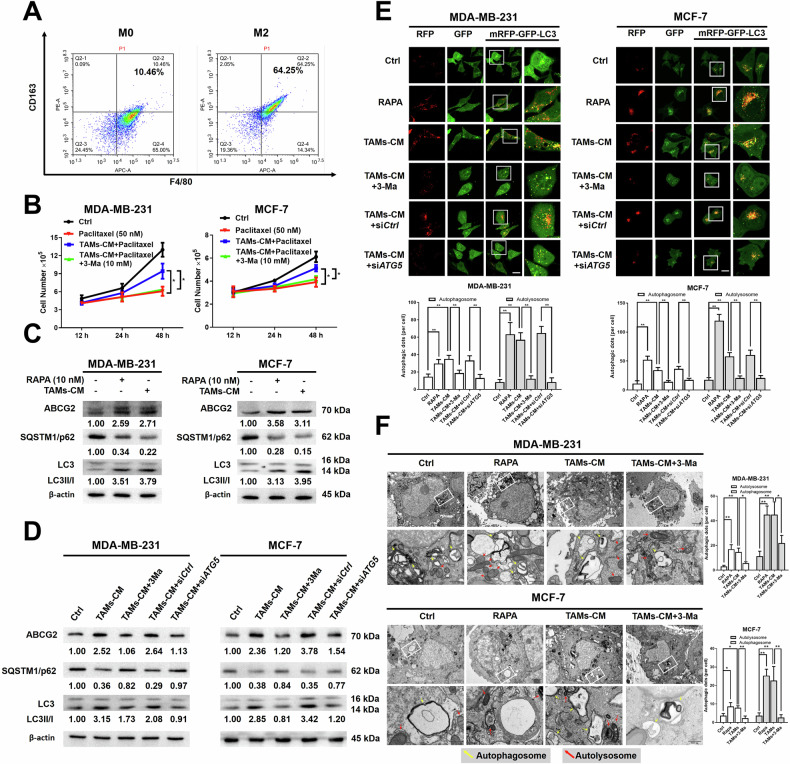
TAMs enhanced breast cancer cell chemoresistance by activating autophagy. **A** Flow cytometry analysis confirmed the successful induction of M2-TAMs by staining with CD163 and F4/80. **B** Cell counting assay was performed in MDA-MB-231 and MCF-7 cells to determine the effect of TAMs-CM on the cell growth response to paclitaxel (50 nM) with or without autophagic inhibitor 3-Ma (10 mM) after 48 h treatment. **C** Western blotting assay was conducted to evaluate the effects of TAMs-CM on expressions of ABCG2, SQSTM1/p62 and LC3 levels in MDA-MB-231 and MCF-7 cells. The autophagic activator rapamycin (RAPA, 10 nM) was used as a positive control. **D** Western blotting assay was conducted to evaluate the synergistic effects of TAMs-CM with autophagic inhibition by either 3-Ma administration or *ATG5* knockdown on expressions of ABCG2, SQSTM1/p62 and LC3 levels. **E** The autophagic flux of MDA-MB-231 cells and MCF-7 cells was assessed through transfection with mRFP-GFP-LC3 lentiviral vectors after exposure to TAMs-CM, with or without autophagic inhibition by either 3-Ma administration or *ATG5* knockdown. The autophagic activator RAPA (10 nM) was used as a positive control (*n* = 5, scale bars indicate 20 μm). **F** Transmission electron microscope detection demonstrated the presence of autolysosomes (red arrows) and autophagosomes (yellow arrows) in MDA-MB-231 and MCF-7 cells after the indicated treatments. Values are presented as Mean ± SD, *n* = 3 unless otherwise indicated, *P < 0.05, **P < 0.01.

In this study, the human monocytic cell line THP1 was differentiated into M2 macrophages through treatment with IL-4 and verified by flow cytometry analysis (Fig. 1A). Subsequently, the influence of TAMs on autophagy-related chemoresistance was assessed using breast cancer cells MDA-MB-231 and MCF-7. The results in Fig. 1B demonstrated that TAMs-CM significantly reduced the cytotoxicity of paclitaxel on both cell lines after 48 h treatment, while partial reversal of this effect was observed following 3-Ma treatment. Western blotting analysis then revealed that either TAMs-CM or rapamycin (RAPA) increased autophagic flux, presented as an increase in LC3-I to LC3-II conversions as well as a decrease in SQSTM1/p62 expressions in both MDA-MB-231 and MCF-7 cells (Fig. 1C). Despite TAMs-CM was capable of enhancing LC3-II/I conversion and decreasing SQSTM1/p62 expressions, the effects were in part offset by either 3-Ma administration or autophagy-related gene 5 (*ATG5*) knockdown (Fig. 1D). Such phenomenon was further confirmed using the mRFP-GFP-LC3 reporter system. In particular, autolysosomes are labeled by free red fluorescence (mRFP), while autophagosomes represent yellow fluorescence (mRFP and GFP colocalized). It was found that either TAMs-CM or RAPA administration caused an increase in yellow and red puncta in breast cancer cells, while 3-Ma treatment or *ATG5* knockdown reversed the autophagy-promoting effects of TAMs-CM (Fig. 1E). Additionally, the electron microscope showed that TAMs-CM and RAPA increased the number of autophagosomes (yellow arrows) and autolysosomes (red arrows) in both breast cancer cells, while 3-Ma partly abrogated the effects (Fig. 1F). Taken together, these findings demonstrated that M2-TAMs could activate autophagy and contribute to chemoresistance of breast cancer cells.

### TAMs/CXCL1 signaling promoted autophagy-mediated chemoresistance in breast cancer cells

**Fig. 2:**
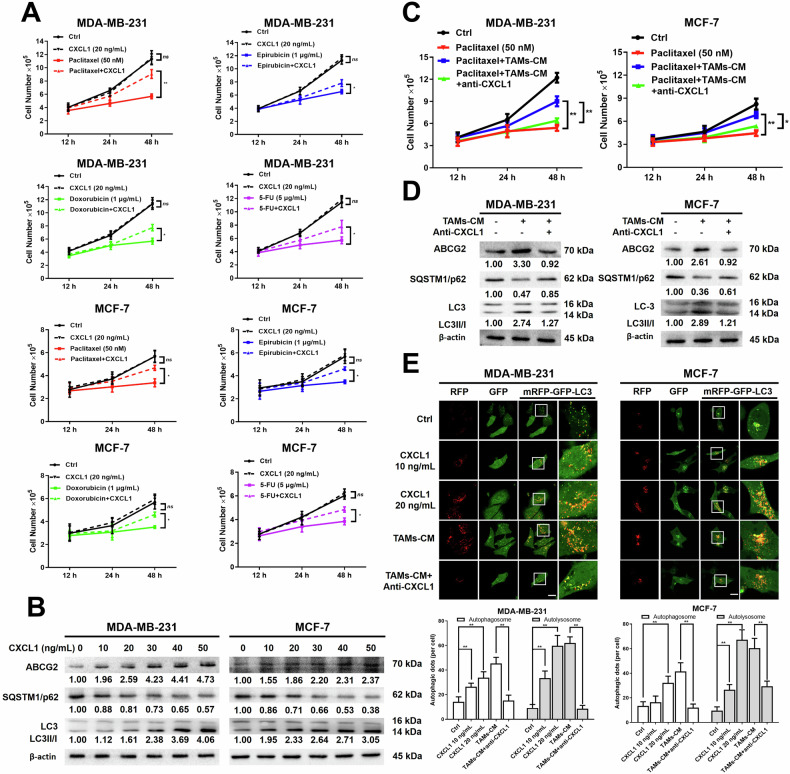
TAMs/CXCL1 signaling promoted autophagy-mediated chemoresistance in breast cancer cells. **A** Cell counting assay was conducted to examine CXCL1 (20 ng/mL) and its combination with several chemotherapeutic drugs (Paclitaxel at 50 nM, Epirubicin at 1 μg/mL, Doxorubicin at 1 μg/mL, and 5-FU 5 μg/mL) on cell growth of MDA-MB-231 and MCF-7 cells after 48 h treatment. **B** Western blotting assay was applied to detect the expressions of ABCG2, SQSTM1/p62 and LC3 levels in the presence of CXCL1 ranging from 0 to 50 ng/mL after 24 h treatment. **C** Cell counting assay was used to detect the effect of CXCL1 neutralizing antibodies (20 ng/mL) on the chemosensitivity of MDA-MB-231 and MCF-7 in response to TAMs-CM. **D** Western blotting assay was performed on MDA-MB-231 and MCF-7 cells to determine the effects of TAMs-CM containing a CXCL1 neutralizing antibody (20 ng/mL) on ABCG2, SQSTM1/p62 and LC3 expression levels. **E** The mRFP-GFP-LC3 reporter assay was carried out to verify autophagic alternations in the presence of either CXCL1 (10–20 ng/mL) or TAMs-CM containing CXCL1 neutralizing antibodies (20 ng/mL) (*n* = 5, scale bars indicate 20 μm). Values are presented as Mean ± SD, *n* = 3 unless otherwise indicated, *P < 0.05, **P < 0.01.

The investigation focused on the impact of CXCL1 on chemoresistance in breast cancer, given its high abundance as a chemokine secreted by TAMs in metastatic breast cancer [19]. This suggested that CXCL1 primarily originated from TAMs. Subsequently, cell growth was examined on breast cancer cells in the presence of CXCL1 cytokine or its neutralizing antibody to determine their response to several chemotherapy drugs. As shown in Fig. 2A, the effects of CXCL1 on MDA-MB-231 and MCF-7 cells were studied either alone or in combination with paclitaxel, epirubicin, doxorubicin and 5-Fluorouracil (5-FU). According to the results, CXCL1 significantly reduced the cytotoxicity of chemotherapy drugs in MDA-MB-231 and MCF-7 cells. Compared with the other three chemodrugs, CXCL1 had the most potent chemoresistance-inducing effects on paclitaxel. Moreover, western blotting analysis revealed an increase in ABCG2 and LC3-II accumulation, as well as a decrease in SQSTM1/p62 expression in breast cancer cells treated with CXCL1 for 24 h (Fig. 2B). These findings suggested that CXCL1 was associated with decreased chemosensitivity and enhanced autophagy in breast cancer. Furthermore, a neutralizing antibody against CXCL1 was introduced into the TAM culture system to assess breast cancer chemosensitivity. It was found that TAMs-CM effectively counteracted the gowth inhibition caused by paclitaxel, while the CXCL1 neutralizing antibody reversed this stimulatory effect of TAMs-CM in MDA-MB-231 and MCF-7 cells (Fig. 2C). The CXCL1 neutralizing antibody also reversed the influence of TAMs-CM on the expressions of ABCG2, SQSTM1/p62, and LC3-II/I in response to paclitaxel treatment (Fig. 2D). Additionally, the mRFP-GFP-LC3 reporter assay was carried out to validate the autophagy-inducing property of CXCL1. Both breast cancer cells treated with CXCL1 exhibited an increase in yellow and free red puncta with increasing doses, while the CXCL1 neutralizing antibody prevented the onset of autophagy induced by TAMs-CM (Fig. 2E). To elucidate the primary source of CXCL1 in breast cancer, an ELISA assay was additionally performed to measure CXCL1 concentrations in the supernatants of TAMs, MDA-MB-231 cells, TAMs transfected with an empty vector (TAMs Vec), TAMs overexpressing CXCL1 (*CXCL1*^*OE*^), TAMs with a control short hairpin RNA (TAMs shCtrl), and TAMs with CXCL1 knockdown (TAMs *CXCL1*^*KD*^). The results revealed that TAMs secreted higher levels of CXCL1 compared to breast cancer cells. In addition, the secretion of CXCL1 was significantly elevated in TAMs *CXCL1*^*OE*^ relative to the vector-transduced control, whereas it was markedly reduced in TAMs *CXCL1*^*KD*^ in comparison with shCtrl group (Supplementary Fig. 1A). CCK8 assays further indicated that TAMs with CXCL1 overexpression significantly enhanced TAMs-induced chemoresistance of MDA-MB-231 and MCF-7 cell lines. This effect was partially attenuated by the application of a CXCL1 neutralizing antibody (Supplementary Fig. 1B). Conversely, CXCL1 knockdown in TAMs markedly suppressed TAM-induced chemoresistance, which was reversed upon the addition of CXCL1 cytokine in both breast cancer cell lines (Supplementary Fig. 1C). Overall, these findings indicated that CXCL1 appeared to be a crucial factor in TAMs-CM to induce breast cancer chemoresistance and autophagy.

### The activation of autophagy by CXCL1 promoted breast cancer cell chemoresistance to paclitaxel

**Fig. 3:**
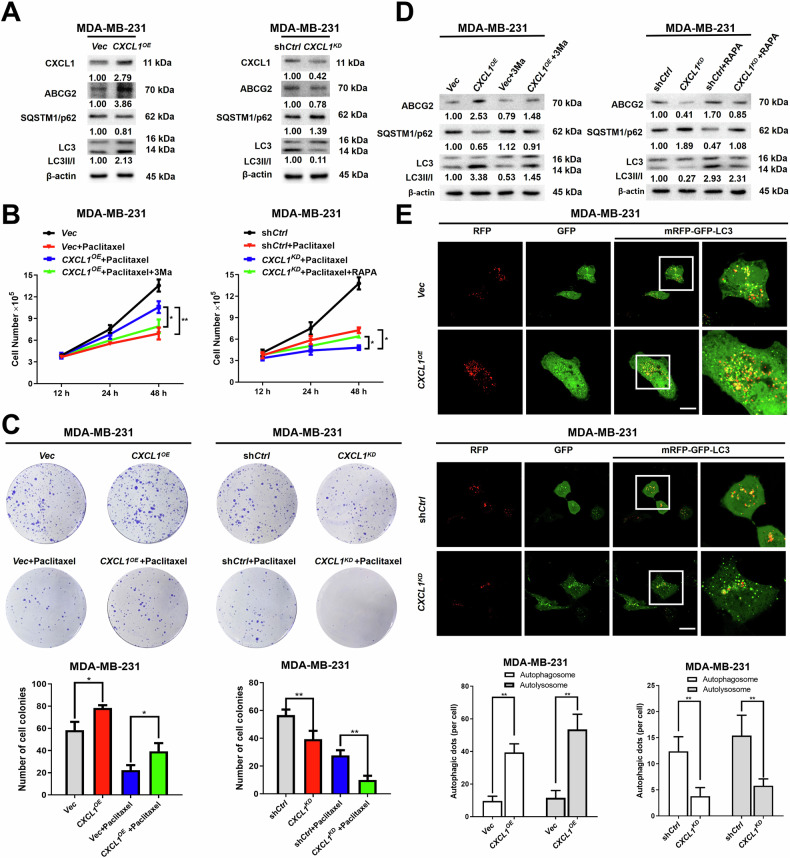
CXCL1 promoted breast cancer cell chemoresistance via activating autophagy. **A** Western blotting assay was conducted to assess the impact of *CXCL1* overexpression on autophagy- and chemoresistance-related proteins in MDA-MB-231 cells, as well as the inhibitory effect of *CXCL1* knockdown. **B** Cell counting and (**C**) colony formation assays were performed to evaluate the influence of *CXCL1* overexpression or downregulation on the chemosensitivity of MDA-MB-231 cells following the indicated treatment. The paclitaxel dosage was set at 50 nM, 3-Ma was used at a concentration of 10 mM, and RAPA dosage was 10 nM. **D** Western blotting assay was applied to evaluate the influence of *CXCL1* overexpression or knockdown on the expressions of ABCG2, SQSTM1/p62, and LC3 levels in MDA-MB-231 cells with the indicated autophagic modulators. 3-Ma was used at a concentration of 10 mM, and RAPA dosage was 10 nM. **E** mRFP-GFP-LC3 reporter assay was used to investigate the effect of *CXCL1* overexpression or knockdown on autophagy in MDA-MB-231 cells (*n* = 5, scale bars indicate 20 μm). Values are presented as Mean ± SD, *n* = 3 unless otherwise indicated, *P < 0.05, **P < 0.01.

To further validate the effect of CXCL1 on autophagy and chemoresistance in breast cancer, *CXCL1* was overexpressed (*CXCL1*^*OE*^) or silenced (*CXCL1*^*KD*^) by lentiviral transfection in MDA-MB-231 cells. Western blotting assay showed that the protein levels of ABCG2 and LC3-II/I increased, while SQSTM1/p62 decreased in response to *CXCL1* overexpression. By contrast, *CXCL1* knockdown reversed such process (Fig. 3A). Meanwhile, cell counting and colony formation assays further revealed that *CXCL1* overexpression reduced the chemosensitivity of MDA-MB-231 cells to paclitaxel, with partial reversal of this effect by 3-Ma. Conversely, *CXCL1* knockdown enhanced the cytotoxicity of paclitaxel, which was reversed by the autophagy agonist RAPA (Fig. 3B, C). Western blotting results also showed that 3-Ma partially mitigated the modulatory effects of *CXCL1* overexpression on LC3-II/I conversion, ABCG2, and SQSTM1/p62, while RAPA reversed the effects of *CXCL1* knockdown (Fig. 3D). Furthermore, MDA-MB-231 cells overexpressing *CXCL1* showed an increased presence of yellow and free red puncta, whereas cells lacking *CXCL1* displayed fewer autophagic puncta (Fig. 3E). Collectively, these data suggested that CXCL1 might serve as an upstream regulator of autophagy-related chemoresistance.

### Silencing CXCL1 enhanced the chemosensitivity of paclitaxel-resistant breast cancer cells via autophagy inhibition

**Fig. 4:**
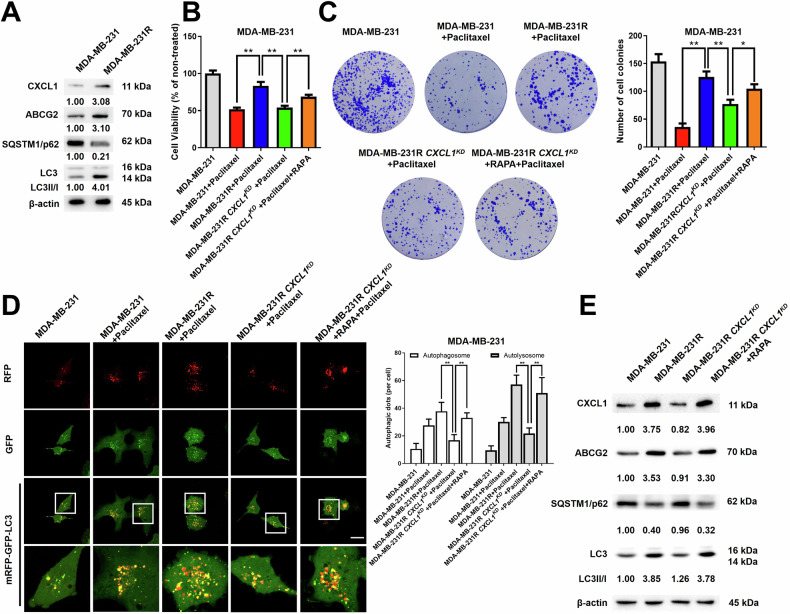
CXCL1 silence enhanced the chemosensitivity of paclitaxel-resistant breast cancer cells via autophagy inhibition. **A** Western blotting assay showed changes in CXCL1, ABCG2, SQSTM1/p62, and LC3 expression levels in MDA-MB-231R cells when compared to its parental cell line. **B** Cell counting and (**C**) colony formation assays were performed to explore the effects of CXCL1 silence on the chemosensitivity of MDA-MB-231R cells. The paclitaxel dosage was set at 50 nM, and the RAPA dosage was 10 nM. **D** mRFP-GFP-LC3 reporter assay was utilized to evaluate the impact of *CXCL1* knockdown on paclitaxel-induced autophagic flux in conjunction with 10 nM RAPA treatment (*n* = 5, scale bars indicate 20 μm). **E** Western blotting was used to evaluate the impact of *CXCL1* knockdown on the expressions of CXCL1, ABCG2, SQSTM1/p62, and LC3 levels with or without RAPA (10 nM) in MDA-MB-231R cells. Values are presented as Mean ± SD, *n* = 3 unless otherwise indicated, *P < 0.05, **P < 0.01.

A paclitaxel-resistant MDA-MB-231R cell line was established to thoroughly validate the role of CXCL1 in mediating chemoresistance. Western blotting assay showed that MDA-MB-231R cells expressed higher levels of CXCL1, LC3-II/I and ABCG2, but lower SQSTM1/p62 expression when compared to its parental cell line (Fig. 4A). Meanwhile, cell counting and colony formation assays were performed to assess the impact of CXCL1 on the chemosensitivity of MDA-MB-231R. The results indicated that *CXCL1* knockdown led to a significant increase in the cytotoxicity of paclitaxel on MDA-MB-231R cells, which was partially reversed by RAPA treatment (Fig. 4B, C). Furthermore, the mRFP-GFP-LC3 assay showed that CXCL1 suppression decreased yellow and free red puncta of MDA-MB-231R cells, with partial reversal by RAPA administration (Fig. 4D). In addition, western blotting results demonstrated that *CXCL1* knockdown in MDA-MB-231R cells significantly decreased the levels of ABCG2 and LC3-II/I, while increasing the level of SQSTM1/p62 expression (Fig. 4E). Taken together, these findings suggested that CXCL1 also promoted autophagy and chemosensitivity in paclitaxel-resistant breast cancer cells.

### Identification of IGF1/IGF1R as a downstream signaling for CXCL1-induced autophagy

**Fig. 5:**
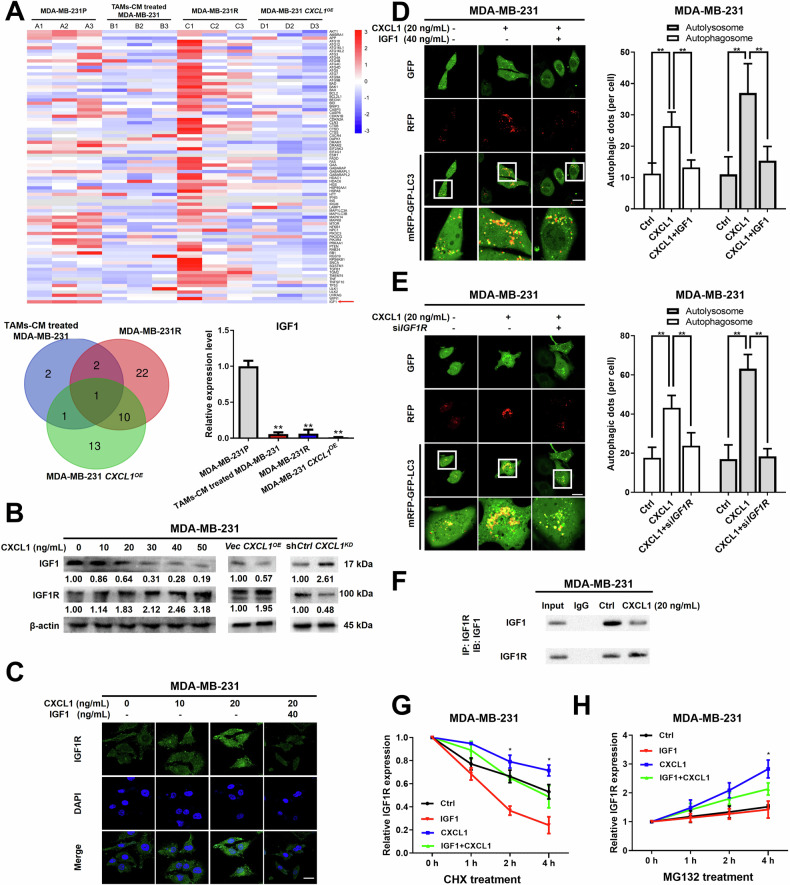
Identification of IGF1/IGF1R as a downstream signaling for CXCL1-induced autophagy. **A** The heatmap and Venn diagram analyses were conducted using data from a high-throughput quantitative PCR chip to evaluate differences among the indicated four groups, with a focus on IGF1 as the primary target. **B** Western blotting analysis was performed to evaluate the impact of varying concentrations of CXCL1 (0–50 ng/mL), *CXCL1* overexpression, or *CXCL1* knockdown on the expression levels of IGF1 and its receptor IGF1R in MDA-MB-231 cells. **C** Immunofluorescence was utilized to examine the expression and distribution of IGF1R with 20 ng/mL CXCL1 alone or its combination with 40 ng/mL IGF1 (scale bars indicate 20 μm). **D**, **E** mRFP-GFP-LC3 reporter assay was conducted to investigate the effect of 40 ng/mL IGF1 or IGF1R silence on CXCL1-mediated autophagy in MDA-MB-231 cells (*n* = 5, scale bars indicate 20 μm). **F** CoIP analysis was conducted to assess the effect of CXCL1 treatment (20 ng/mL) on protein interaction between IGF1 and IGF1R in MDA-MB-231 cells. **G**, **H** Western blotting assay was utilized to examine whether the change of IGF1R expression was affected by proteasome degradation. CXCL1 dose was 20 ng/mL, IGF1 dose was 40 ng/mL, CHX dose was 10 μM, and MG132 dose was 10 μM. Values are presented as Mean ± SD, *n* = 3 unless otherwise indicated, *P < 0.05, **P < 0.01.

To reveal the molecular mechanism underlying CXCL1-induced autophagy, a high-throughput quantitative PCR chip was applied to compare the levels of autophagy-related genes among the four groups of MDA-MB-231 parental (MDA-MB-231P) cells, TAMs-CM treated MDA-MB-231 cells, MDA-MB-231-resistant cells (MDA-MB-231R) cells, as well as MDA-MB-231 *CXCL1* overexpressing (MDA-MB-231 *CXCL1*^*OE*^) cells. The heatmap and the venn diagram identified IGF1 as the central gene controlling autophagy through the intersection of the aforementioned groups (Fig. 5A). Moreover, increasing CXCL1 levels through either CXCL1 chemokine or its plasmid constitutively expressing CXCL1 resulted in a reduction of IGF1 expression and an increase in IGF1R expression, the receptor for IGF1. Conversely, knockdown of *CXCL1* had the opposite effect (Fig. 5B). Immunofluorescence analysis further demonstrated that CXCL1 dose-dependently enhanced IGF1R expression in MDA-MB-231 cells, with partial reversal of this effect observed upon administration of IGF1 (Fig. 5C). In addition, treatment with CXCL1 was found to enhance the autophagic flux of MDA-MB-231 cells, with partial attenuation of this effect by IGF1 (Fig. 5D). To further elucidate the impact of IGF1R on CXCL1-induced autophagy, MDA-MB-231 cells were transfected with si*IGF1R*, resulting in a partial inhibition of CXCL1-induced autophagic flux (Fig. 5E). Moreover, CoIP results revealed that CXCL1 treatment led to a decrease in the interaction between IGF1 and IGF1R in MDA-MB-231 cells (Fig. 5F). Subsequent analysis focused on determining whether alterations in IGF1R expression were linked to the proteasome degradation pathway. Our findings revealed that CXCL1 significantly attenuated the rate of IGF1R protein degradation induced by CHX, with this effect being counteracted by IGF1 treatment. Since the ubiquitin-proteasome pathway plays a major role in protein degradation, cells were then treated with MG132, an inhibitor of the ubiquitin-proteasome pathway. The results demonstrated that CXCL1 prompted a more rapid accumulation of IGF1R in the presence of MG132 than that in the control group, while IGF1 administration partially suppressed the effects (Fig. 5G, H). To investigate the potential inhibitory effect of CXCL1 on the ubiquitination and degradation of IGF1R, the influence of *CXCL1* on IGF1R expression in MDA-MB-231 cells was then assessed. Western blotting analysis suggested that increased CXCL1 expression led to a notable reduction in ubiquitinated proteins and a rise in IGF1R expression in MDA-MB-231 cells. Meanwhile, the contrasting effects were observed in *CXCL1* knockdown cells (Supplementary Fig. 2A). VHL (Von Hippel-Lindau), an E3 ubiquitin ligase, could mediate the ubiquitination and degradation of numerous oncoproteins [29]. A CoIP experiment was conducted to explore the potential role of CXCL1 in modulating IGF1R expression of MDA-MB-231 cells through VHL modulation. It was shown that CXCL1 decreased VHL binding with IGF1R in MDA-MB-231 cells, while *CXCL1* knockdown increased VHL binding with IGF1R (Supplementary Fig. 2B). In addition, western blotting analysis demonstrated that *CXCL1* knockdown was associated with reduced expression of IGF1R and elevated levels of ubiquitylation, whereas si*VHL* treatment partially attenuated IGF1R ubiquitination in *CXCL1* knockdown MDA-MB-231 cells (Supplementary Fig. 2C). Overall, these data suggested that CXCL1 might induce autophagy by regulating the IGF1/IGF1R signaling in breast cancer.

### CXCL1 induced autophagy through IGF1R/STAT3/HMGB1 signaling in breast cancer

**Fig. 6:**
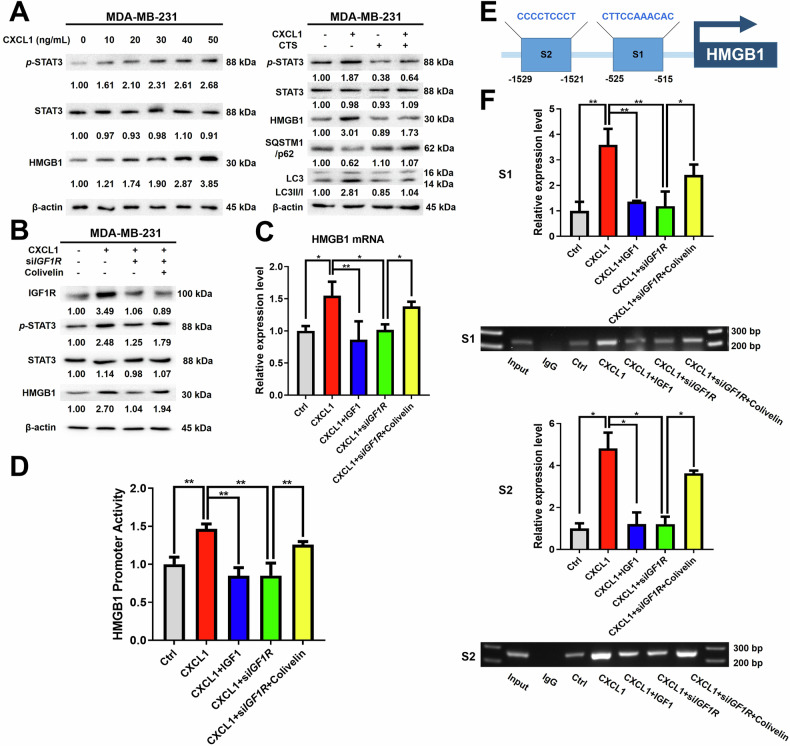
CXCL1 induced autophagy in breast cancer cell through the IGF1R/STAT3/HMGB1 signaling pathway. **A** Western blotting assay was conducted to detect the impact of varying CXCL1 (0–50 ng/mL) on the STAT3/HMGB1 pathway (left) and the involvement of STAT3 in the regulatory effects of CXCL1 on HMGB1 (right). STAT3 inhibitor CTS dose was set at 5 µM. **B** Western blotting assay was utilized to assess the relationship between IGF1R and STAT3 by conducting *IGF1R* knockdown and utilizing the STAT3 activator Colivelin (2 µM). **C** RT-*q*PCR and (**D**) dual-luciferase reporter gene assays were used to investigate the role of IGF1R/STAT3 axis in CXCL1-regulated transcriptional activity and mRNA expression of HMGB1. CXCL1 dose was 20 ng/mL, IGF1 dose was 40 ng/mL, and Colivelin dose was 2 µM. **E** A search of the Jaspar program and the hTFtarget database predicted two STAT3-binding sites within the HMGB1 promoter regions, including the sites from −1529 to −1521 and −525 to −521. **F** ChIP assay was conducted to reveal the direct binding of STAT3 to the promoter regions of HMGB1. The study also examined the impact of CXCL1 (20 ng/mL) on this binding activity, the partial inhibition of binding by IGF1 (40 ng/mL) and si*IGF1R*, and the restoration of si*IGF1R*’s effects by Colivelin (2 µM). Values are presented as Mean ± SD, *n* = 3, *P < 0.05, **P < 0.01.

Previous studies demonstrated that CXCL1 induced autophagy-mediated chemoresistance in breast cancer cells by modulating HMGB1 [21]. Herein, this study aimed to provide additional insight into the role of CXCL1 in the regulation of HMGB1. Firstly, it was found that HMGB1 transcription might be influenced by STAT3 through analysis of Cistrome Data Browser and subsequent bioinformatics analysis (see http://dbtoolkit.cistrome.org/?specie=hg38&distance=10k&factor=factor&keyword=chr13%3A30457915%3A30464326%3ANM_001313892%3AHMGB1). To investigate the role of STAT3 in the regulatory effects of CXCL1 on HMGB1, MDA-MB-231 cells were treated with CXCL1 chemokine in the presence or absence of STAT3 inhibitor cryptotanshinone (CTS). Western blotting assay showed the protein levels of *p*-STAT3 and HMGB1 were increased in the MDA-MB-231 cell line after 24 h of CXCL1 treatment, with CTS reversing the effects of CXCL1 on the expression of LC3, SQSTM1/p62, *p*-STAT3 and HMGB1 levels (Fig. 6A). It was also found that *IGF1R* knockdown inhibited CXCL1-induced *p*-STAT3 and HMGB1 upregulation. Nevertheless, the STAT3 activator Colivelin partially reversed the effects of si*IGF1R* but had no regulatory effect on IGF1R (Fig. 6B). The finding suggested that STAT3 might be a downstream target of IGF1R. Moreover, RT-*q*PCR and dual-Luciferase reporter gene assays showed that CXCL1 increased HMGB1 transcriptional activity and mRNA expression, while IGF1 or si*IGF1R* administration could inhibit the process. Interestingly, Colivelin treatment further reversed the effects of si*IGF1R* (Fig. 6C, D), suggesting that STAT3 might be a transcriptional factor for HMGB1. Therefore, we further predicted the STAT3-binding site in the HMGB1 promoter regions by searching the Jaspar program and the hTFtarget database. The results suggested the presence of two STAT3 binding sites within the HMGB1 promoter region, specifically at positions −1529 to −1521 and −525 to −521 (Fig. 6E). The result of ChIP assay indicated direct binding of STAT3 to both regions of HMGB1 promoter, with CXCL1 enhancing the binding activity, and either IGF1 or si*IGF1R* partially inhibiting it. Besides, colivelin was found to reverse the inhibitory effect of si*IGF1R* (Fig. 6F). In summary, these data suggested that CXCL1 could induce autophagy *via* IGF1R/STAT3/HMGB1 axis.

### TAMs/CXCL1 enhanced breast cancer chemoresistance by inducing autophagy in vivo

**Fig. 7:**
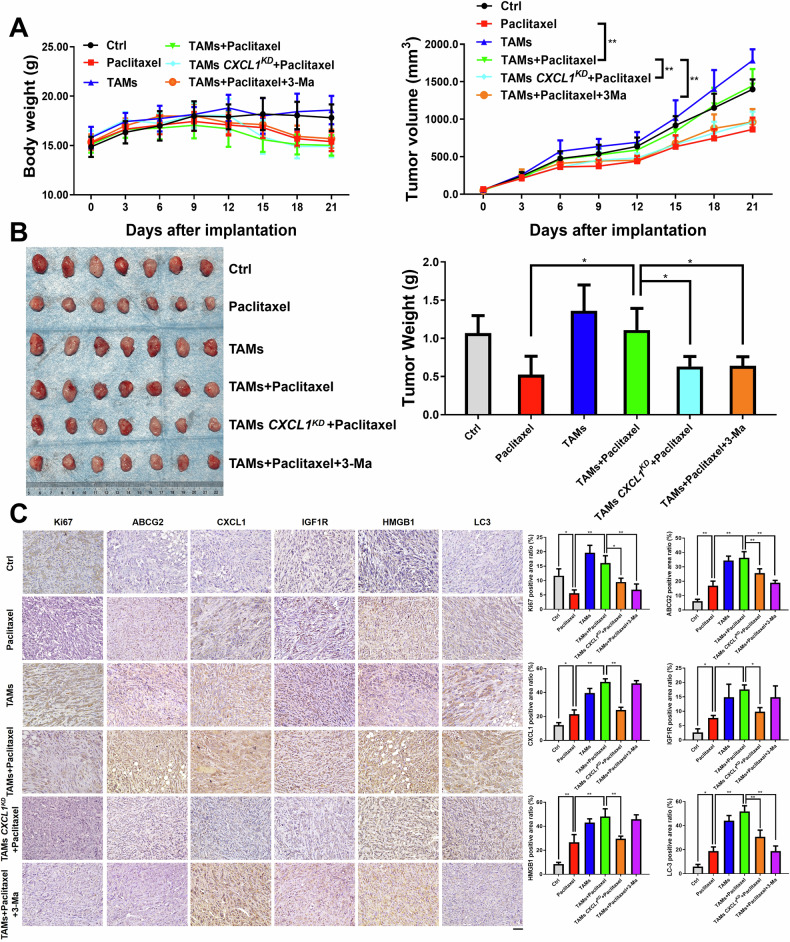
TAMs/CXCL1 enhanced breast cancer chemoresistance by inducing autophagy in vivo*.* **A**, **B** Tumor growth, body weight, and tumor weights were assessed for each group. Paclitaxel was administered intraperitoneally at a dosage of 10 mg/kg every three days, while the autophagy inhibitor 3-Ma was administered intraperitoneally at a dosage of 20 mg/kg daily. Values represent as Mean ± SD, *n* = 7, *P < 0.05, **P < 0.01, ^*#*^*P* < 0.05, ^*##*^*P* < 0.01. **C** Immunohistochemistry analysis was utilized to detect the expression levels of CXCL1, Ki67, IGF1R, HMGB1, LC3, and ABCG2 in tumor tissues among the indicated groups. Scale bars indicate 50 μm, values represent as Mean ± SD, *n* = 3, *P < 0.05, **P < 0.01.

For the in vivo study, Balb/c-nu-nu mice were utilized to establish breast cancer xenografts to investigate the impact of TAMs/CXCL1 on chemoresistance and autophagic process. The mice were categorized into six groups, including the Ctrl group, Paclitaxel group, TAMs group, TAMs + Paclitaxel group, TAMs *CXCL1*^*KD*^ + Paclitaxel group, and TAMs + Paclitaxel + 3-Ma group. TAMs or TAMs *CXCL1*^*KD*^ cells were mixed at a 3:1 ratio with MDA-MB-231 cancer cells and then injected into the mammary fat pads of Balb/c-nu-nu mice. It was found that the administration of paclitaxel at a dosage of 10 mg/kg/d resulted in an inhibition of breast cancer growth. However, TAMs co-injection (TAMs + Paclitaxel group) was found to conteract the inhibitory effects of paclitaxel. It was also observed that *CXCL1* knockdown in TAMs enhanced chemosensitivity of breast cancer to paclitaxel, and treatment with the autophagy inhibitor 3-Ma increased the chemosensitivity of paclitaxel when administered in conjunction with TAMs (Fig. 7A, B). Immunohistochemistry analysis further revealed that the TAMs + Paclitaxel group significantly increased the expression levels of CXCL1, Ki67, IGF1R, HMGB1, LC3, and ABCG2 compared to the paclitaxel alone group. In addition, either TAMs *CXCL1* knockdown or 3-Ma treatment led to decreased levels of Ki67, LC3, and ABCG2 compared with TAMs + Paclitaxel group (Fig. 7C). Collectively, these finding provided in vivo evidence supporting that TAMs increase paclitaxel chemoresistance by inducing CXCL1-mediated autophagy in breast cancer.

### Clinical significance of CXCL1 in predicting breast cancer prognosis and its correlation with IGF1R/HMGB1 signaling

**Fig. 8:**
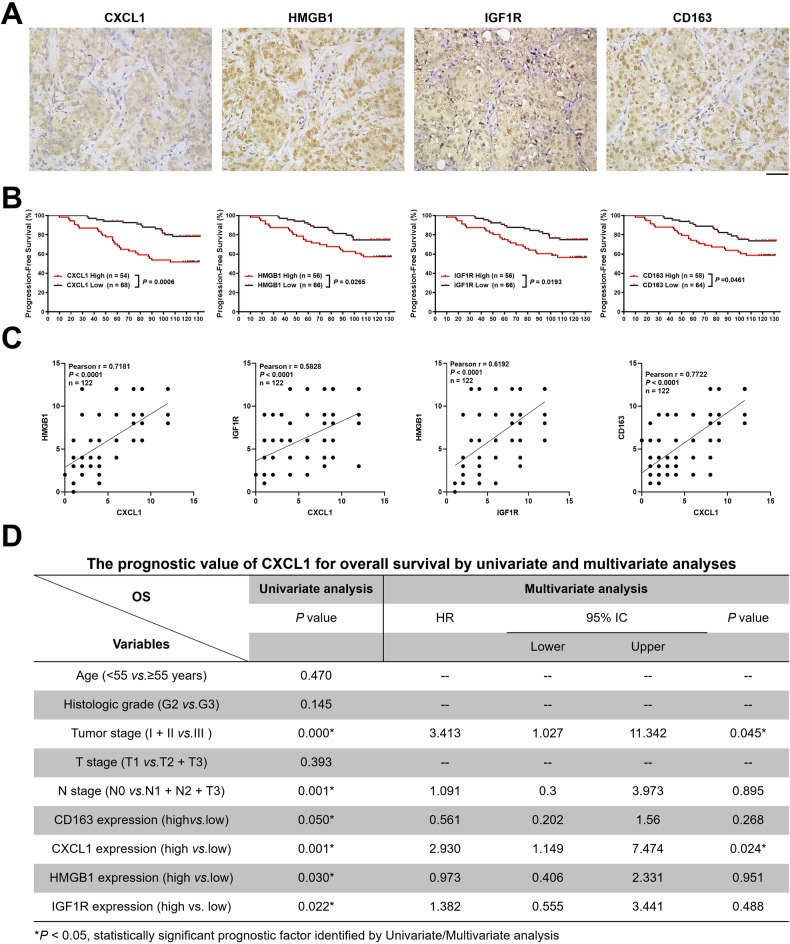
Clinical significance of CXCL1 in predicting breast cancer prognosis and its correlation with IGF1R/HMGB1 signaling. **A** Representative immunohistochemistry images demonstrating the expressions of CXCL1, HMGB1, IGF1R, and CD163 (scale bars indicate 50 μm). **B** Survival analysis indicated a significant association between poor PFS and elevated levels of CXCL1, HMGB1, IGF1R, or CD163 (*n* = 122). **C** A Pearson correlation analysis was conducted in tissue microarray samples (*n* = 122) to determine the correlation between CXCL1 expression and the expressions of HMGB1, IGF1R, and ICD163, respectively. **D** The prognostic value of CXCL1 for overall survival by univariate and multivariate analyses suggesting that CXCL1 expression as independent risk factor influencing breast cancer prognosis.

Breast cancer tissues in TMA were utilized to investigate the clinical significance of CXCL1 and its relevance with HMGB1, IGF1R, and CD163 expressions. The representative immunohistochemistry images of CXCL1, HMGB1, IGF1R as well as CD163 expressions were shown in Fig. 8A. Survival analysis further revealed that the poor PFS was correlated with high expression of CXCL1 (*P* = 0.0006), HMGB1 (*P* = 0.0265), IGF1R (*P* = 0.0193), and CD163 (*P* = 0.0461) (Fig. 8B). Meanwhile, it was found that HMGB1, IGF1R and CD163 expressions were strongly correlated with CXCL1 expression, as evidenced by a Pearson correlation analysis (Fig. 8C). Moreover, univariate COX analysis showed that the factors including tumor stage, N stage, CD163 expression, CXCL1 expression, HMGB1 expression, and IGF1R expression were significantly associated with breast cancer prognosis. Multivariate COX regression analysis further suggested that tumor stage and CXCL1 expression were independent risk factors determining breast cancer prognosis (Fig. 8D). Therefore, these results demonstrated that TAMs/CXCL1/IGF1R/HMGB1 signaling was closely associated with breast cancer prognosis.

## Discussion

Chemotherapy is one of the standard therapeutic strategies for triple-negative breast cancer. However, chemoresistance still presents a major challenge in decreasing breast cancer recurrence and mortality worldwide. Therefore, molecular elucidation of the underlying targets and mechanisms underlying chemoresistance is essential for developing novel strategies to prolong patients’ survival. In this study, we demonstrated that TAMs/CXCL1 promoted breast cancer chemoresistance by activating autophagy in vitro and in vivo. The IGF1R/STAT3/HMGB1 axis was identified as the principal mechanism accounting for CXCL1-induced autophagy. Clinical investigation also revealed that CXCL1 was an independent risk factor for breast cancer prognosis and positively correlated with IGF1R and HMGB1 expression. These results suggested that TAMs/CXCL1 signaling would be essential for the regulation of autophagy to mediate breast cancer chemoresistance.

Chemoresistance is a complex process involving multiple targets and complicated mechanisms. Autophagy is one of the main stress-regulatory mechanisms and shows bidirectional function during chemotherapy treatment. For example, Cytarabine, a major acute myeloid leukemia treatment drug, was found to induce a protective autophagy, thereby impairing its anticancer efficacy and causing resistance. However, it was found that the autophagic inducer Dendrogenin A could potentiate Cytarabine’s effect in vitro and in vivo by activating lethal autophagy. This was due to the synergistic induction of excessive autophagy might ultimately lead to autophagic cell death [30]. Herein, we demonstrated that autophagy played a key role in TAMs-associated chemoresistance of breast cancer cells. The findings of our study were consistent with several other studies. It was reported that LncRNA H19 derived from TAMs could stabilize ULK1 expression by increasing the expression of LC3-II/I and decreasing SQSTM1/p62 levels, thereby activating bladder cancer cell autophagy [31]. Guo et al. found that TAMs in the TME of hepatocellular carcinoma (HCC) generated massive IL-17, which suppressed oxaliplatin-induced apoptosis by promoting molecular chaperone-mediated autophagy [32]. Besides, it was found that TAMs induced autophagy and chemoresistance of HCC cells, while si*ATG5* suppressed autophagy in HCC cells and increased the cytotoxicity of oxaliplatin. These results indicated that TAMs-induced autophagy in HCC cells might contribute to chemoresistance to oxaliplatin [33]. Therefore, suppression of TAMs-mediated autophagy might be a potential therapeutic strategy to improve the prognosis of cancer patients with chemoresistance.

A strong association between CXCL1 and cancer chemoresistance was also supported by numerous studies. The study by Han et al. revealed that A-kinase-interacting protein 1 could promote the chemoresistance of glioblastoma to temozolomide by activating CXCL1/NF-κB signaling [34]. Moreover, a high level of CXCL1 was associated with radiosensitivity and poor prognosis in glioblastoma patients, and CXCL1 silence could reduce the proliferation and radioresistance of glioblastoma cells [35]. Besides, CXCL1 could shape the tumor microenvironment to affect chemoresistance. The chemotherapeutic drugs induced cancer cells to release more CXCL1 in the TME, leading to the recruitment of MDSCs into tumors and increased S100A8/9 secretion, ultimately increasing cancer cell survival following chemotherapy [36]. In glioblastoma multiforme, CXCL1 neutralizing antibody was shown to block MDSCs migration and resulted in an increase in CD8^+^ T cells in the tumor. A combination of CXCL1 neutralizing antibody and temozolomide chemotherapy was more effective than chemotherapy alone in treating glioblastoma multiforme, thereby extending OS [37]. CXCR2 was an important receptor for CXCL1. It was discovered that breast cancer cells that survived chemotherapy and radiation therapy expressed higher levels of CXCR2. The chemotherapeutic drugs paclitaxel and doxorubicin seemed to have more toxic effects on CXCR2 was knocked down in vitro. In mouse breast cancer xenografts, *CXCR2* knockdown allowed paclitaxel-treated animals to develop stronger anti-cancer activity and fewer lung metastases [38]. Clinical data analysis showed that poor PFS was correlated with high expression of CXCR2 in breast cancer patients, and CXCR2 was an independent risk factor determining breast cancer prognosis. Further research indicated that CXCR2 could promote breast cancer chemoresistance by inhibiting AKT1 signaling and activating COX2 signaling [39]. The finding of Ghallab *et al*. indicated that CXCR2 and TGF-β signaling were highly expressed in human triple-negative breast cancer biopsies, and CXCR2 inhibitor AZD5069 abrogated doxorubicin-induced TGF-β upregulation and eliminated chemoresistance in triple-negative breast cancer cells [40]. Hence, targeting CXCL1/CXCR2 signaling has emerged as a pivotal strategy for improving the prognosis of breast cancer, with potential benefits for patients resistant to chemotherapy through the use of CXCL1/CXCR2 antagonists.

Previous studies suggested that CXCL1 could induce autophagy-mediated chemoresistance in breast cancer cells by regulating HMGB1 [21]. Herein, we further revealed that CXCL1 promotes HMGB1 expression *via* IGF1/IGF1R/STAT3 signaling. The canonical receptor for IGF1, known as IGF1R, is recognized for its role in regulating autophagy. It was also revealed that IGF1R might also be significant in the processes of carcinogenesis and chemoresistance in cancer cells. Kato et al. analyzed the expression of IGF-1R protein in tumor tissues of nearly 100 Japanese non-small cell lung cancer patients treated with gefitinib using IHC analysis. It was found that those with high expression of the IGF1R were more likely to be resistant to gefitinib therapy and had significantly shorter OS and PFS [41]. In a phase II clinical trial of advanced pancreatic cancer patients treated with IGF1R-directed monoclonal antibody MK-0646 combined with gemcitabine, high expression of IGF1 in the combined treatment group was found to be associated with reduced risk of disease progression or death [42]. It was reported that IGF1 could remarkably down-regulate IGF1R level by increasing its degradation through the ubiquitination-dependent proteasome pathway [43]. Several studies indicated that IGF1R inhibition affected autophagy differently in cancer cells. Through large-scale compound screening, picropodophyllotoxin was identified as an IGF1R inhibitor and capable of activating autophagy, which is key in improving chemotherapy-induced immunogenic cell death [44]. By contrast, Renna *et al*. found that IGF1R siRNA induced a reduction of autophagosomes in HeLa cells. si*IGF1R* was found to suppress mTORC2 signaling, finally leading to a decrease in PKC α/β activity, thereby reducing autophagosome precursor formation [45]. The reason for the inconsistent effect of IGF1R inhibitors and siRNA on autophagy might be due to the multiple targets of picropodophyllotoxin. In the present study, we also found that IGF1R siRNA could inhibit the autophagic flux, thereby confirming the regulatory effects of IGF1R on autophagy. STAT3 has been recognized as the canonical downstream signaling of IGF1R, and the IGF1R/STAT3 signaling axis has been associated with the development of cancer chemoresistance. It was reported that activation of the IGF1R/STAT3 pathway resulted in the upregulation of ALDH1, which in turn enhanced the stemness and radioresistance of non-small cell lung cancer [46]. Additionally, Zheng et al. found that IGF1R might contribute to gefitinib resistance in non-small cell lung cancer through the JAK/STAT3 signaling pathway [47]. Mechanistically, IGF1R underwent conformational changes and autophosphorylation, leading to the creation of docking sites for various adaptor proteins. For example, IGF1R was observed to recruit STAT3 through the adaptor protein RACK1, forming a multiprotein complex that activated the downstream gene transcription [48]. Our study also found that IGF1R induced STAT3 phosphorylation. leading to HMGB1 transcription, thus confirming IGF1R’s role as an upstream regulator of STAT3. Currently, the subcellular localization patterns of STAT3 were believed to have varying effects on autophagy. Nuclear STAT3 induced autophagy by affecting the transcription of autophagy-related genes (e.g. BCL2, BECN1, and HIF1α), while cytoplasmic STAT3 was reported to inhibit autophagy by interacting with autophagy-related signaling molecules such as FOXO3 [49]. A critical regulator of autophagy, HMGB1, was also shown to be regulated by STAT3. Wolfson et al. found that HMGB1 transcription might be modulated by STAT3, and STAT3 silence could eliminate HMGB1 expression in human lung endothelial cells induced by high amplitude cyclic stretch [50]. Herein, it was concluded that STAT3 bind with the promoter regions of HMGB1, which promoted autophagy-mediated chemoresistance. These findings highlighted the significance of STAT3/HMGB1 signaling in autophagy regulation and corresponding drug development.

Collectively, our research highlights the important role of TAMs/CXCL1 in mediating breast cancer chemoresistance through the regulation of autophagy, and proposes IGF1/IGF1R as a potential upstream signaling that governs autophagy *via* STAT3/HMGB1 activation. Targeting CXCL1-mediated autophagy may be one of the MDR reversal mechanisms in breast cancer.

## Supplementary information

Supplementary Figure 1

Supplementary Figure 2

Original images of blot

## Data Availability

The datasets generated during and/or analysed during the current study are available from the corresponding author on reasonable request.
