# Supplementary material for: Tumor-associated macrophages/C-X-C motif chemokine ligand 1 promotes breast cancer autophagy-mediated chemoresistance via IGF1R/STAT3/HMGB1 signaling
Source: Cell Death Dis. 2024 Oct 11;15(10):743. doi: 10.1038/s41419-024-07123-5 (PMC11470078; doi:10.1038/s41419-024-07123-5)
Supplement: Supplementary file 1 — Supplementary Figure 1 [file 41419_2024_7123_MOESM1_ESM.pdf]

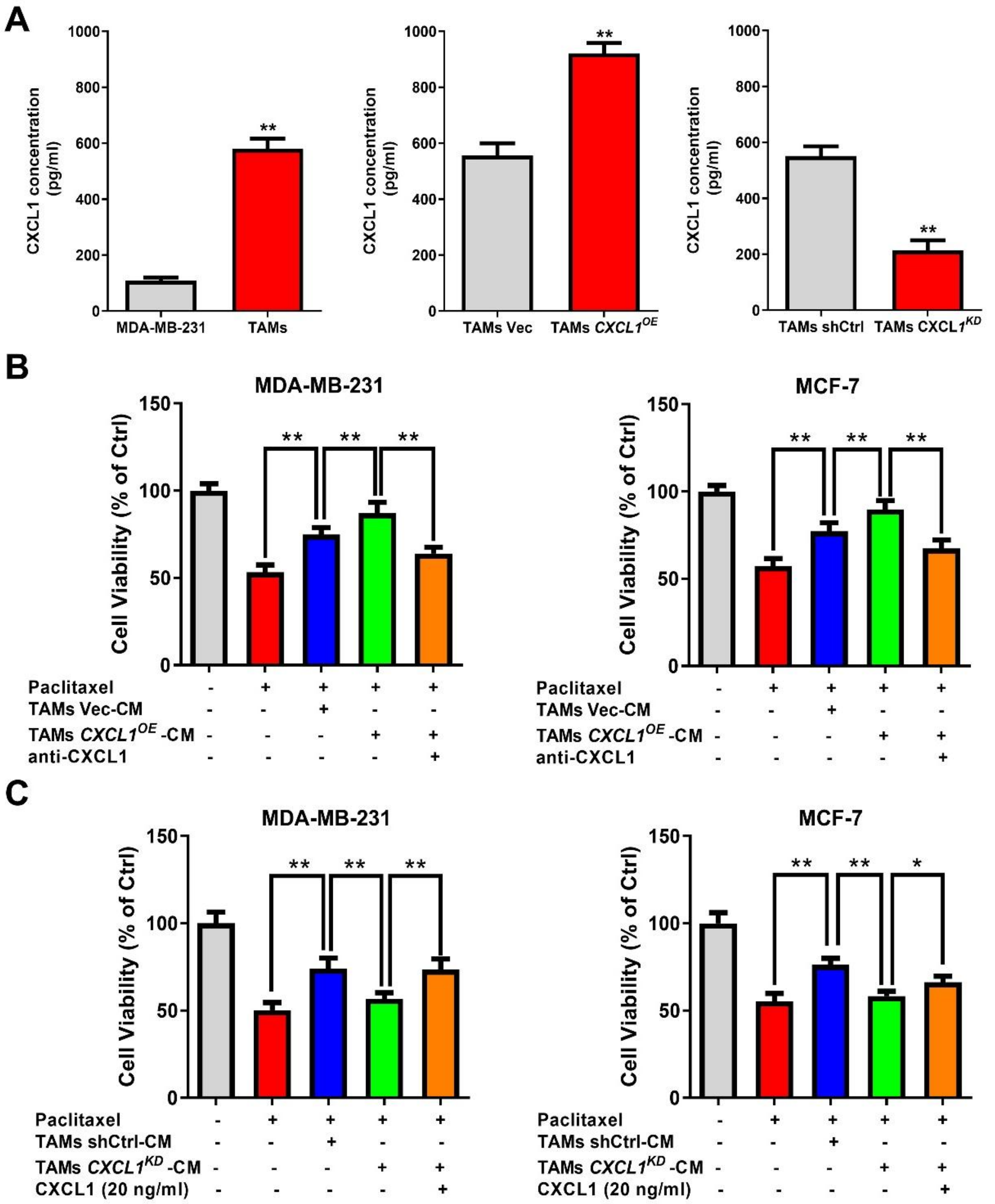

Supplementary Fig 1. The concentration of CXCL1 in supernatants derived from TAMs and MDA-MB-231 cells. (**A**) ELISA assays were utilized to detect the concentration of CXCL1 in supernatants derived from TAMs, MDA-MB-231 cells, TAMs Vec, TAMs $CXCL1^{OE}$, TAMs shCtrl as well as TAMs $CXCL1^{KD}$. Values are presented as Mean ± SD, n = 3, $^{**}P < 0.01$. (**B**) CCK8 assays were conducted to assess the impact of CXCL1 overexpression on TAMs-induced paclitaxel resistance in breast cancer cells, as well as the reversal effect of a CXCL1 neutralizing antibody (Mean ± SD, n = 6, $^{**}P < 0.01$). (**C**) The inhibitory effects of CXCL1 knockdown on TAMs-induced paclitaxel resistance in breast cancer cells were also evaluated using CCK8 assays (Mean ± SD, n = 6, $^{*}P < 0.05$, $^{**}P < 0.01$).
