# Supplementary material for: Tumor-associated macrophages/C-X-C motif chemokine ligand 1 promotes breast cancer autophagy-mediated chemoresistance via IGF1R/STAT3/HMGB1 signaling
Source: Cell Death Dis. 2024 Oct 11;15(10):743. doi: 10.1038/s41419-024-07123-5 (PMC11470078; doi:10.1038/s41419-024-07123-5)
Supplement: Supplementary file 2 — Supplementary Figure 2 [file 41419_2024_7123_MOESM2_ESM.pdf]

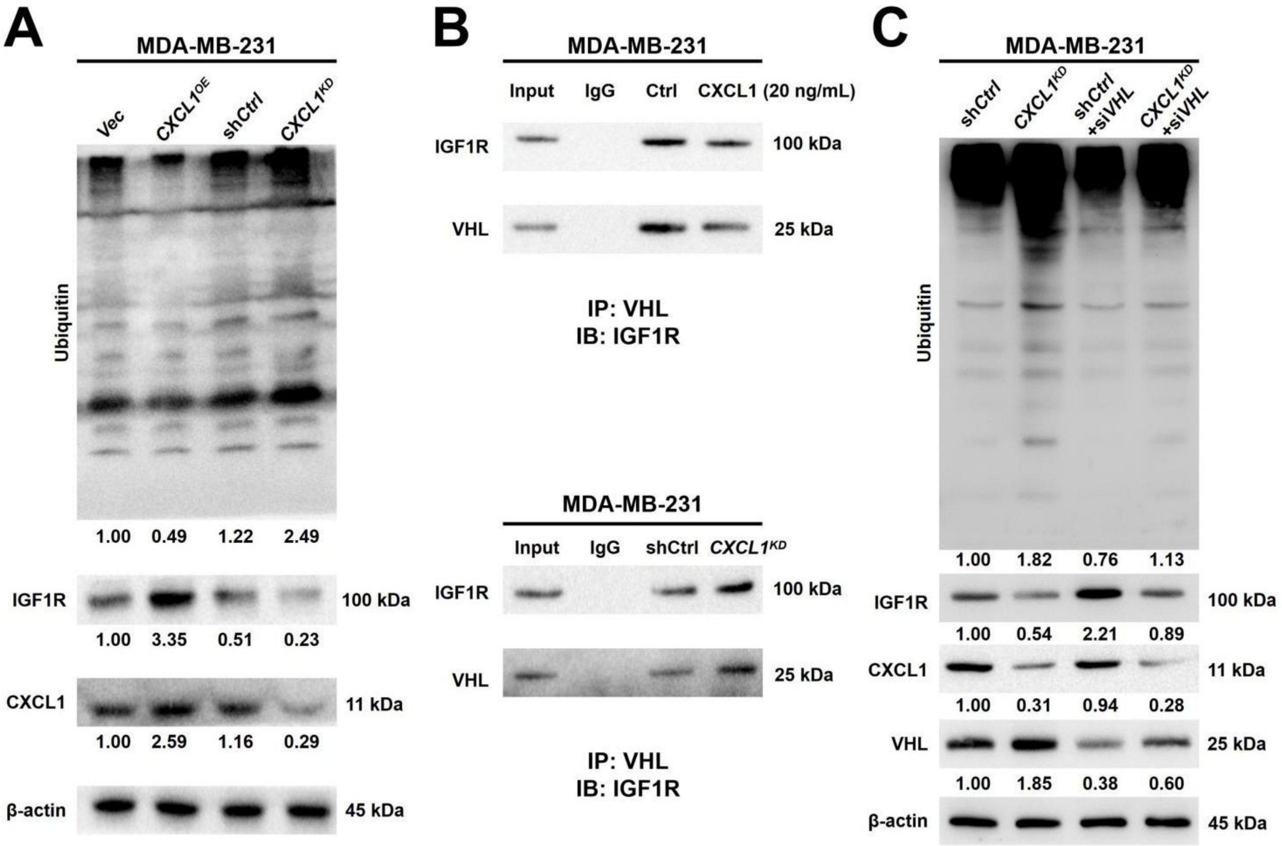

Supplementary Fig 2. CXCL1 inhibited VHL-mediated ubiquitination and degradation of IGF1R in MDA-MB-231 cells. (**A**) Western blotting assay was utilized to assess the effects of *CXCL1* overexpression or knockdown on the levels of ubiquitinated protein and IGF1R expression in MDA-MB-231 cells. (**B**) CoIP analysis was conducted to assess the effect of CXCL1 treatment (20 ng/mL) or *CXCL1* knockdown on protein interaction between VHL and IGF1R in MDA-MB-231 cells. IP: VHL, IB: IGF1R. (**C**) Western blotting analysis was utilized to examine the effect of *CXCL1* knockdown on the levels of IGF1R and ubiquitylation in MDA-MB-231 cells in the presnece of the specific siRNA targeting *VHL*.
