# Supplementary material for: Tumor-associated macrophages/C-X-C motif chemokine ligand 1 promotes breast cancer autophagy-mediated chemoresistance via IGF1R/STAT3/HMGB1 signaling
Source: Cell Death Dis. 2024 Oct 11;15(10):743. doi: 10.1038/s41419-024-07123-5 (PMC11470078; doi:10.1038/s41419-024-07123-5)

# Figure 1C MDA-MB-231

ABCG2

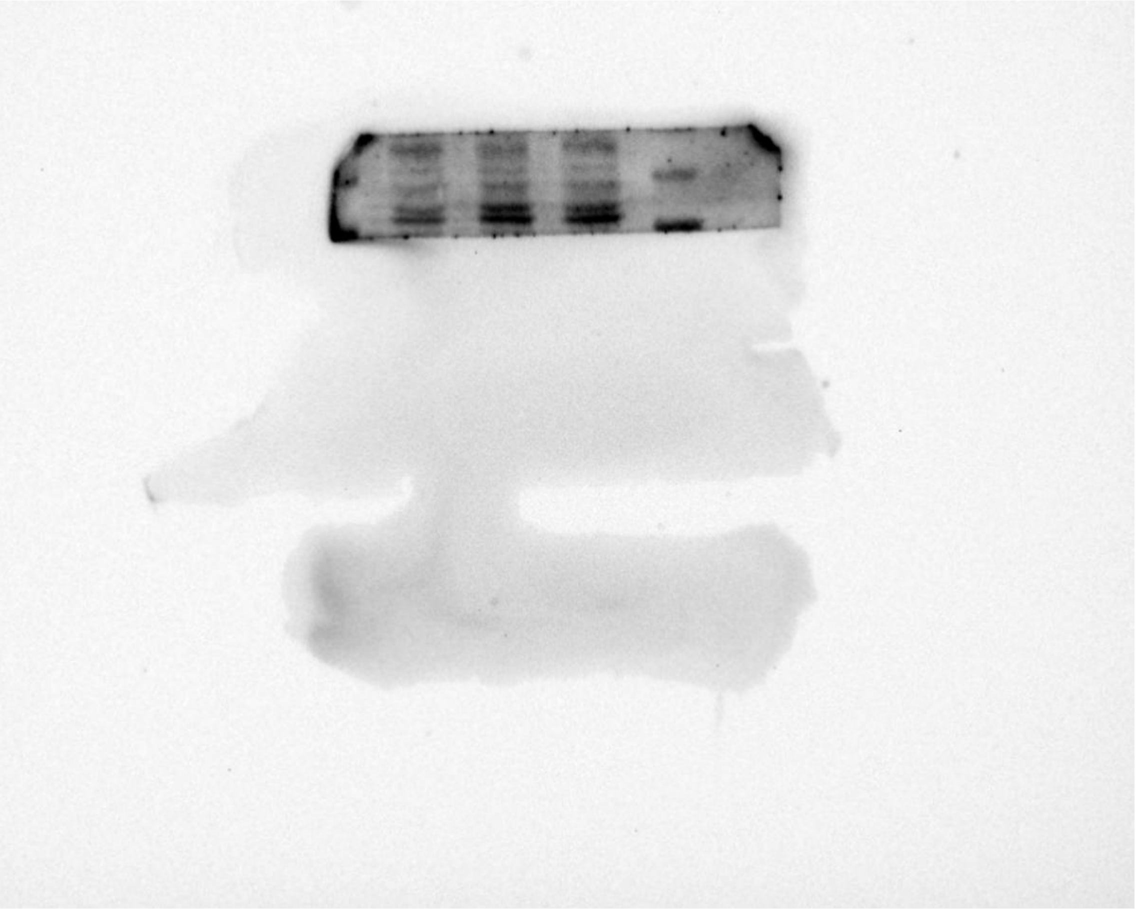

P62

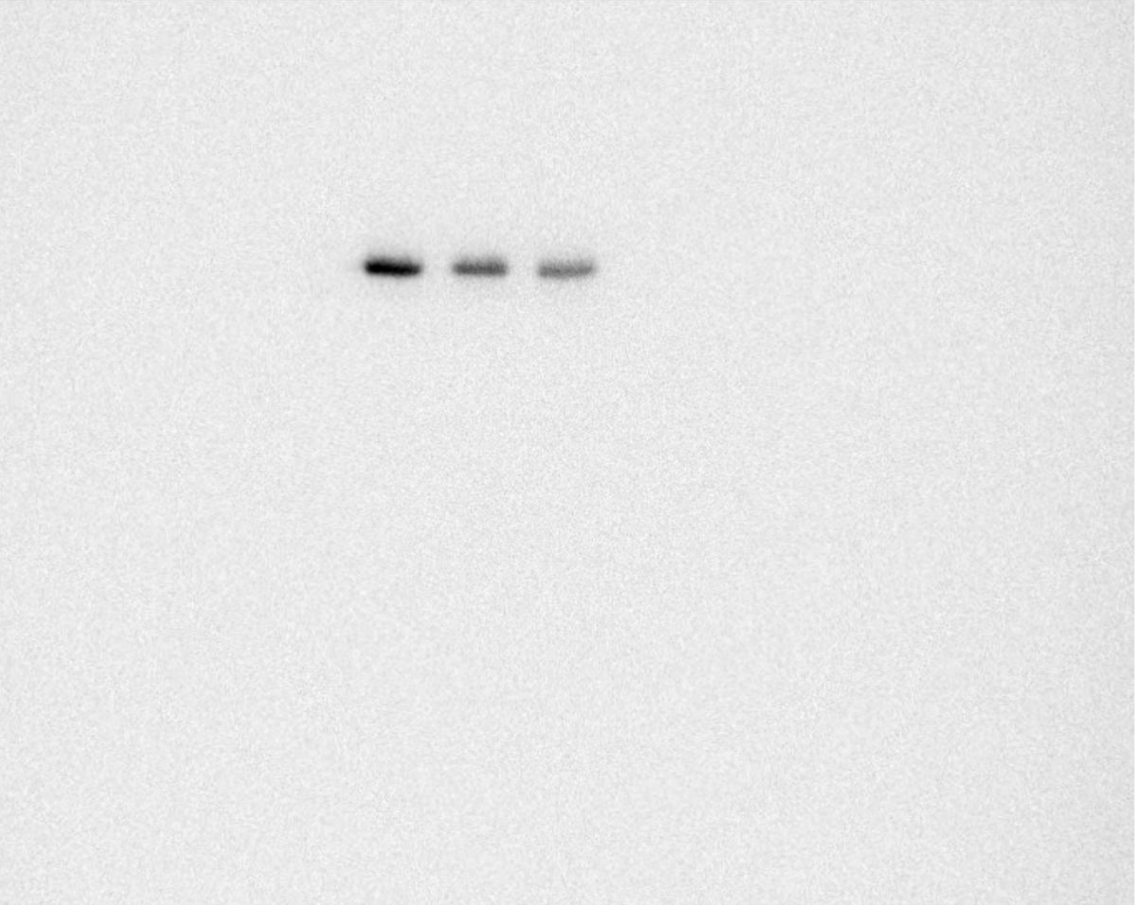

Figure 1C MDA-MB-231

β-actin

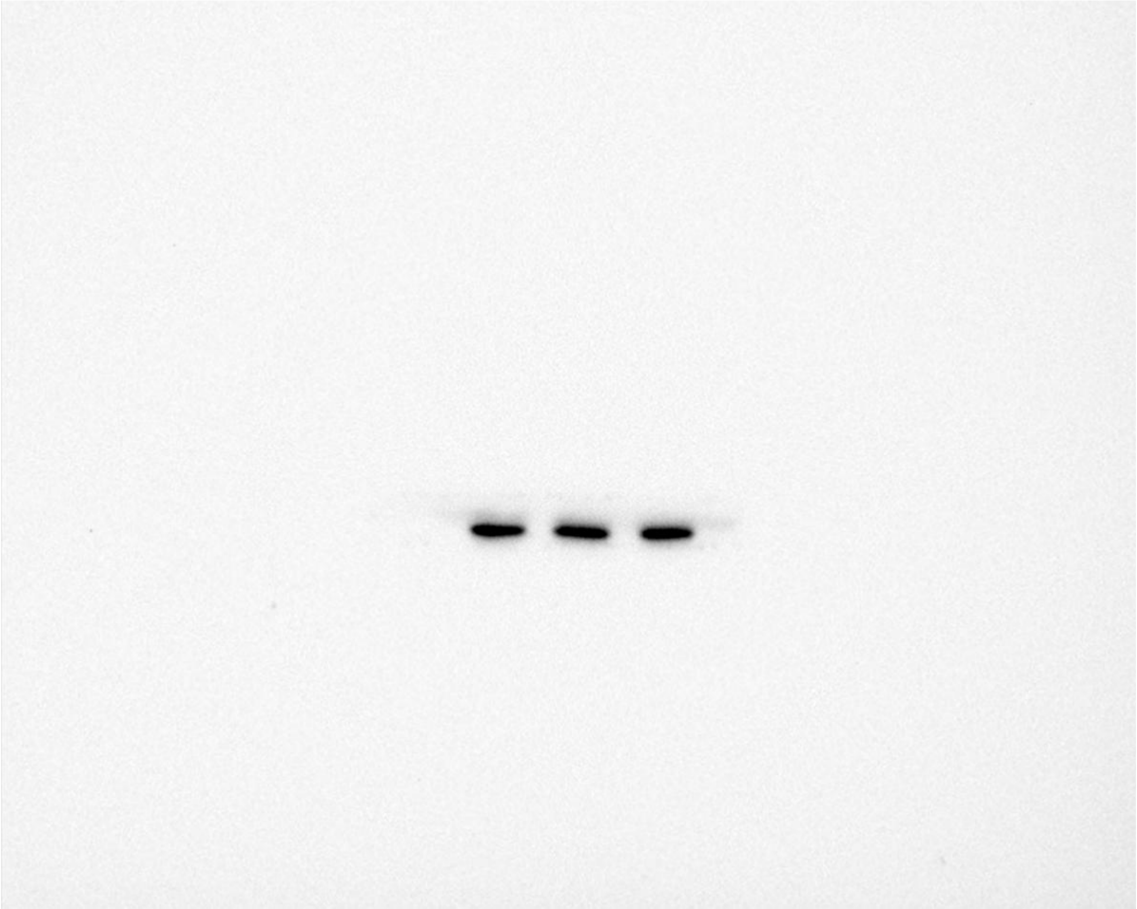

LC-3

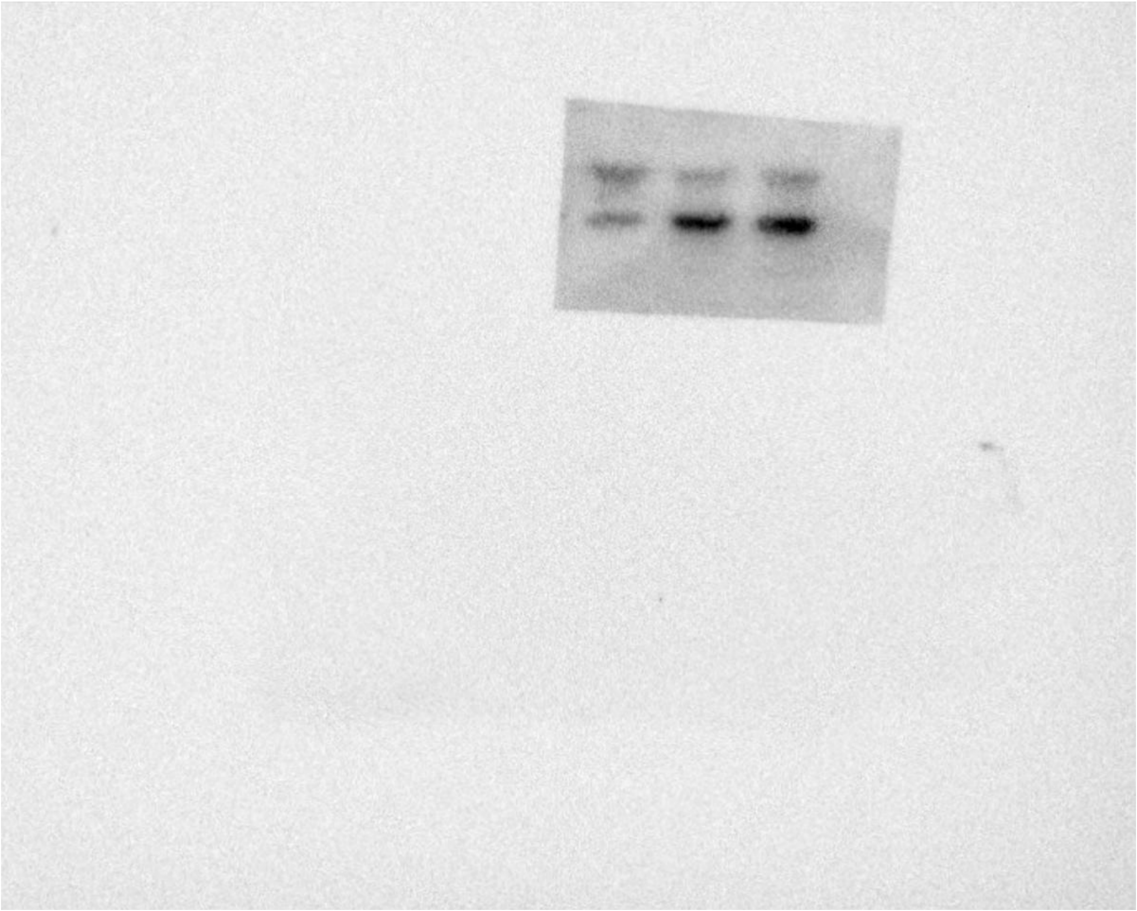

Figure 1C MCF-7

ABCG2

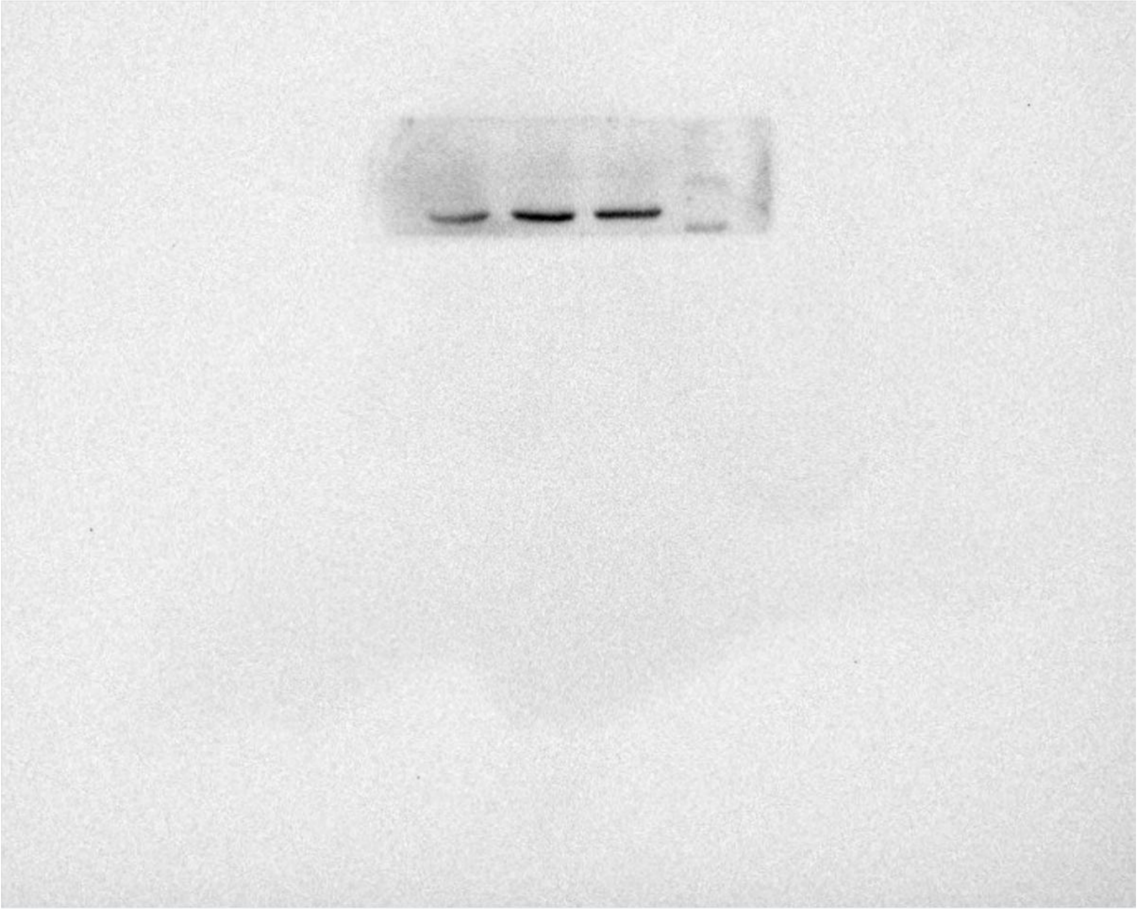

P62

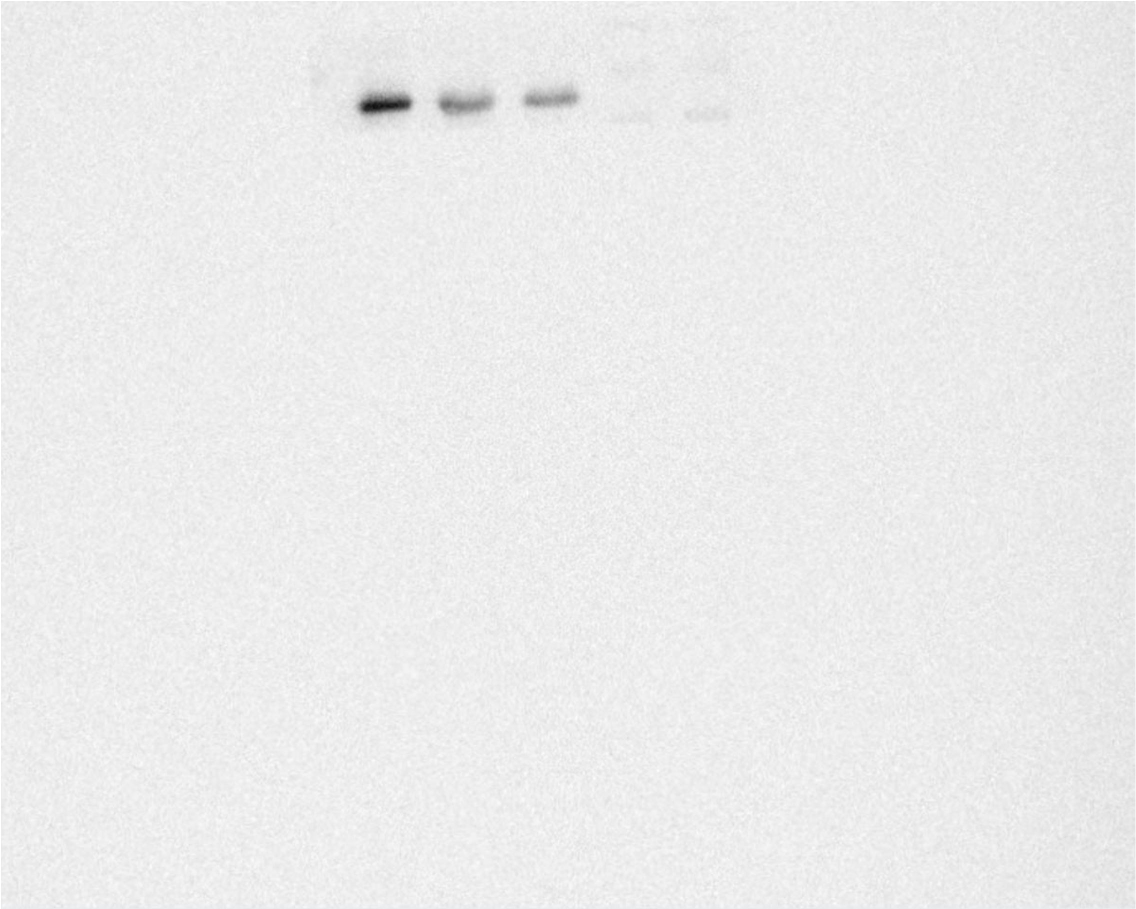

**Figure 1C MCF-7**

β-actin

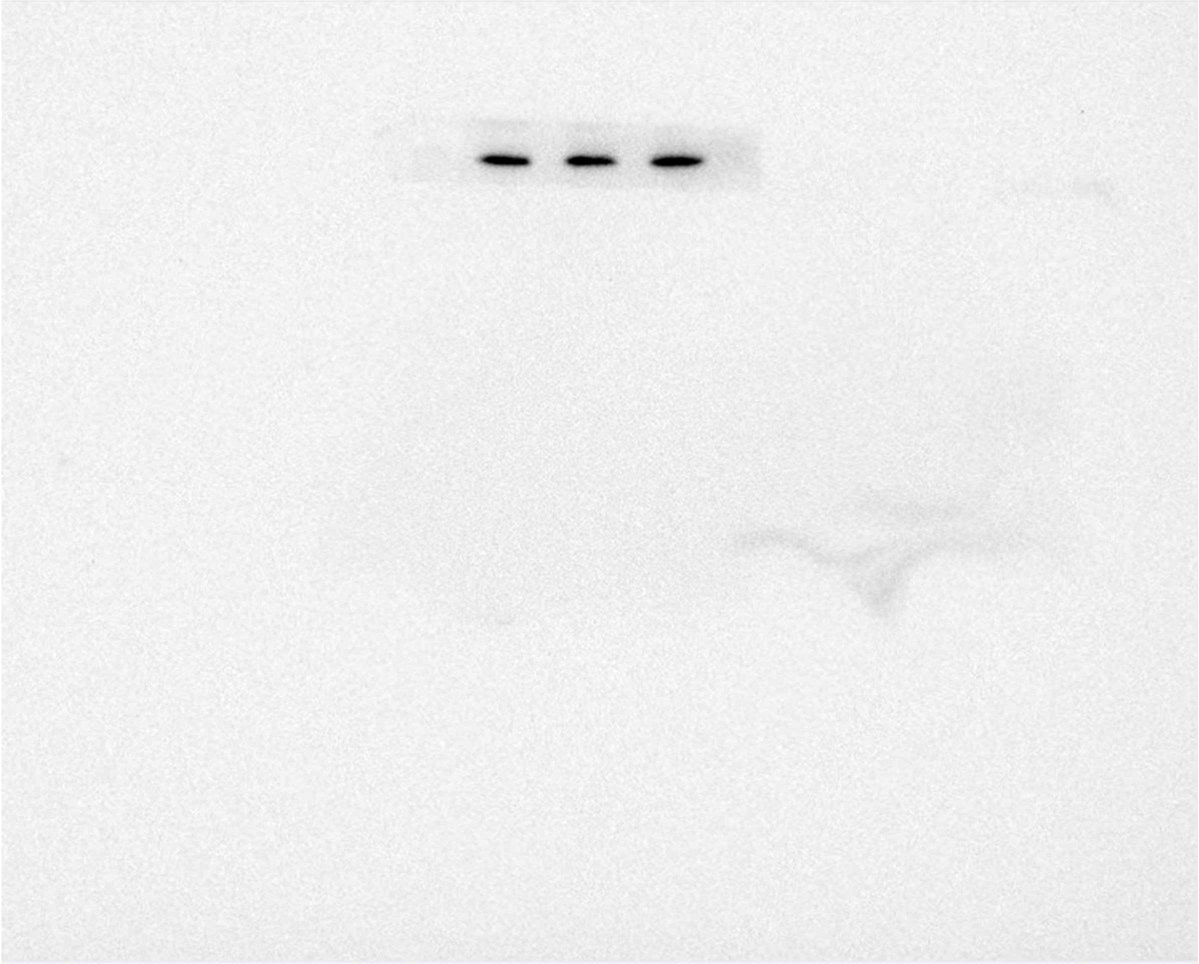

LC-3

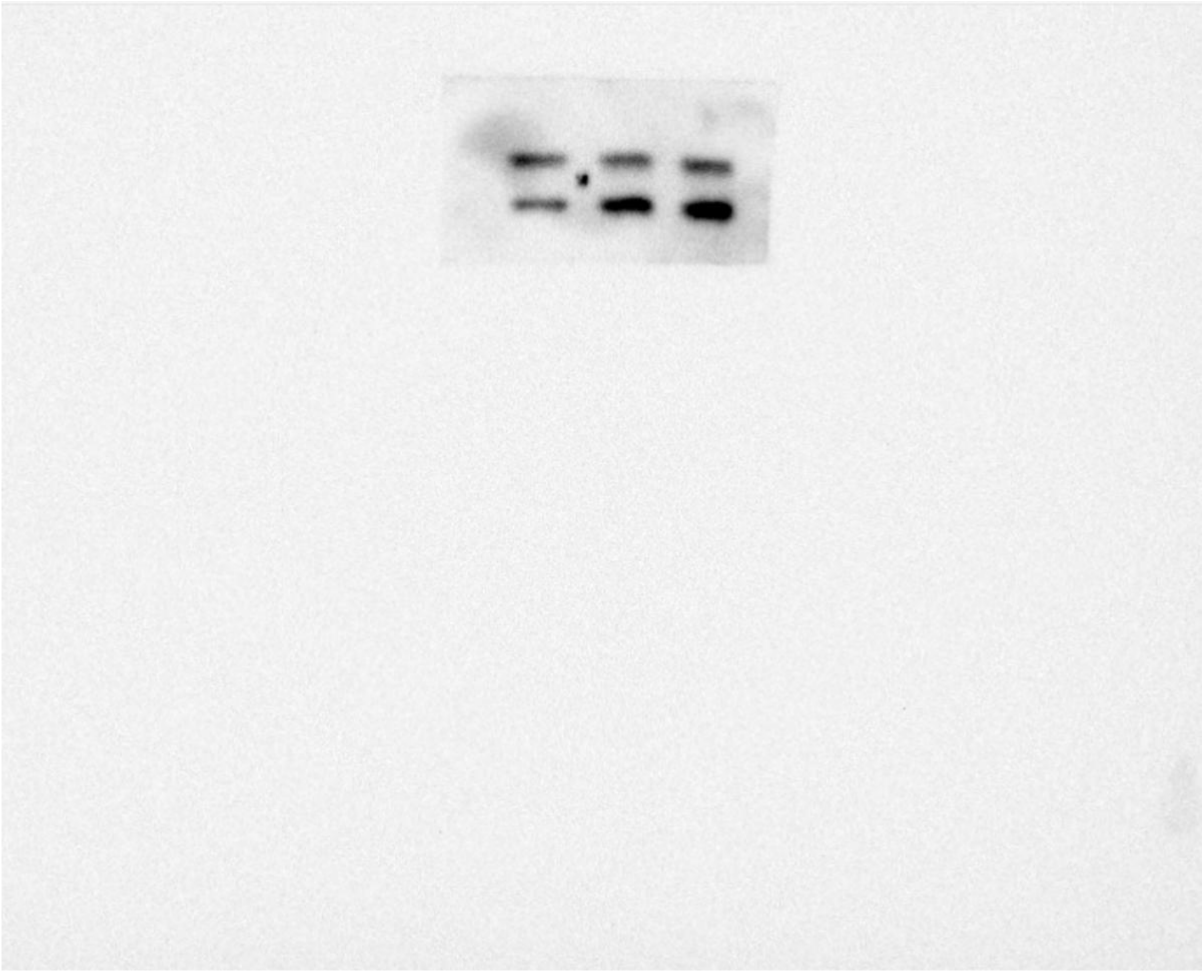

# Figure 1D MDA-MB-231

ABCG2

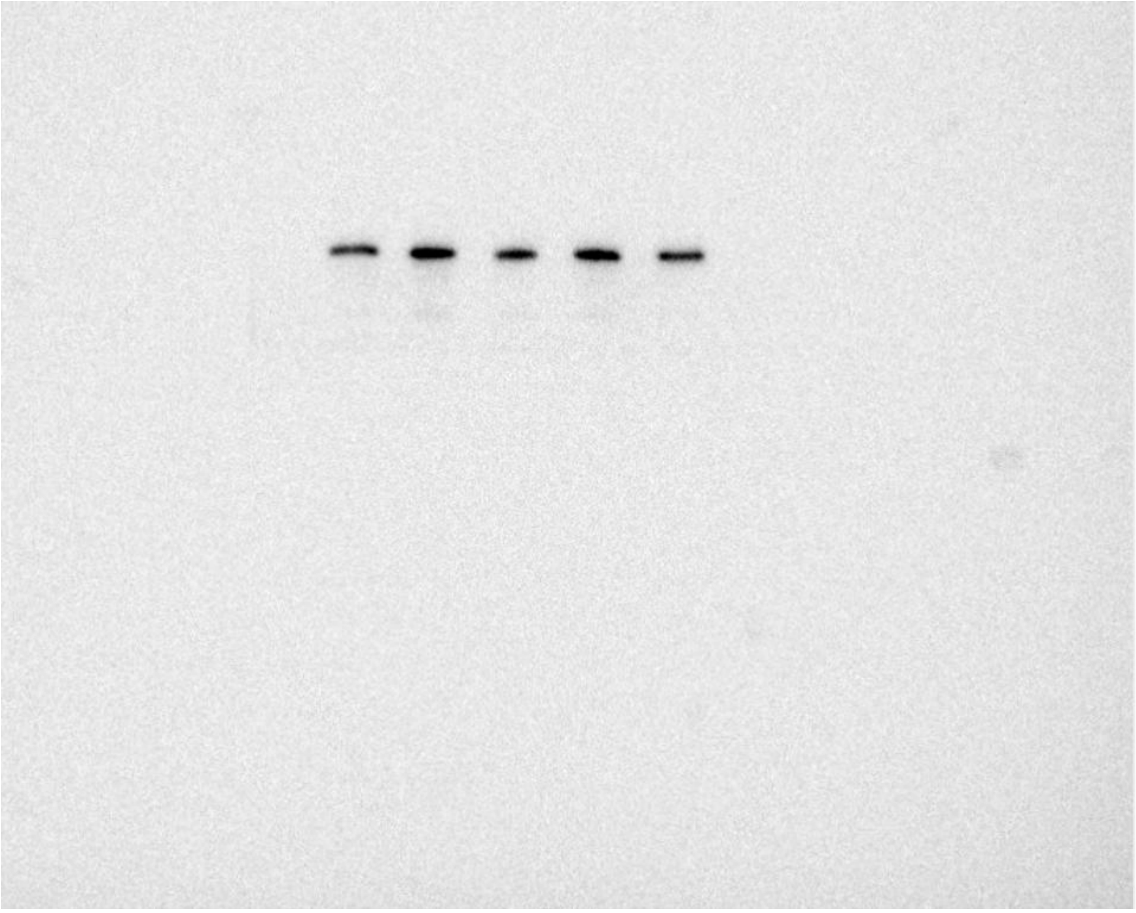

P62

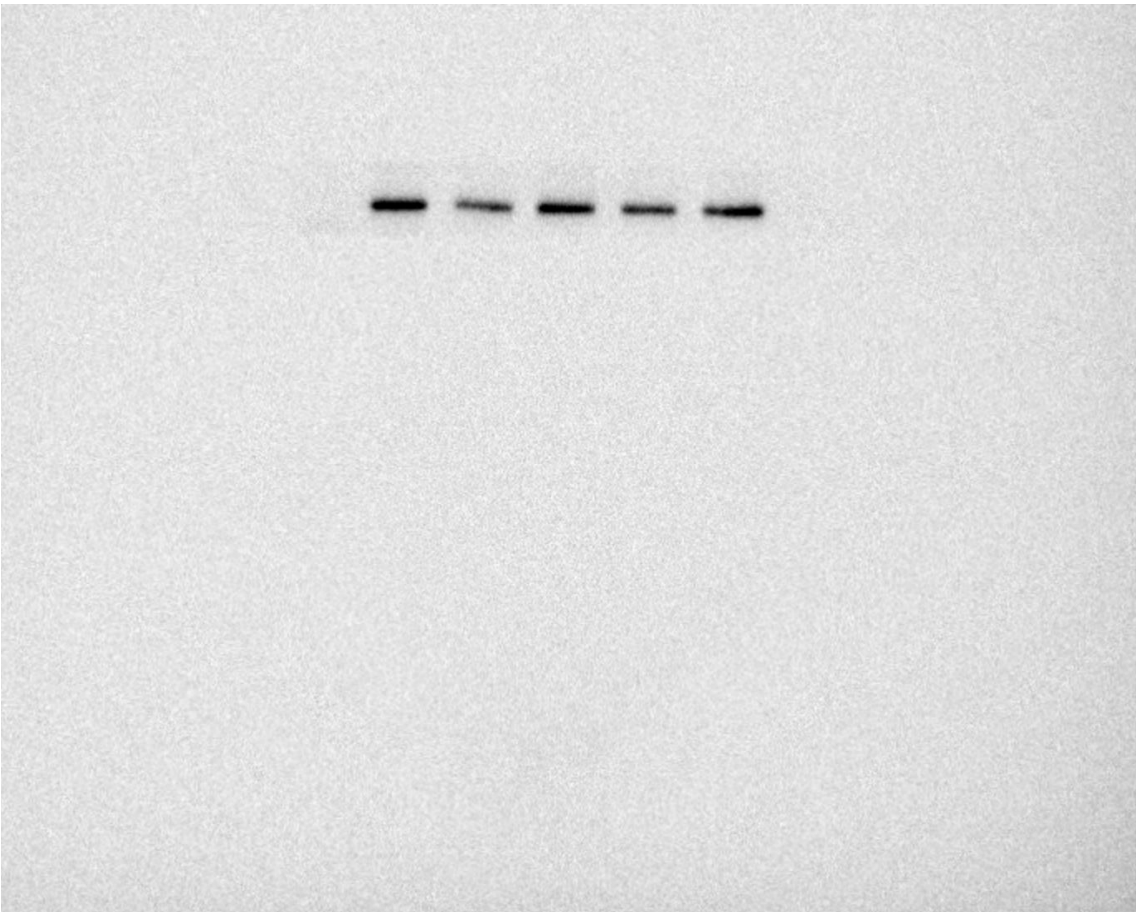

# Figure 1D MDA-MB-231

β-actin

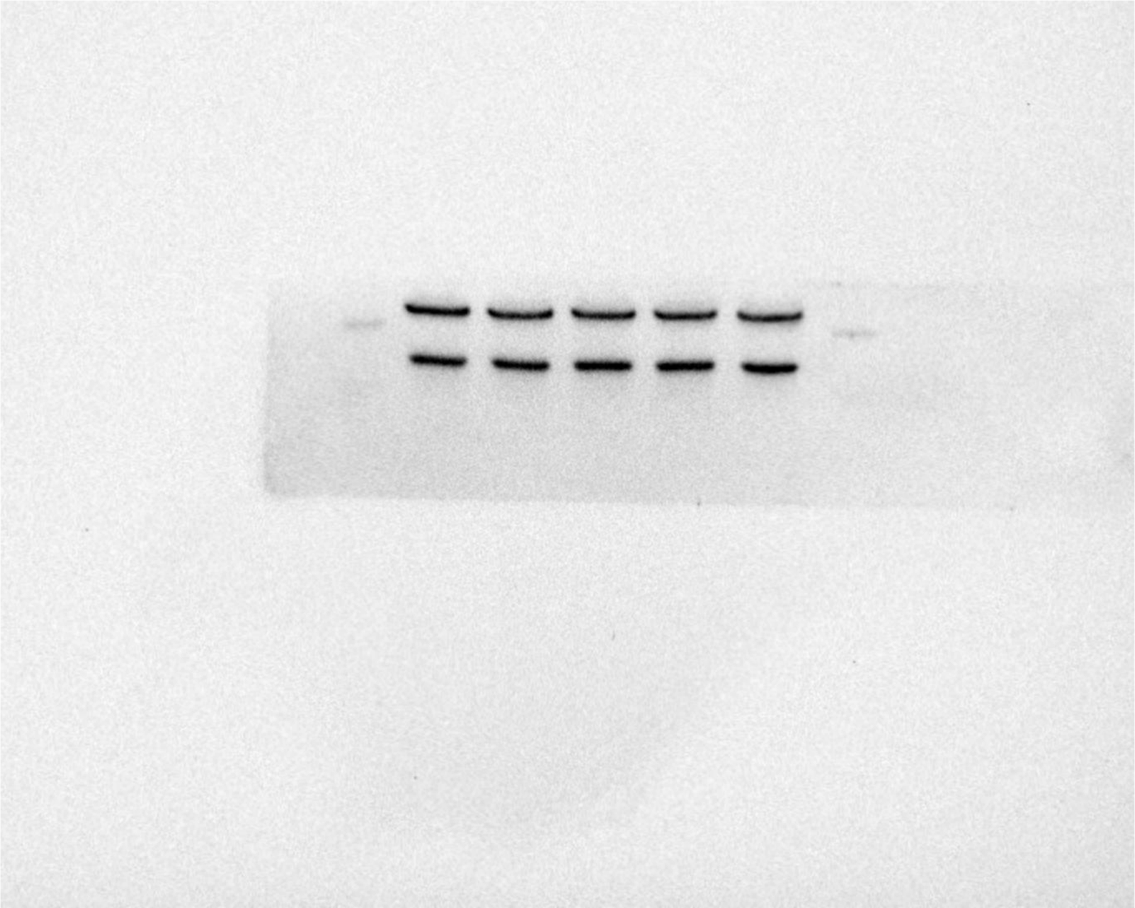

LC3

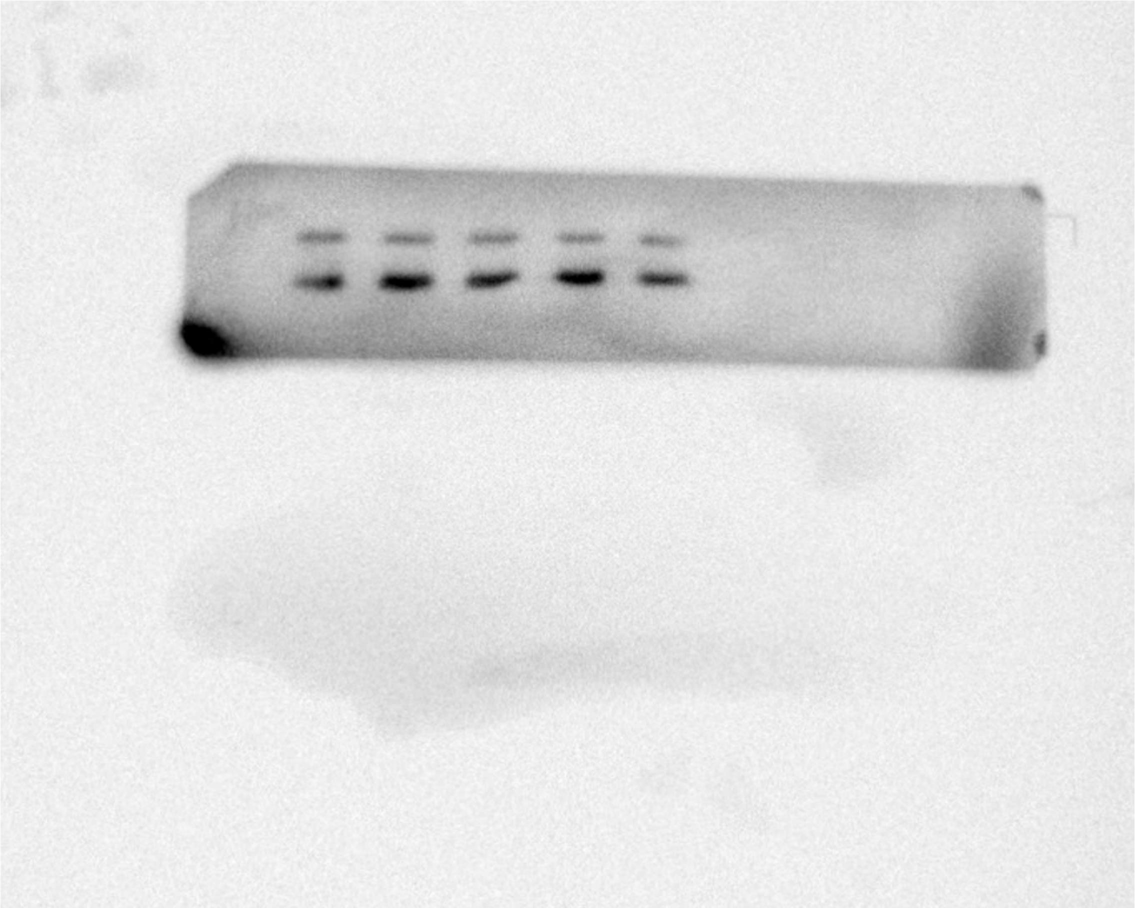

**Figure 1D MCF-7**

ABCG2

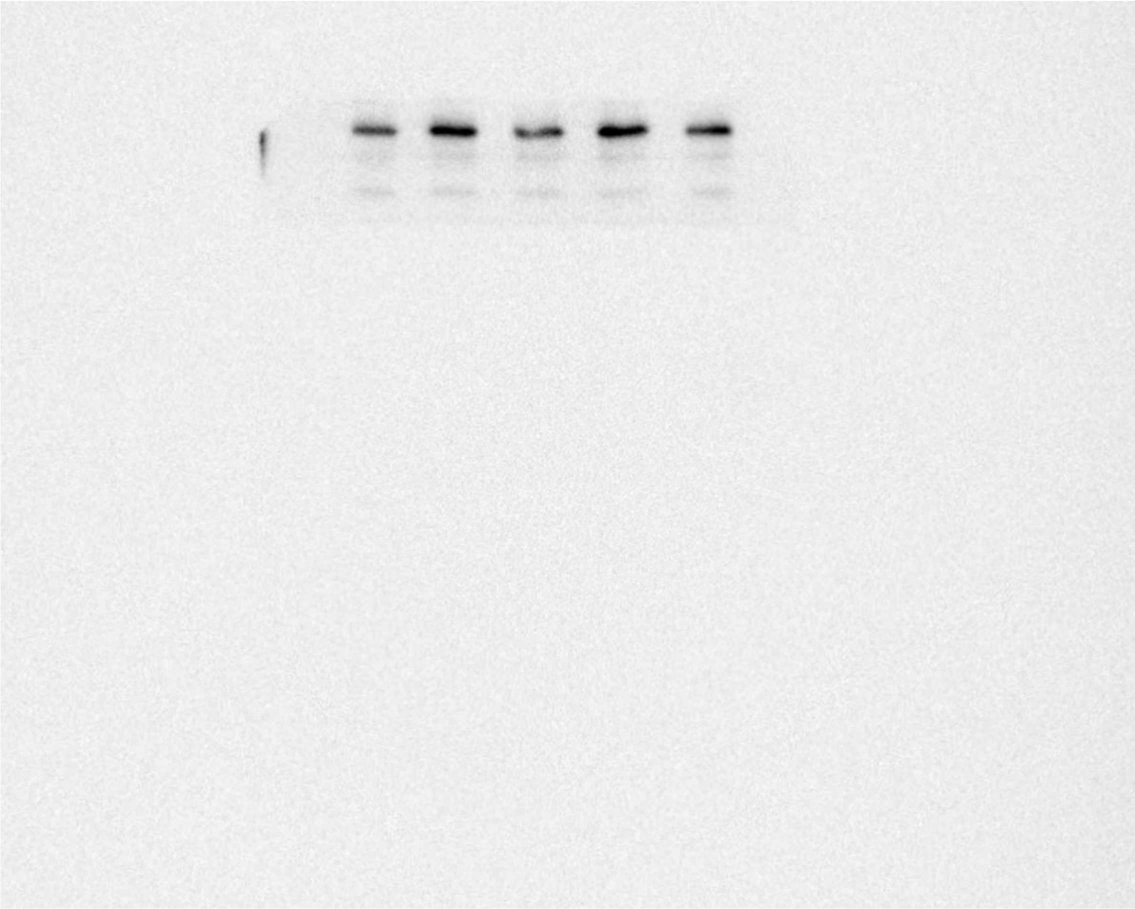

P62

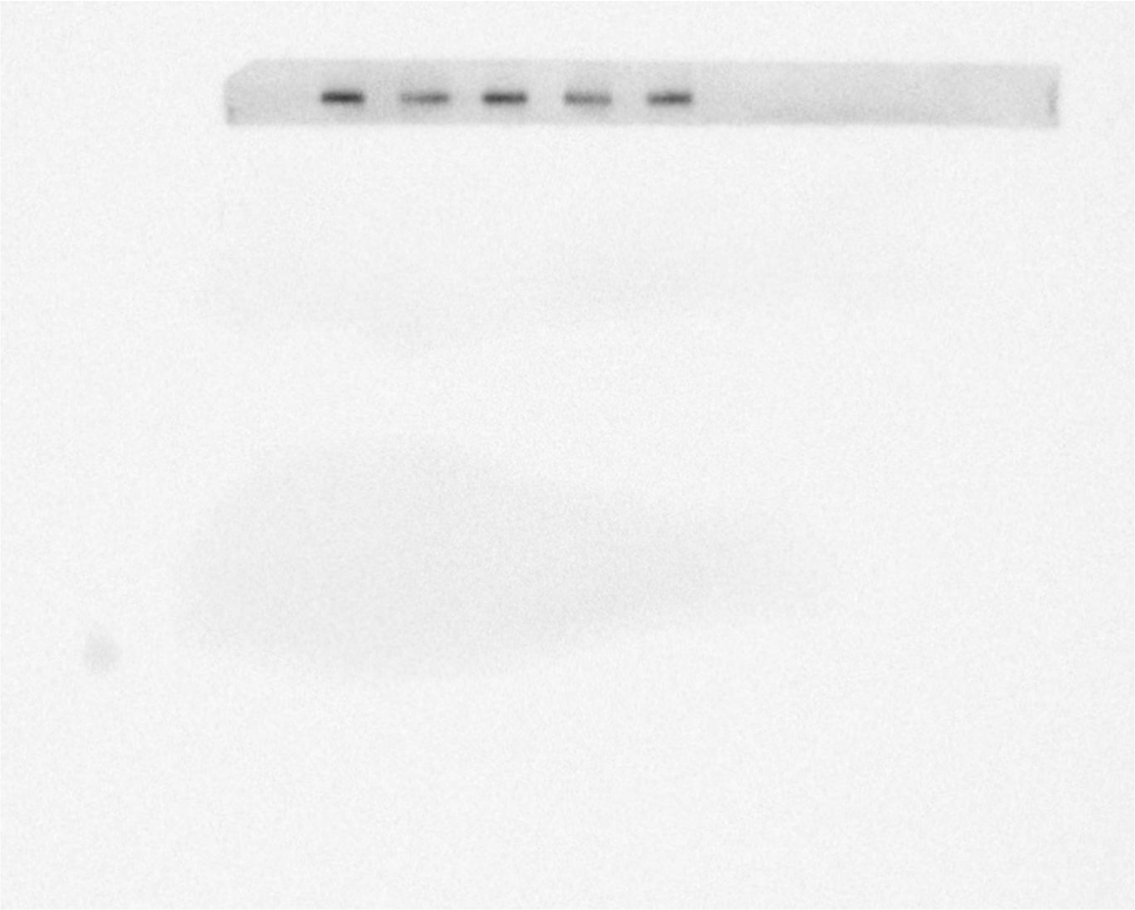

**Figure 1D MCF-7**

**β-actin**

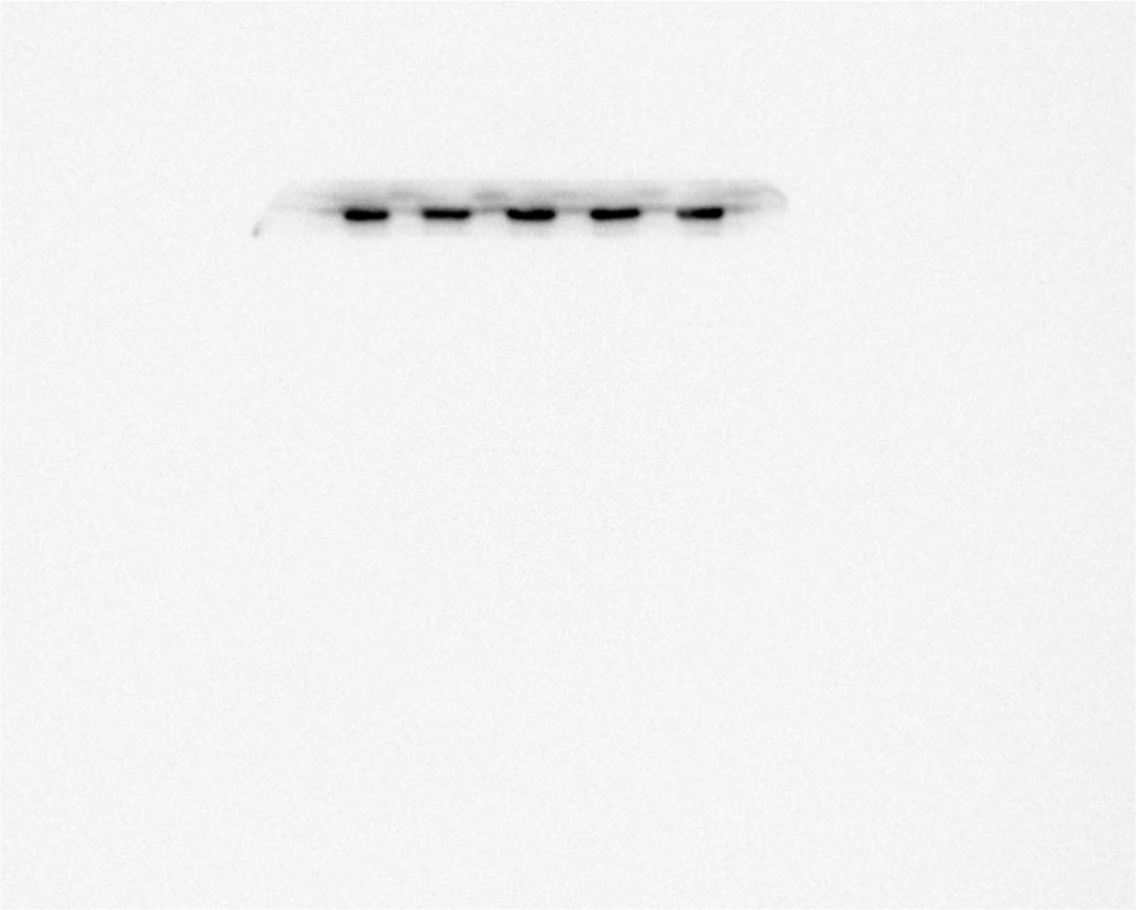

**LC3**

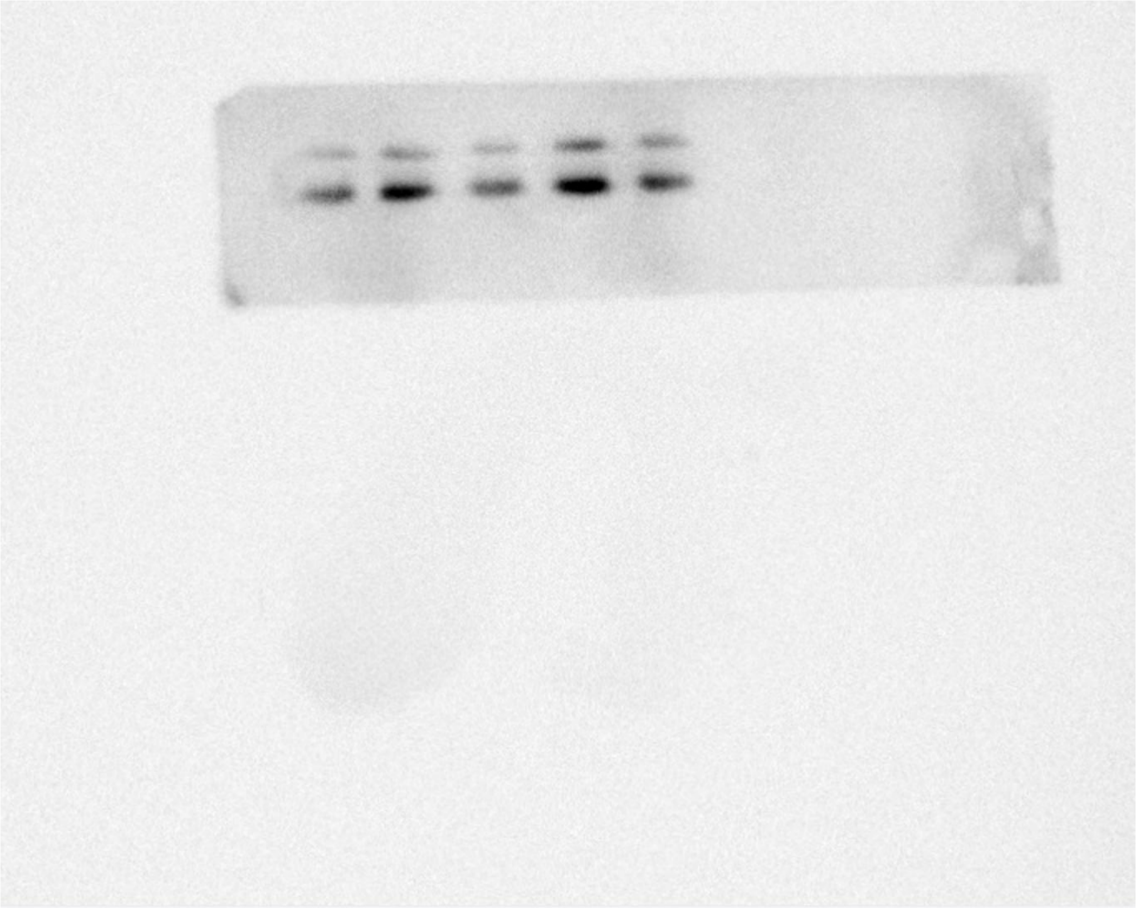

**Figure 2B MDA-MB-231**

ABCG2

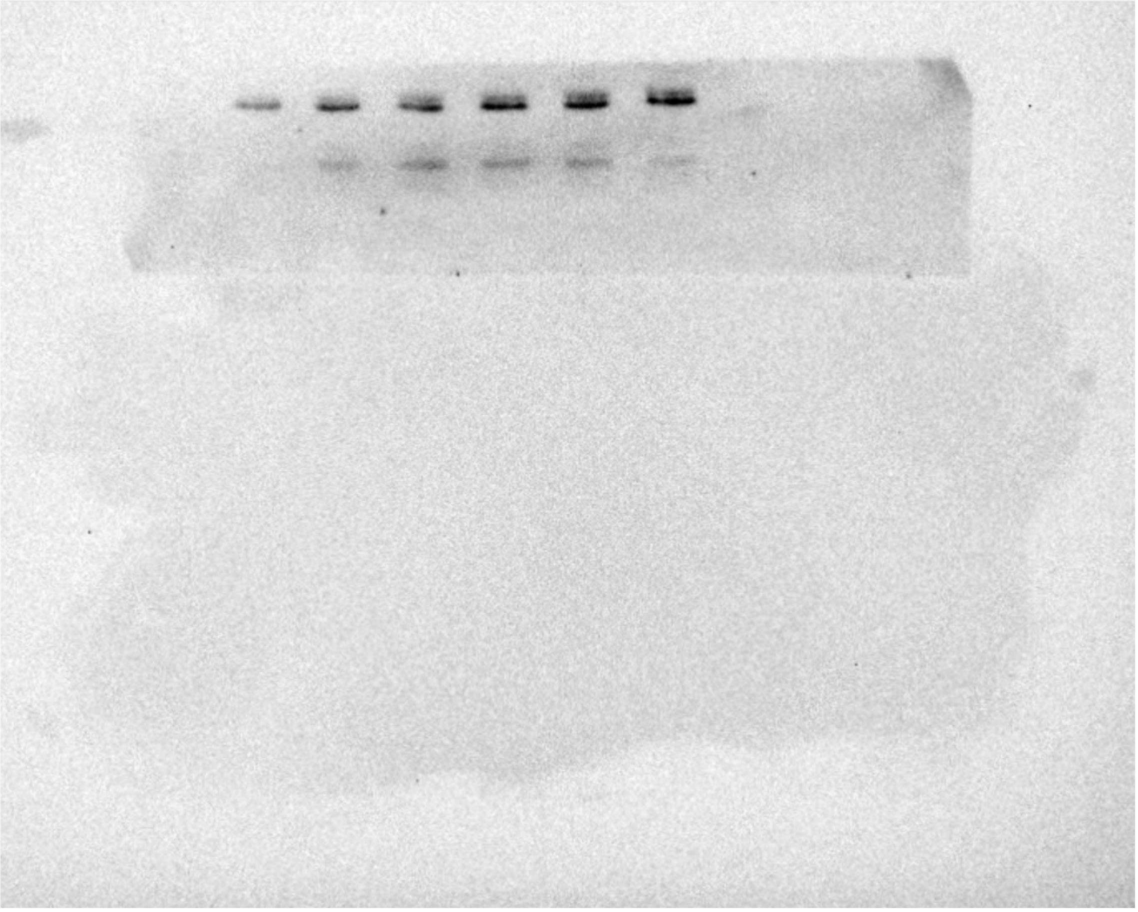

P62

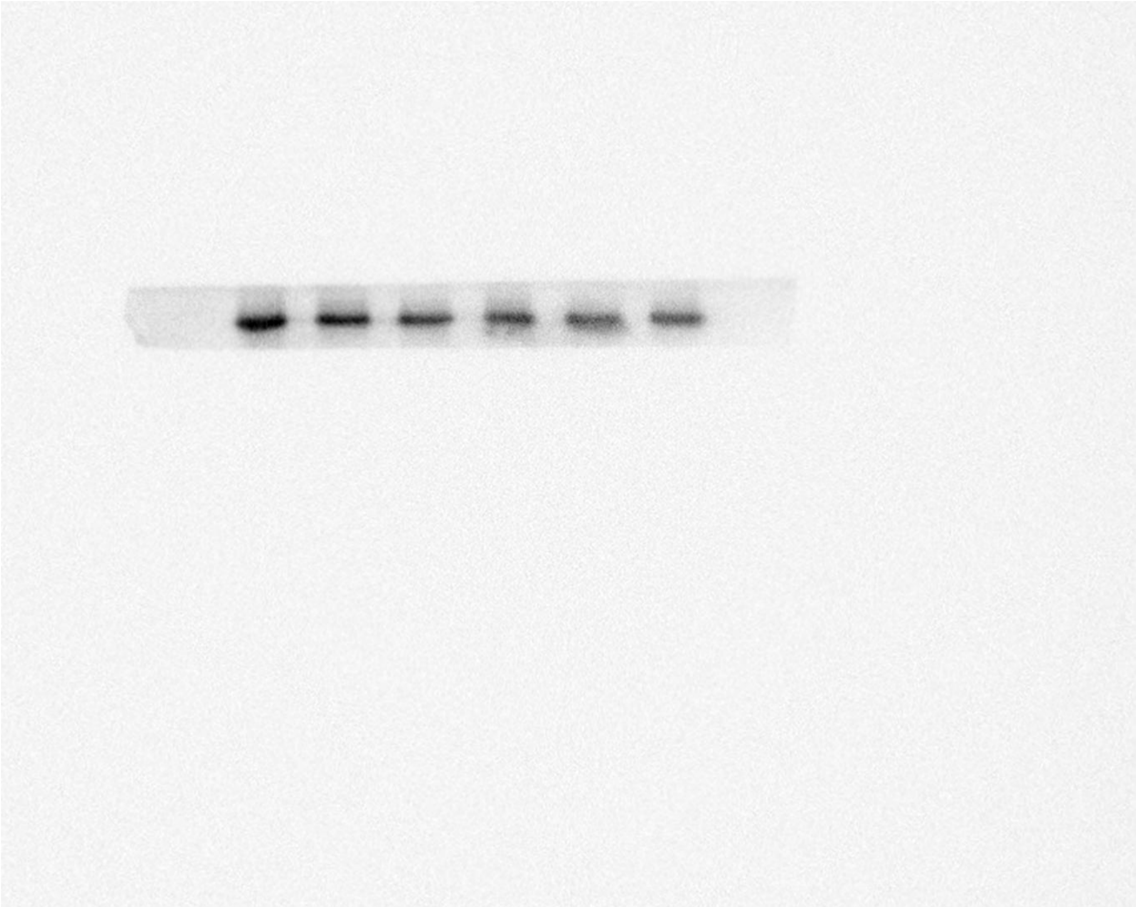

# Figure 2B MDA-MB-231

β-actin

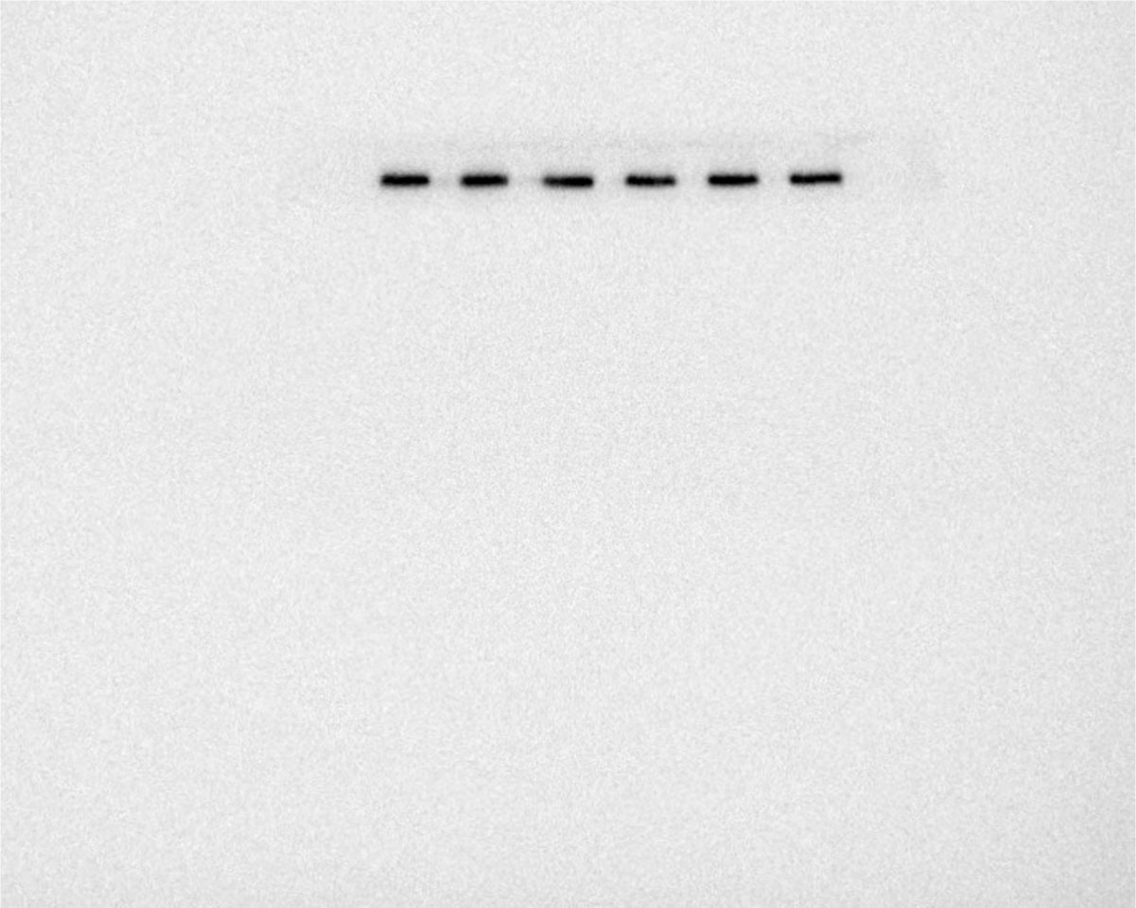

LC-3

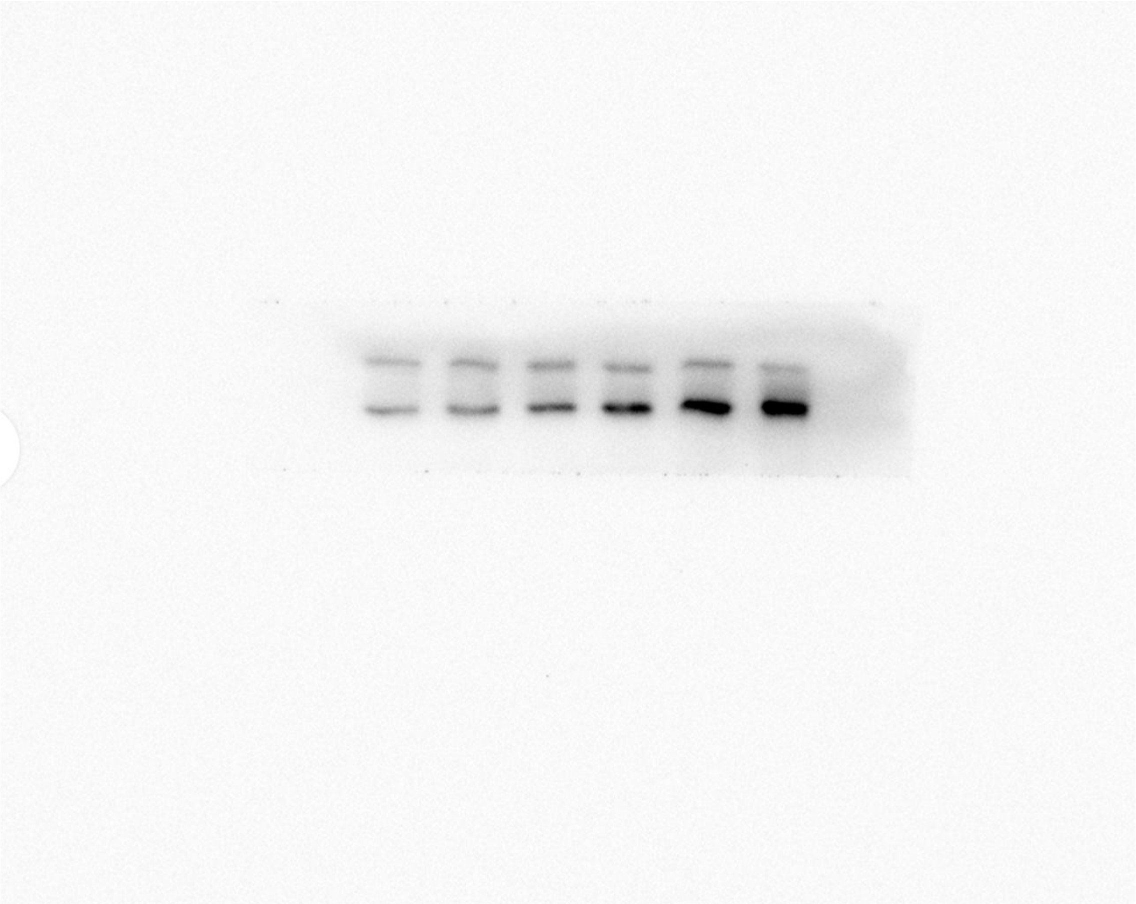

# Figure 2B MCF-7

**ABCG2**

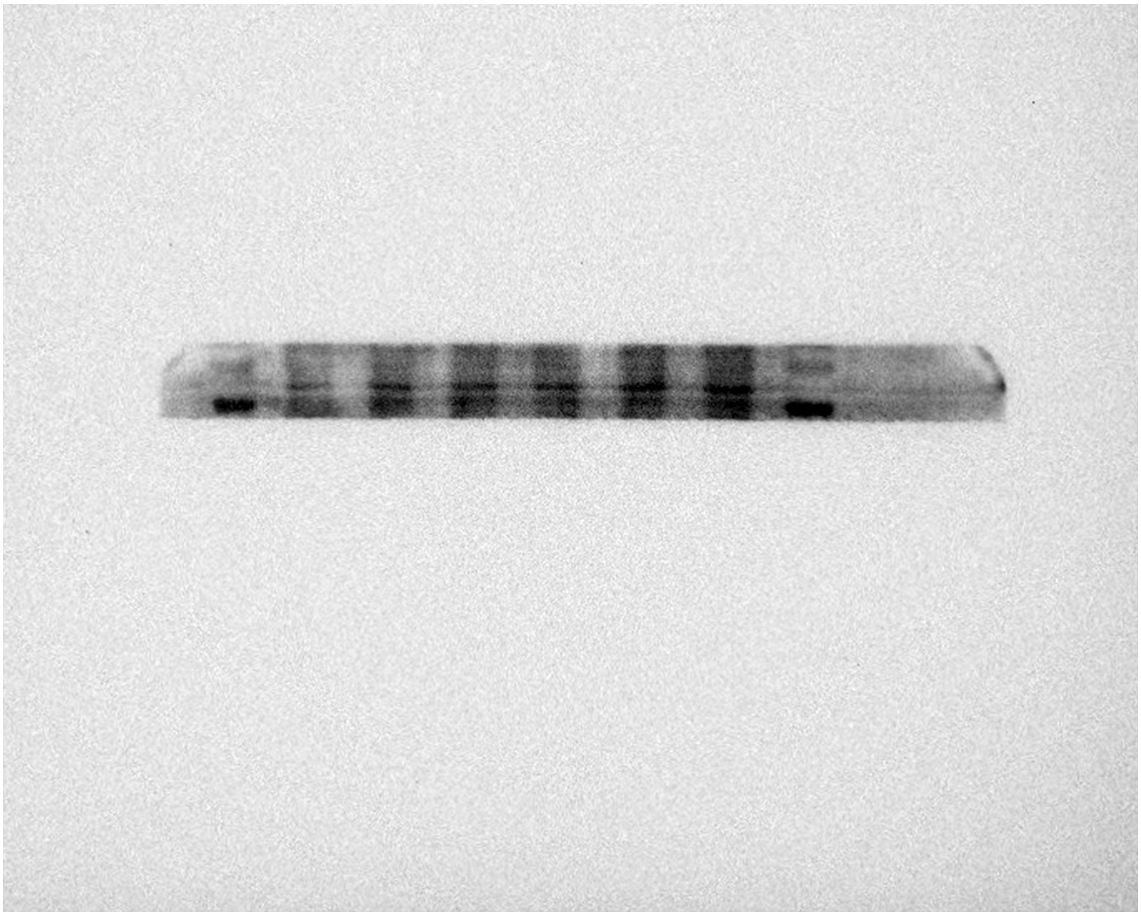

**P62**

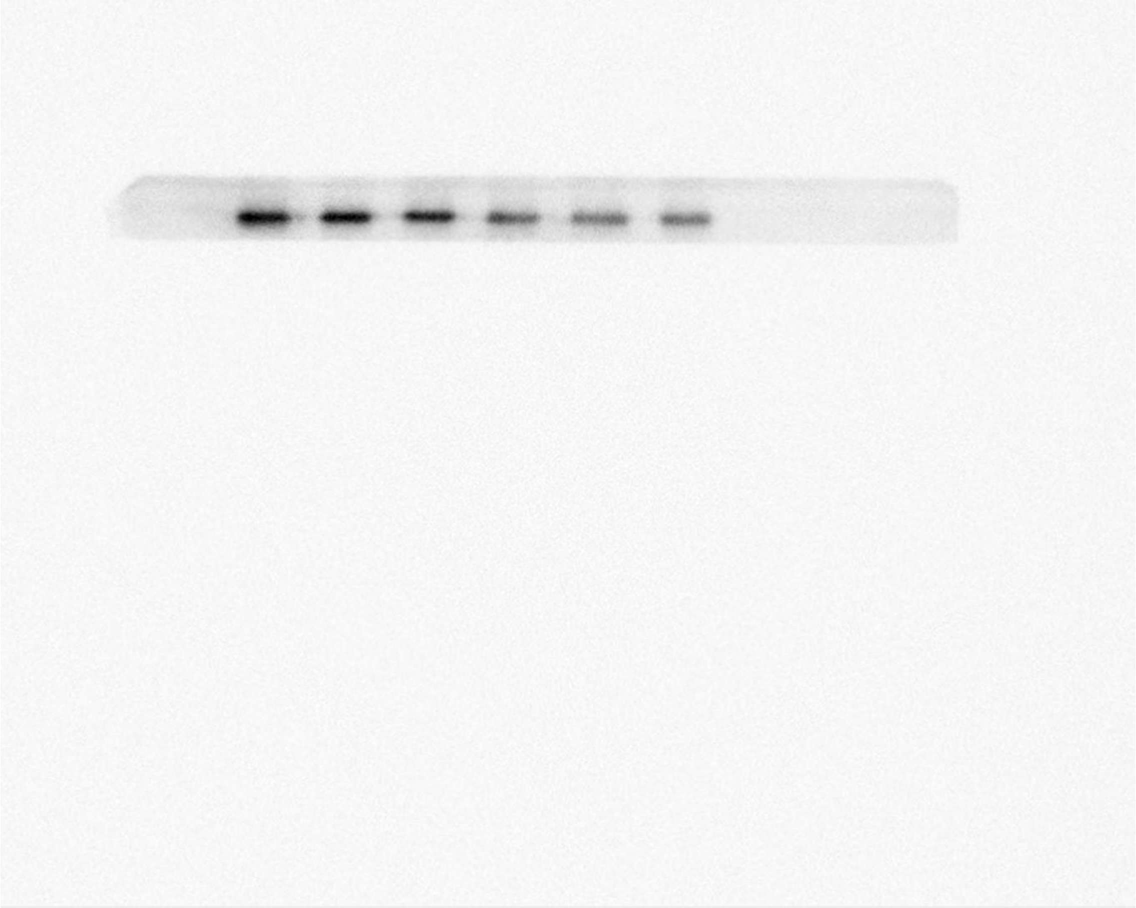

**Figure 2B MCF-7**

β-actin

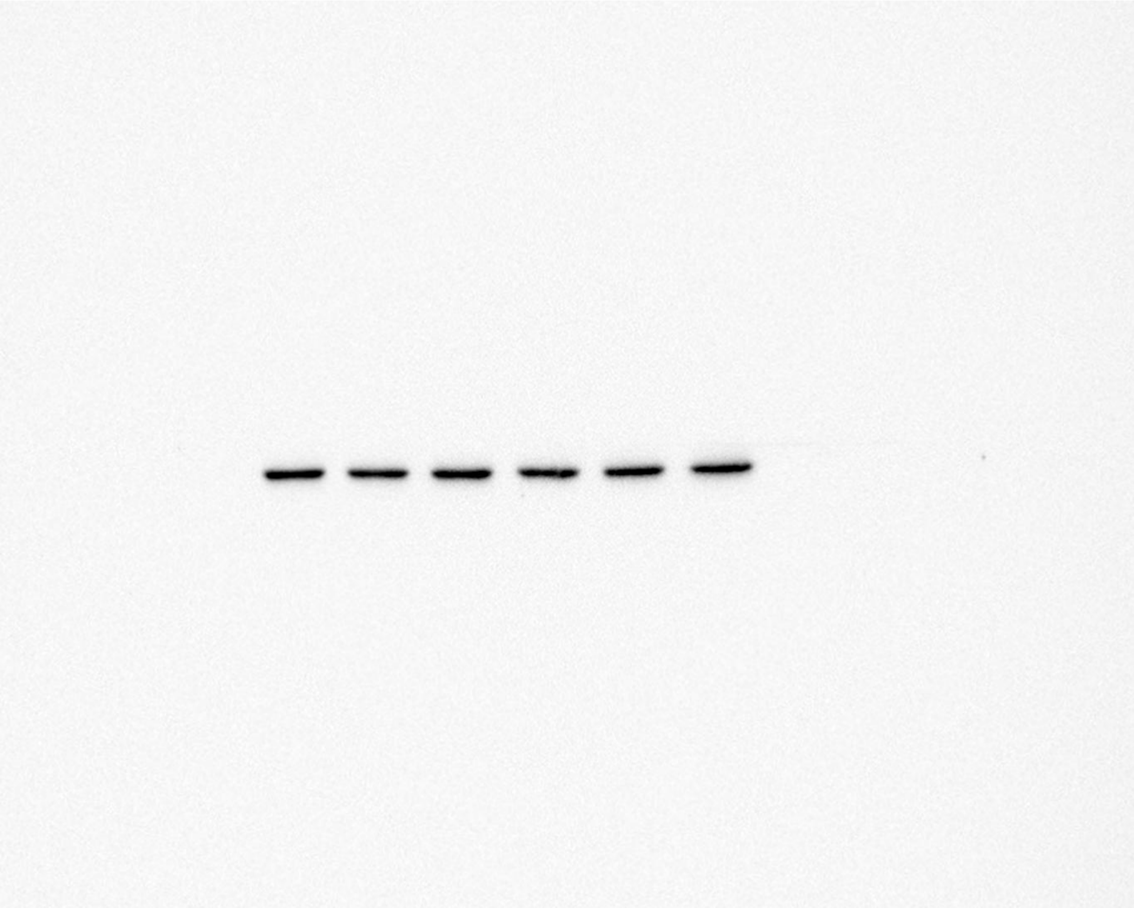

LC-3

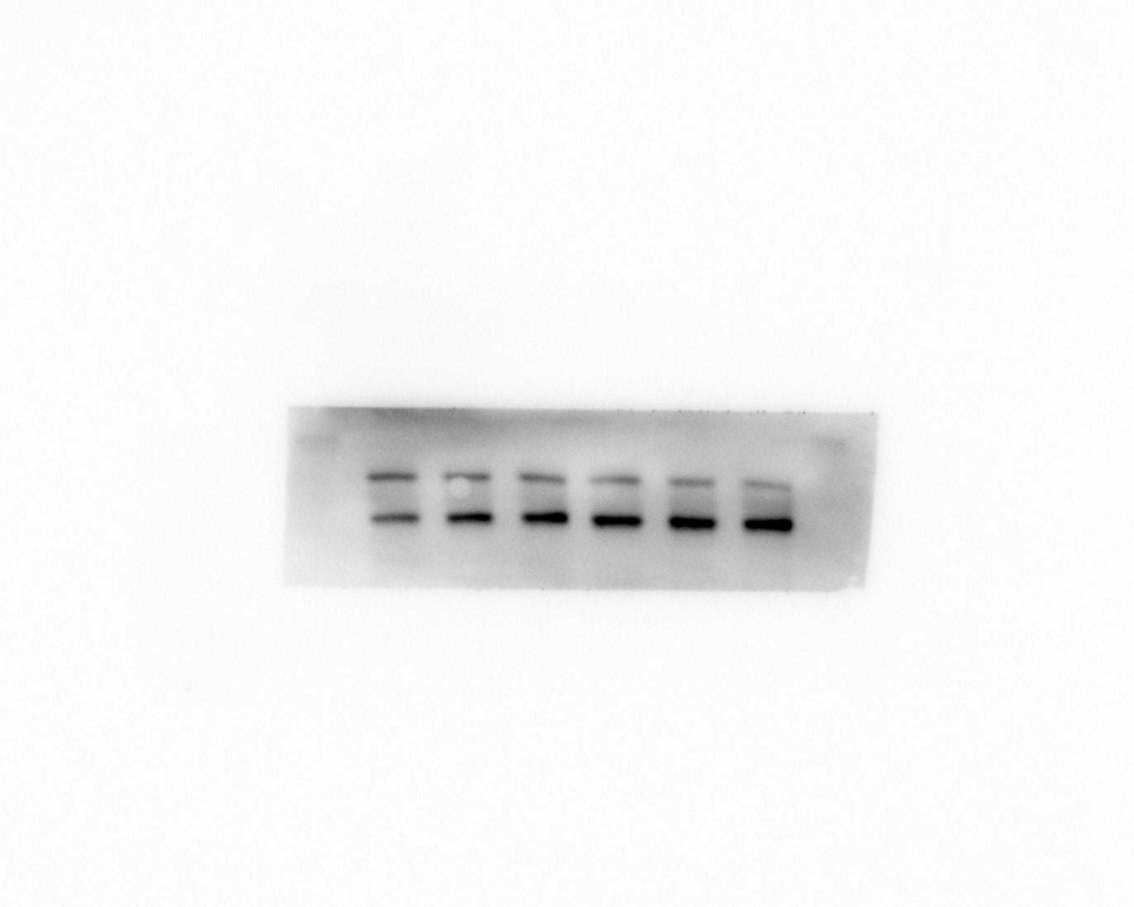

**Figure 2D MDA-MB-231**

**ABCG2**

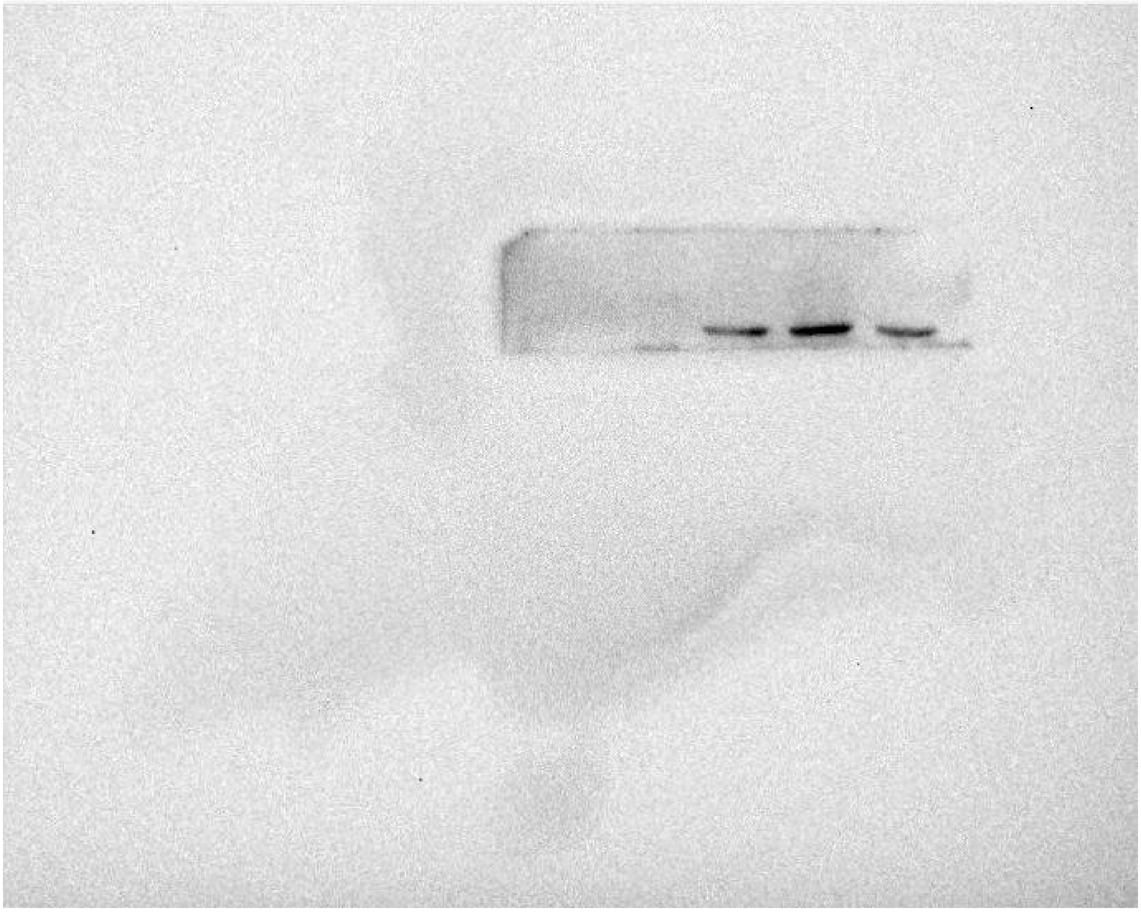

**P62**

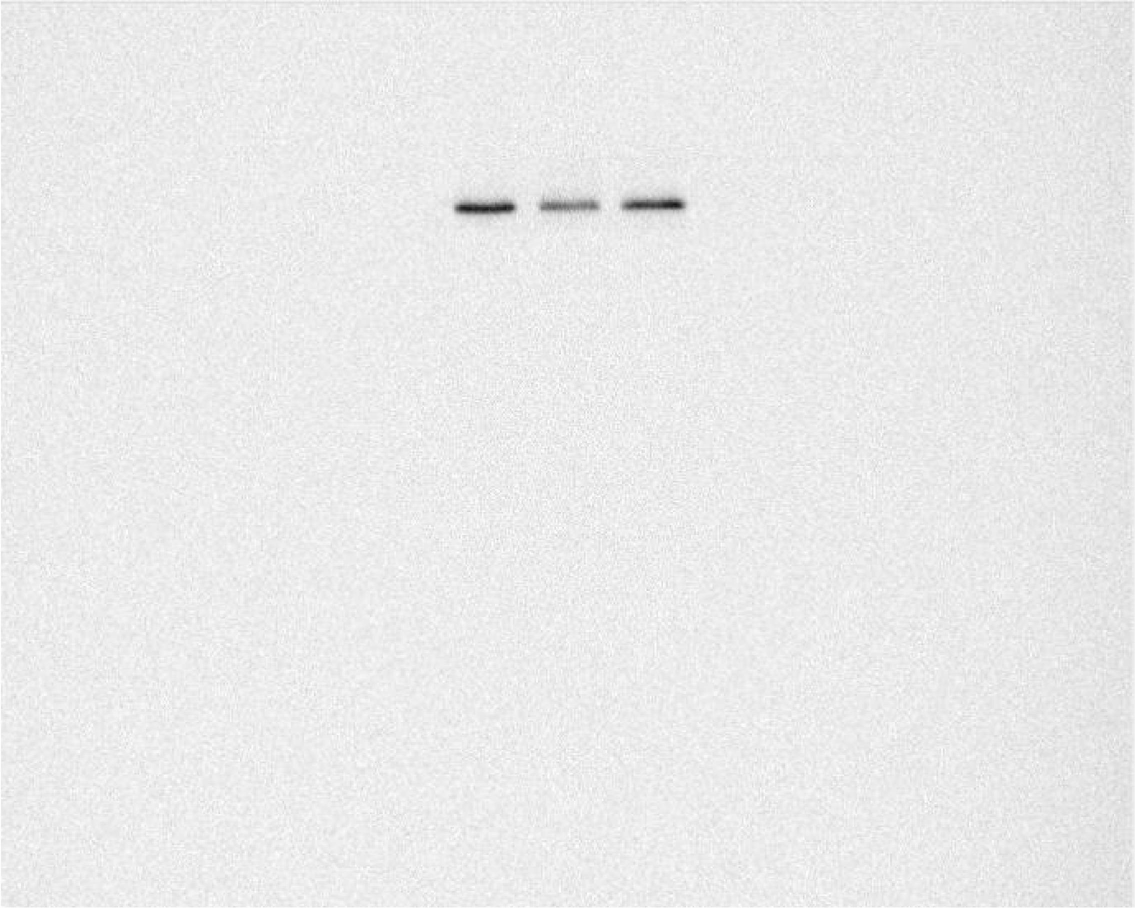

Figure 2D MDA-MB-231

β-actin

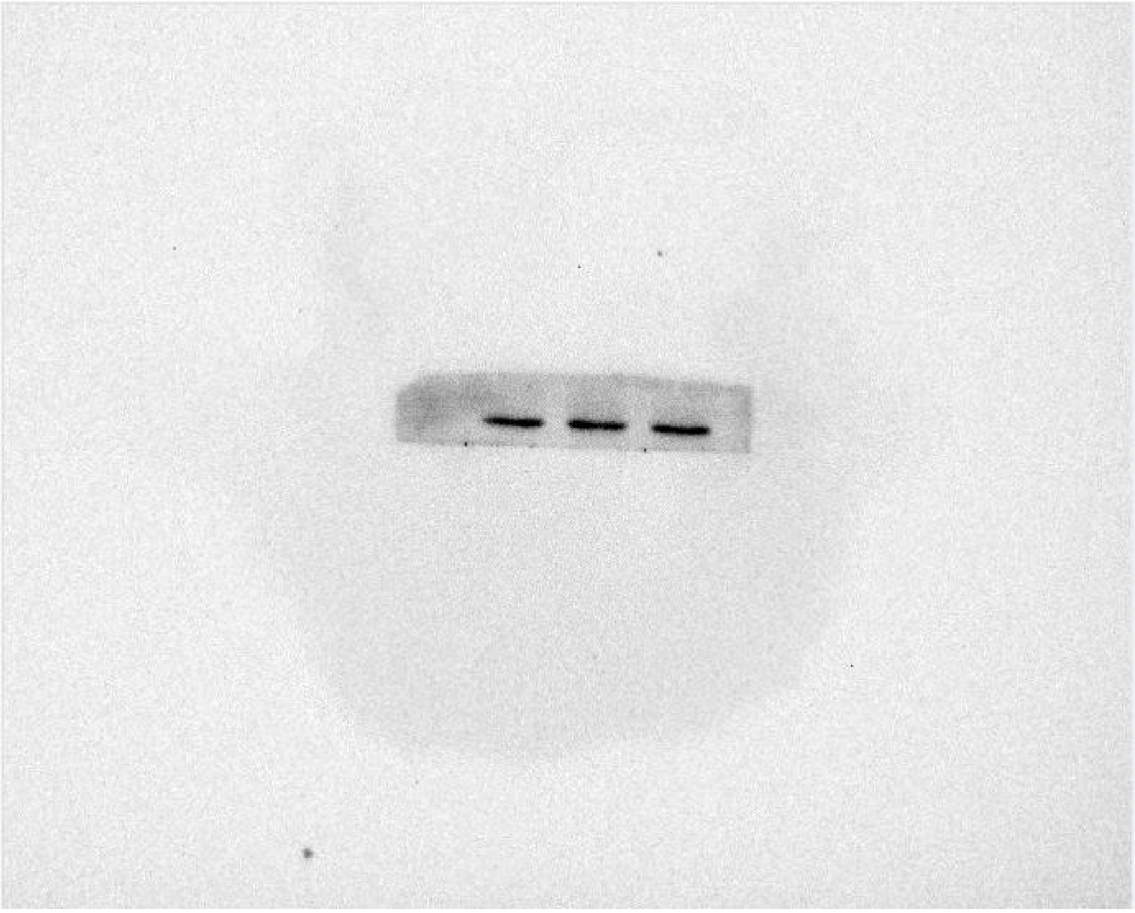

LC-3

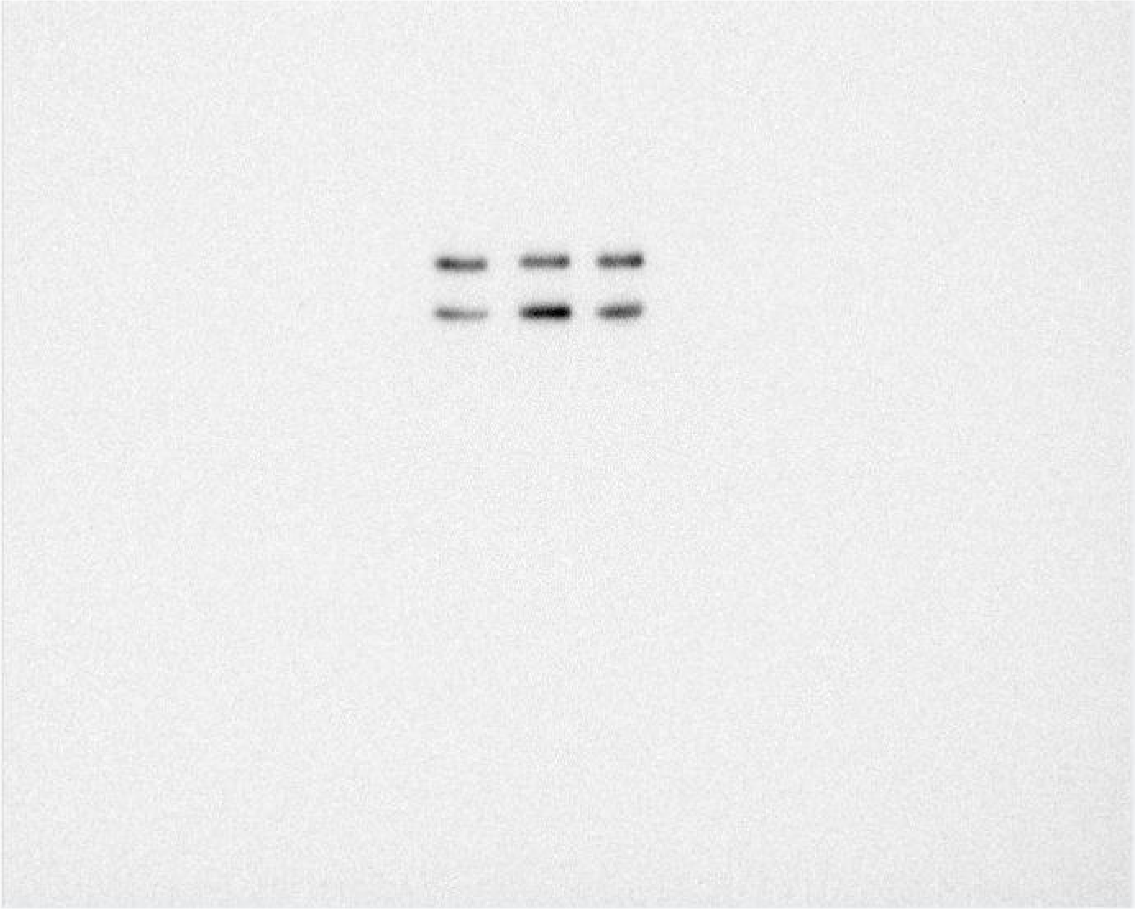

# Figure 2D MCF-7

ABCG2

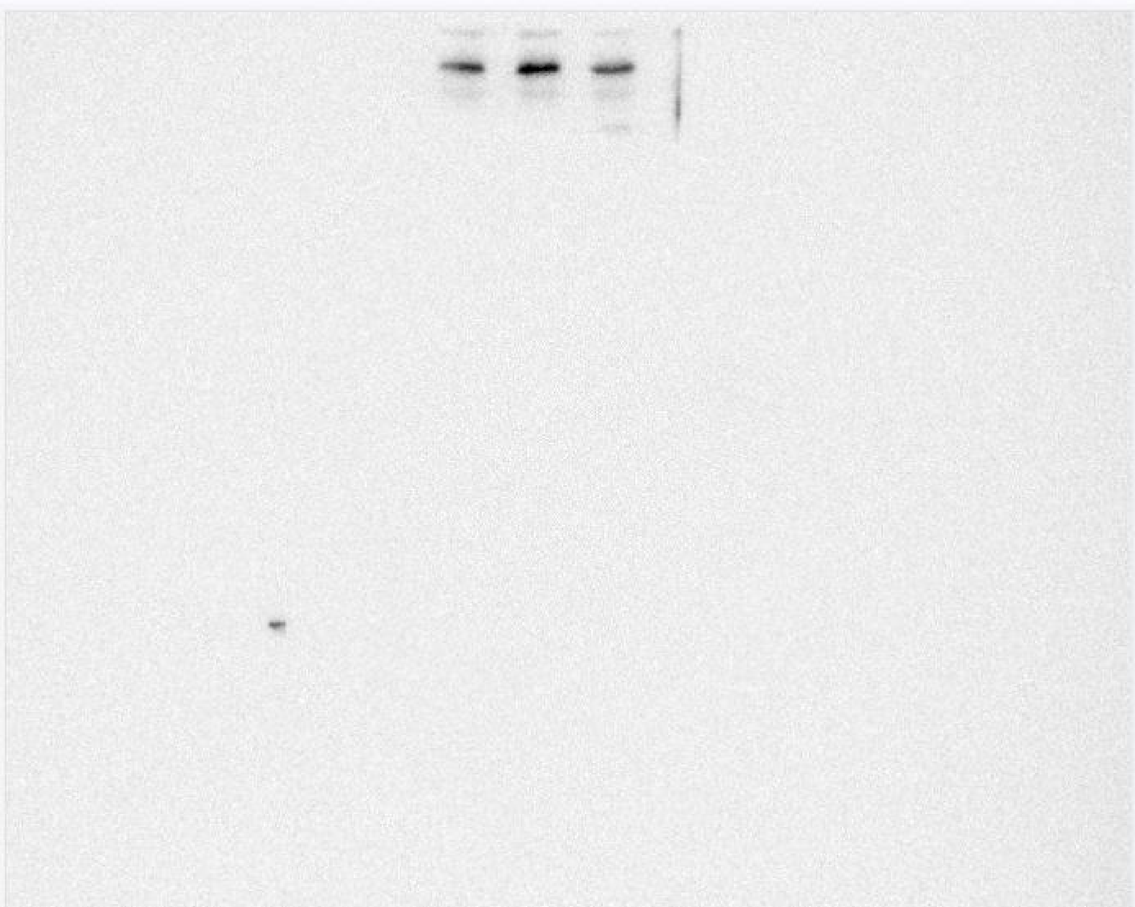

P62

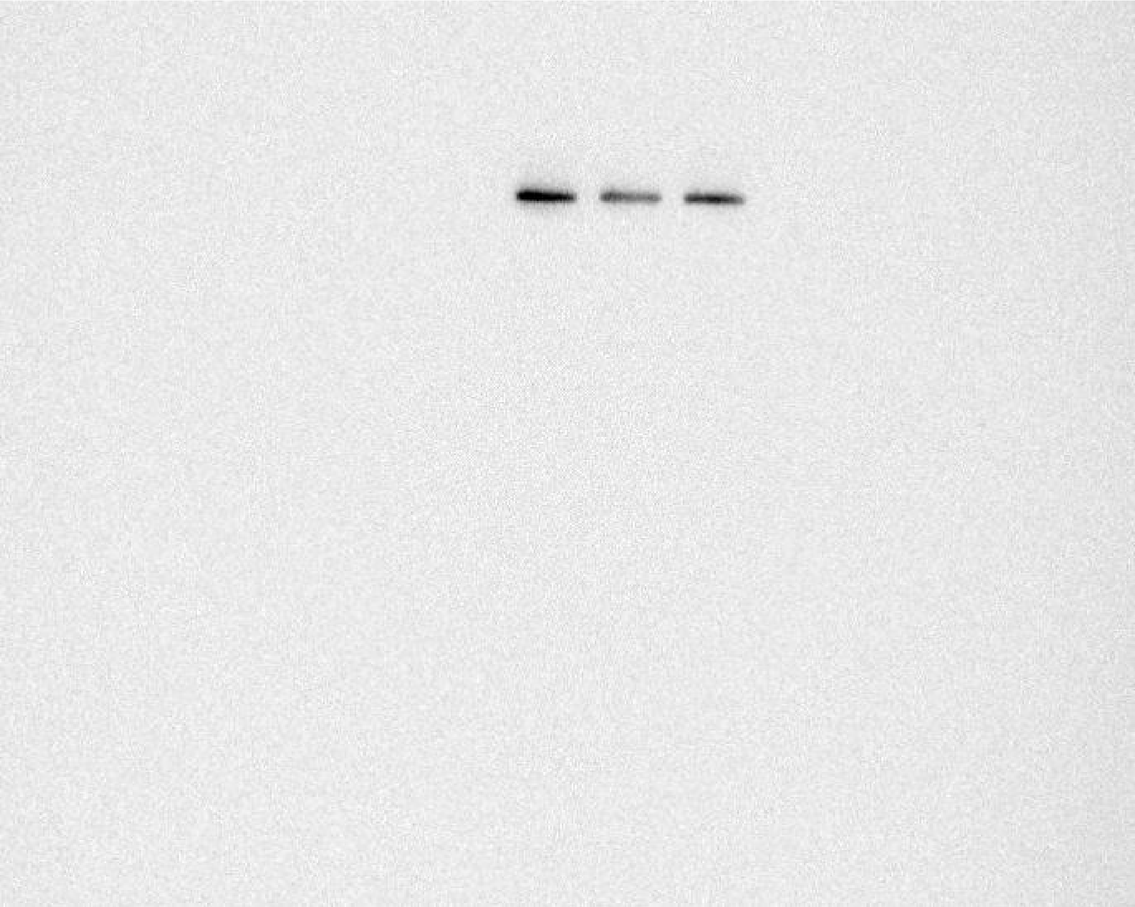

**Figure 2D MCF-7**

β-actin

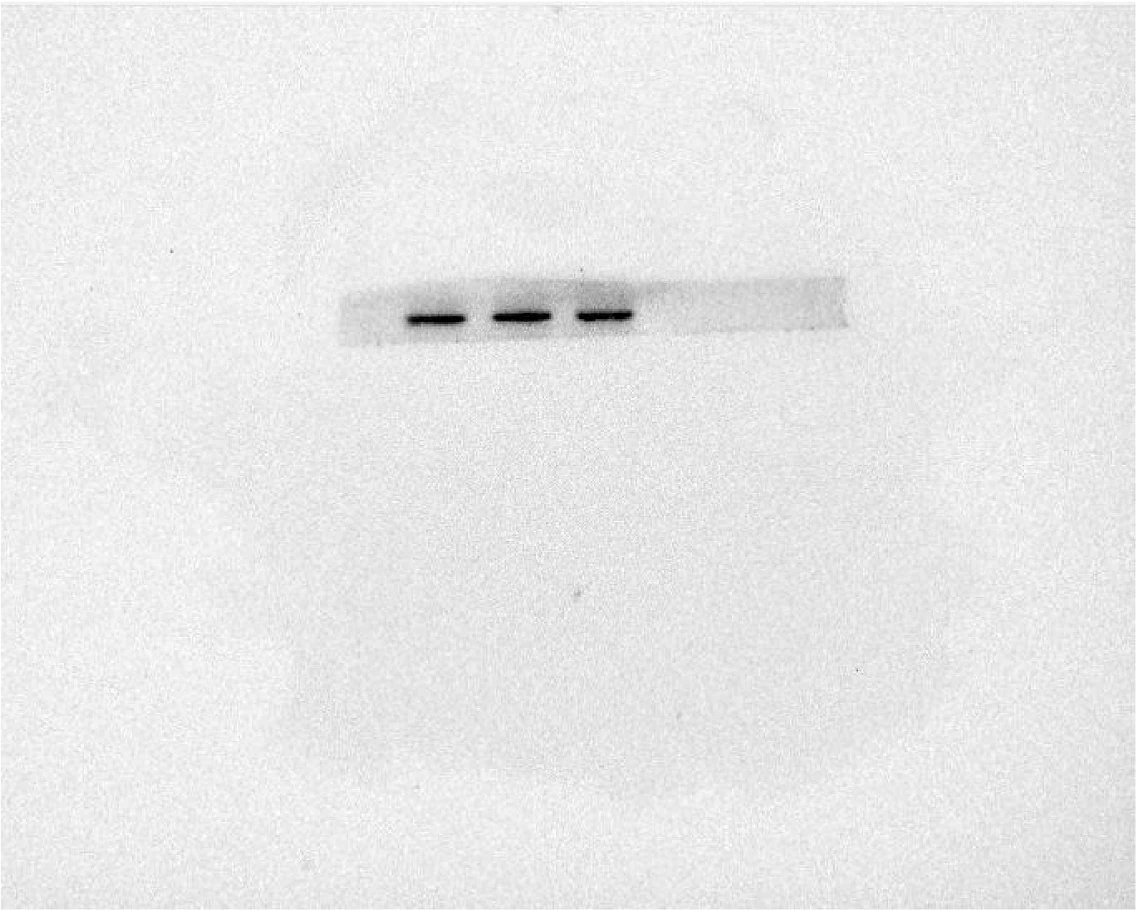

LC-3

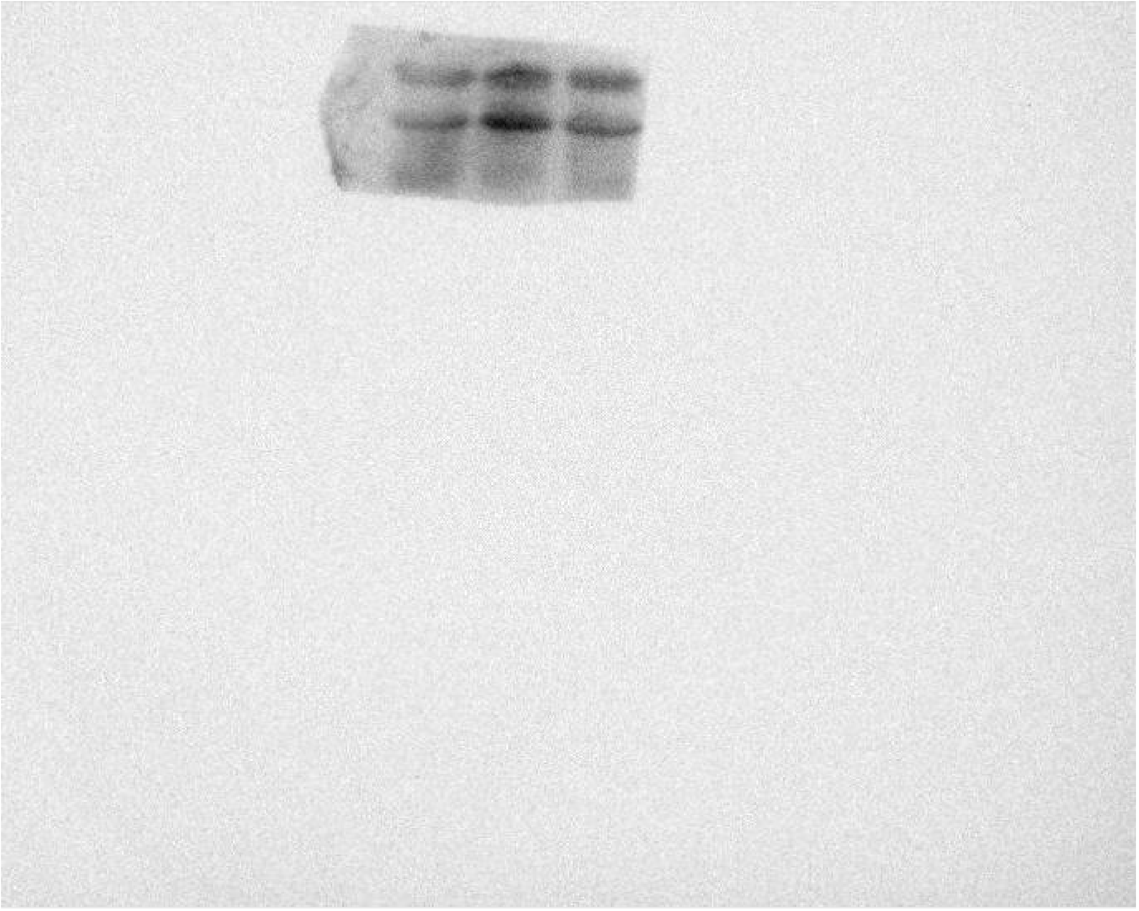

**Figure 3A *CXCL1*$^{OE}$**

ABCG2

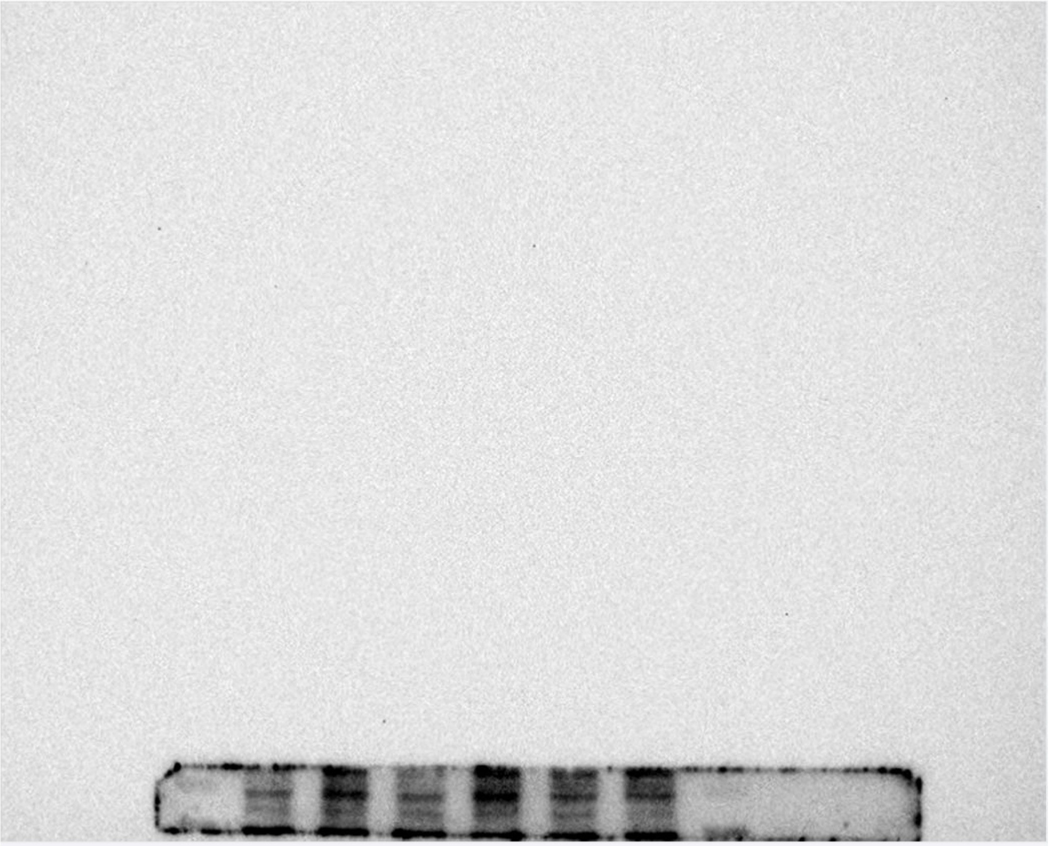

P62

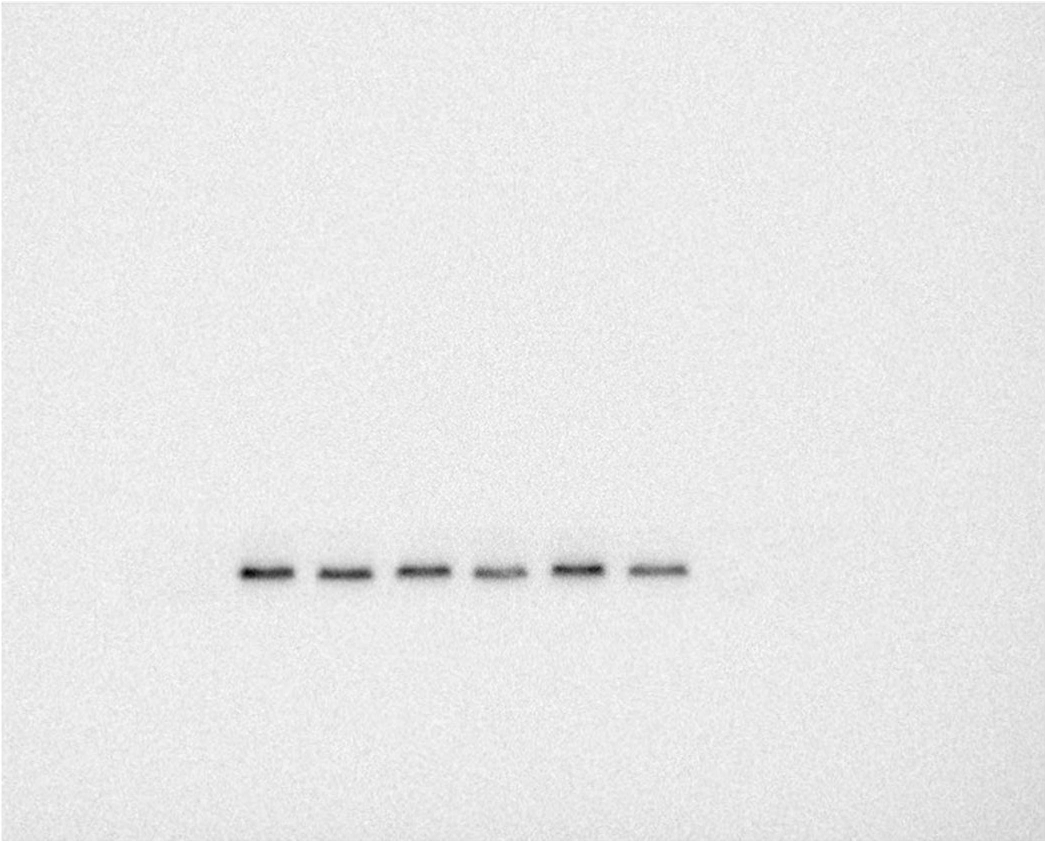

**Figure 3A** ***CXCL1^OE^***

**β-actin**

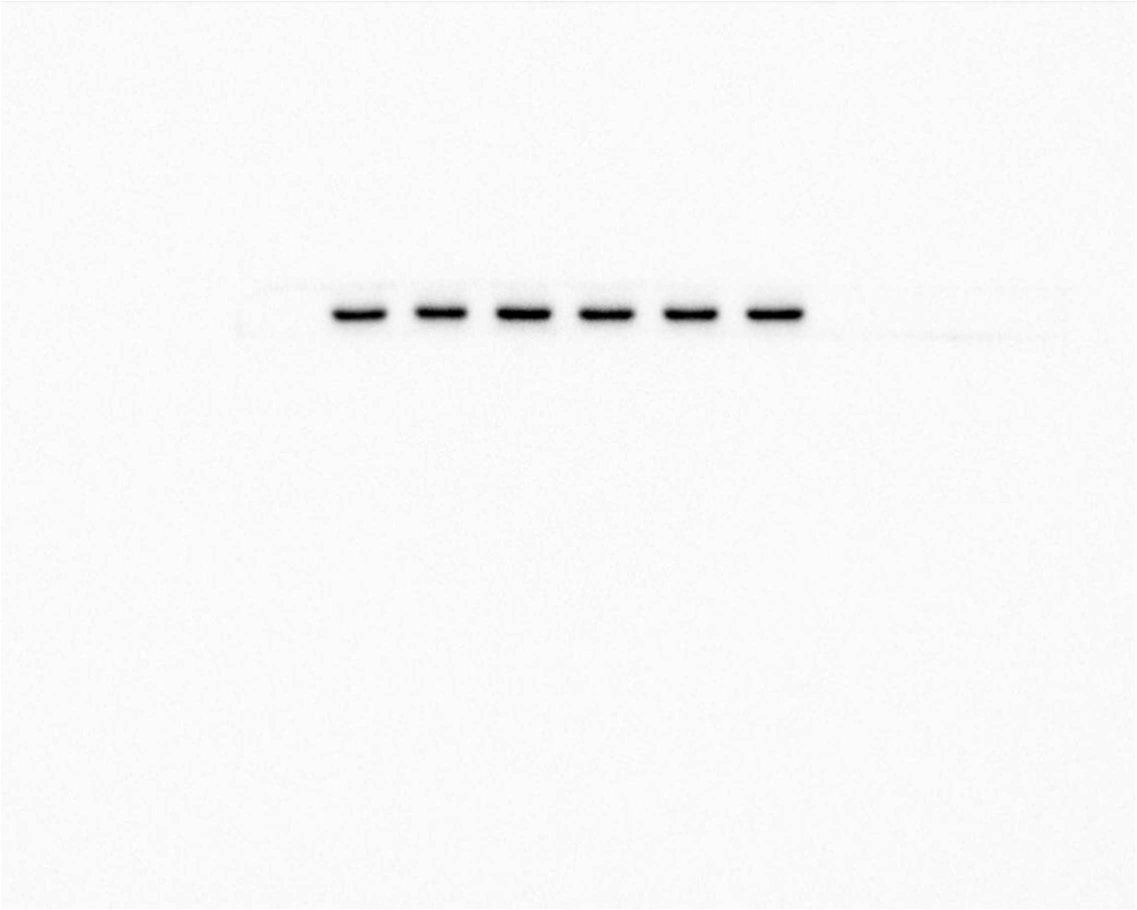

**LC3**

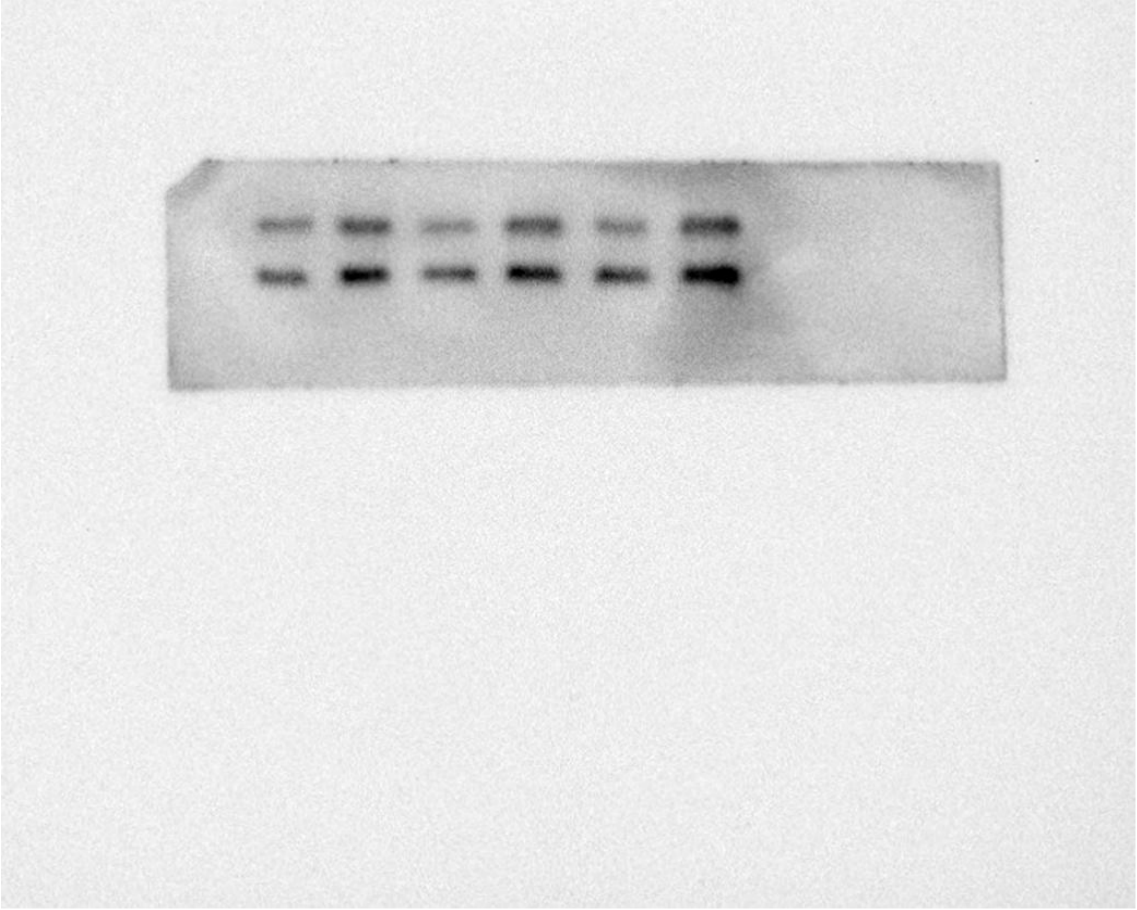

Figure 3A *CXCL1$^{OE}$*

CXCL1

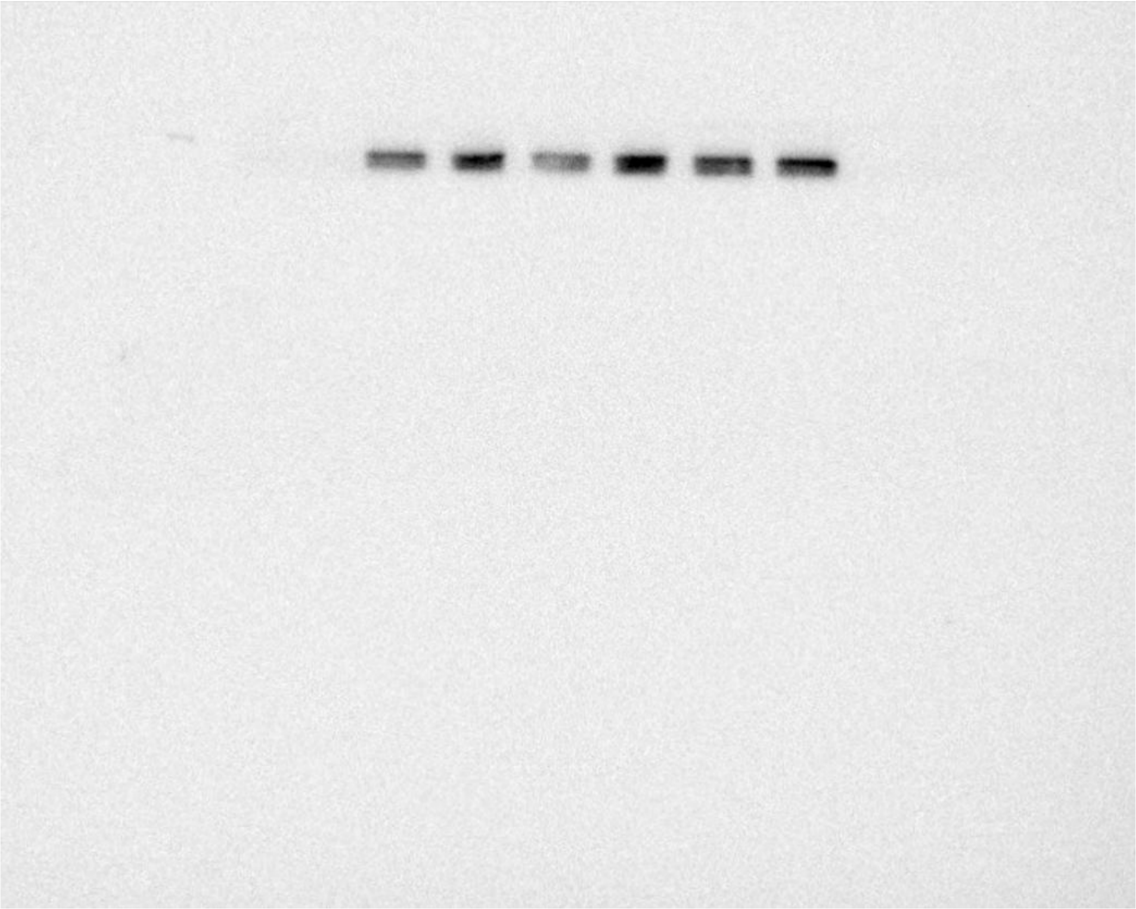

**Figure 3A *CXCL1*$^{KO}$**

**ABCG2**

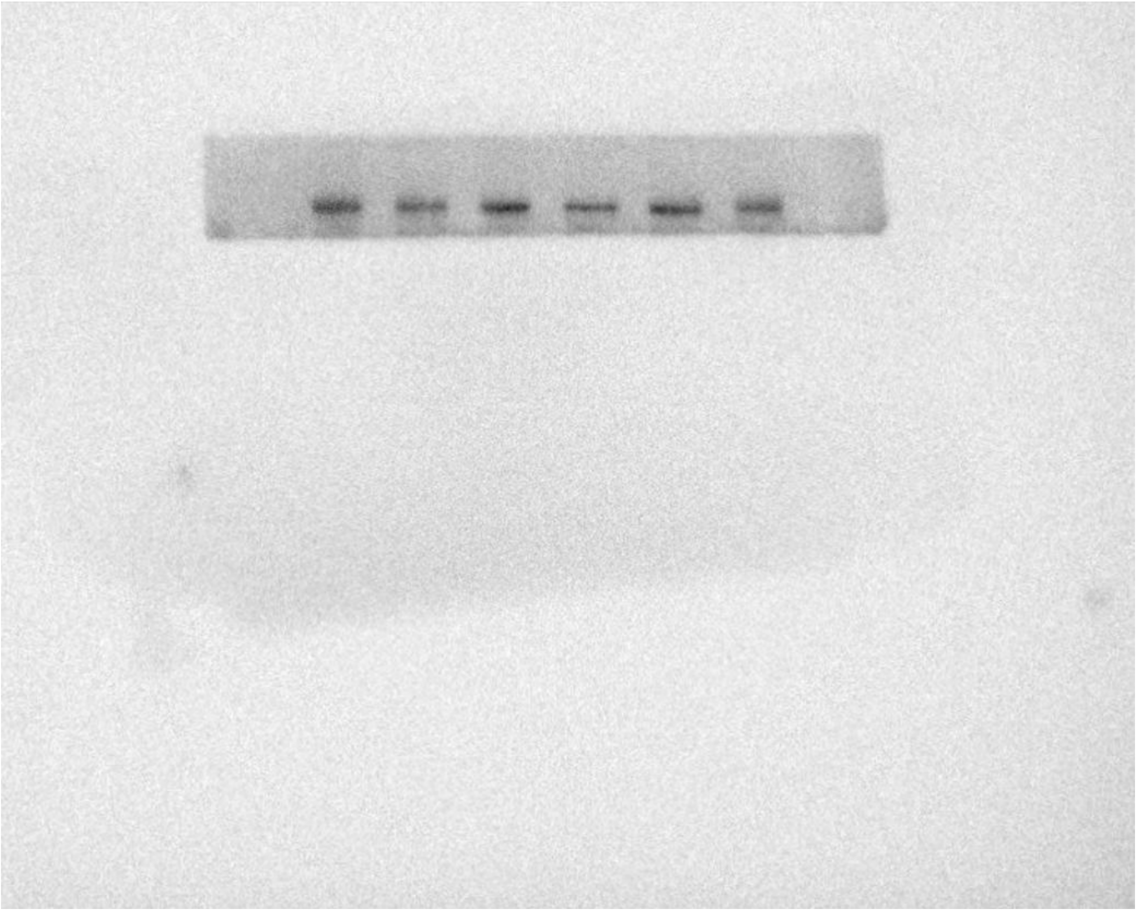

**P62**

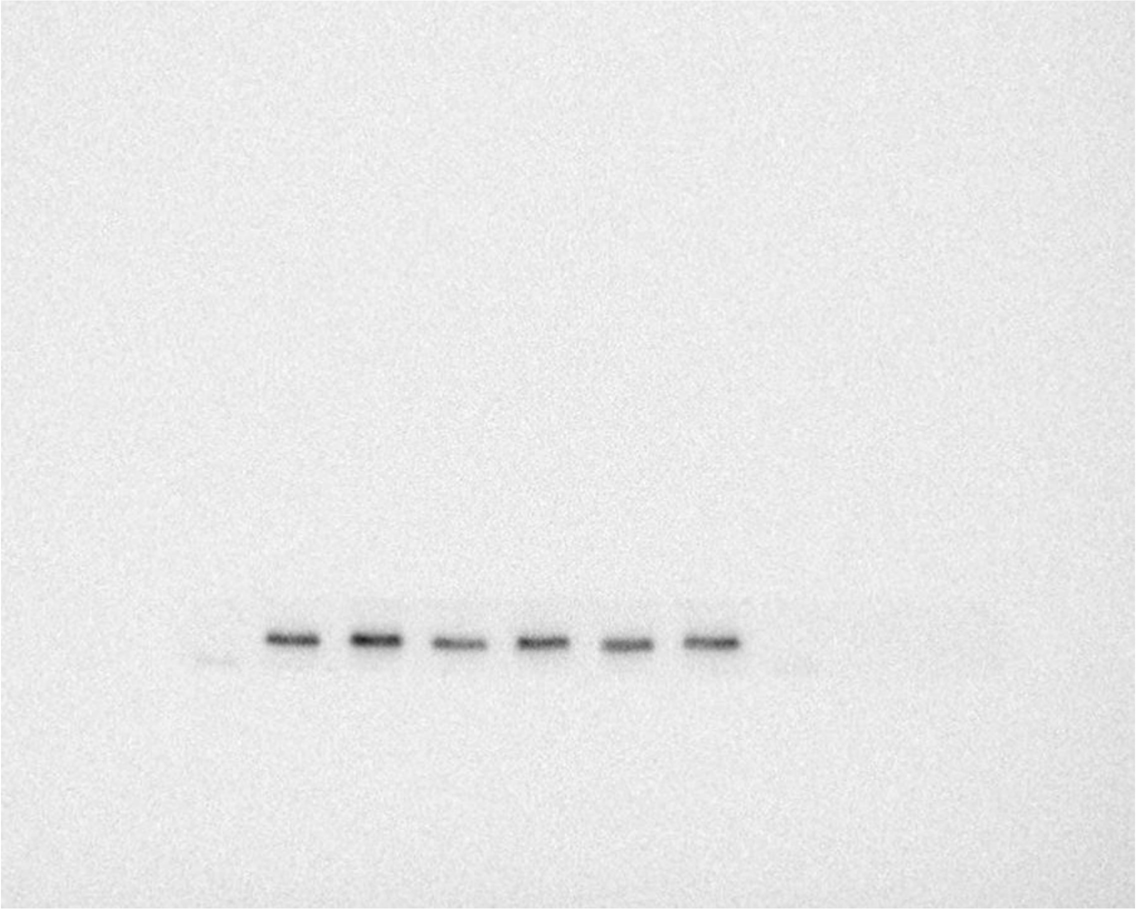

**Figure 3A *CXCL1*$^{KO}$**

**β-actin**

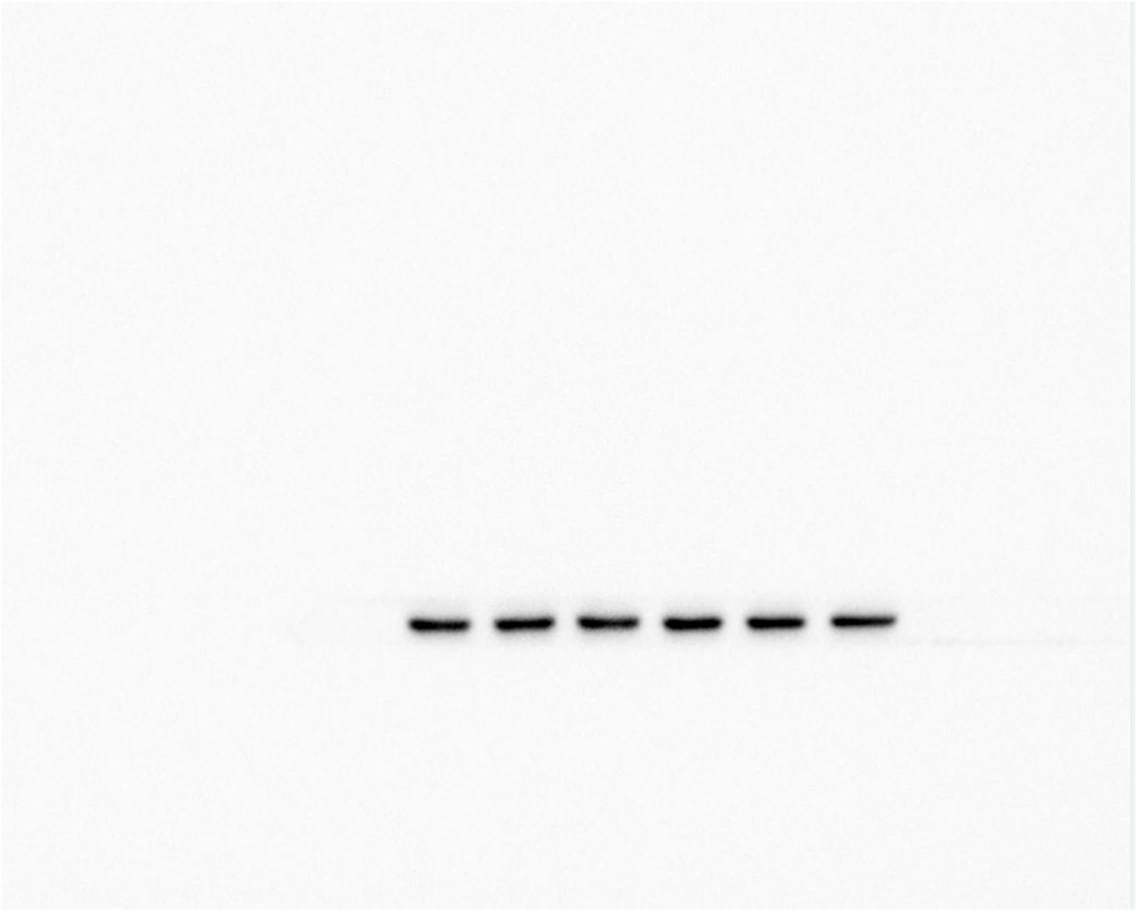

**LC3**

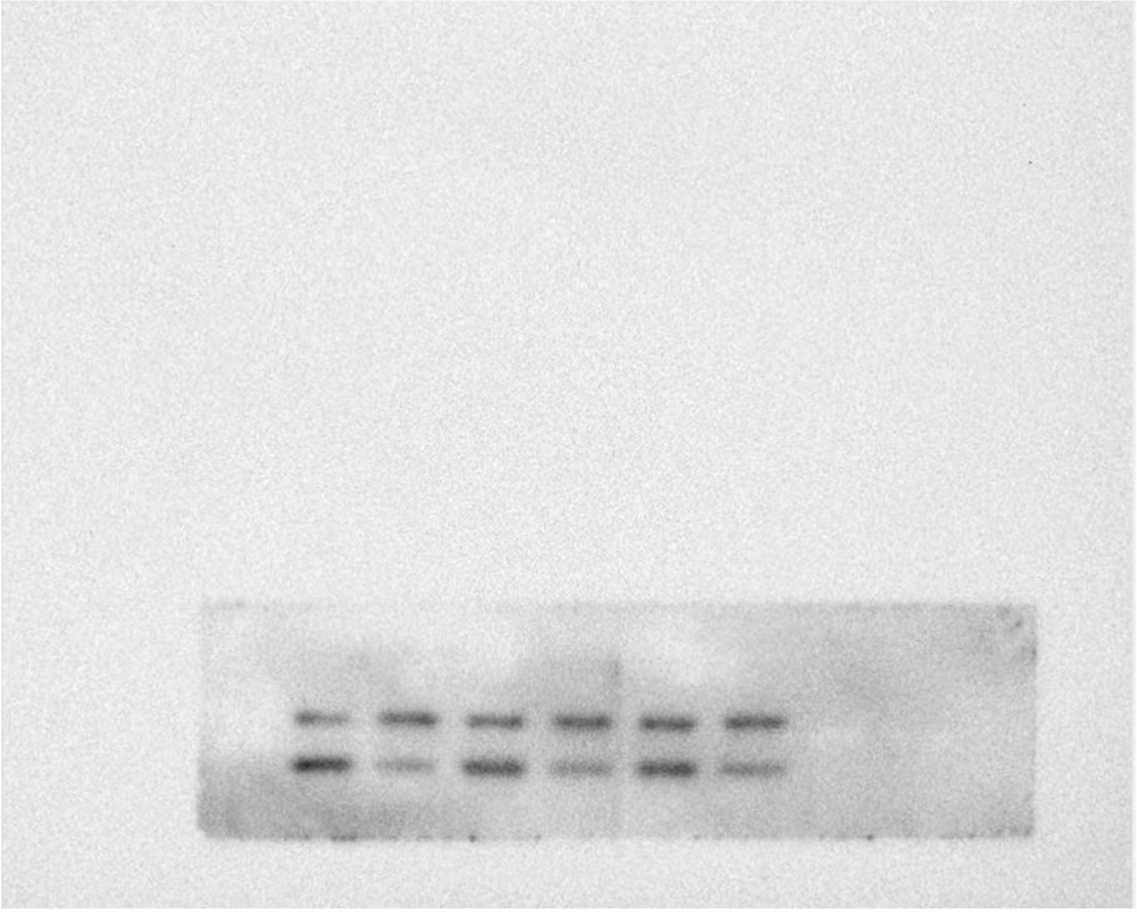

Figure 3A *CXCL1*$^{KO}$

CXCL1

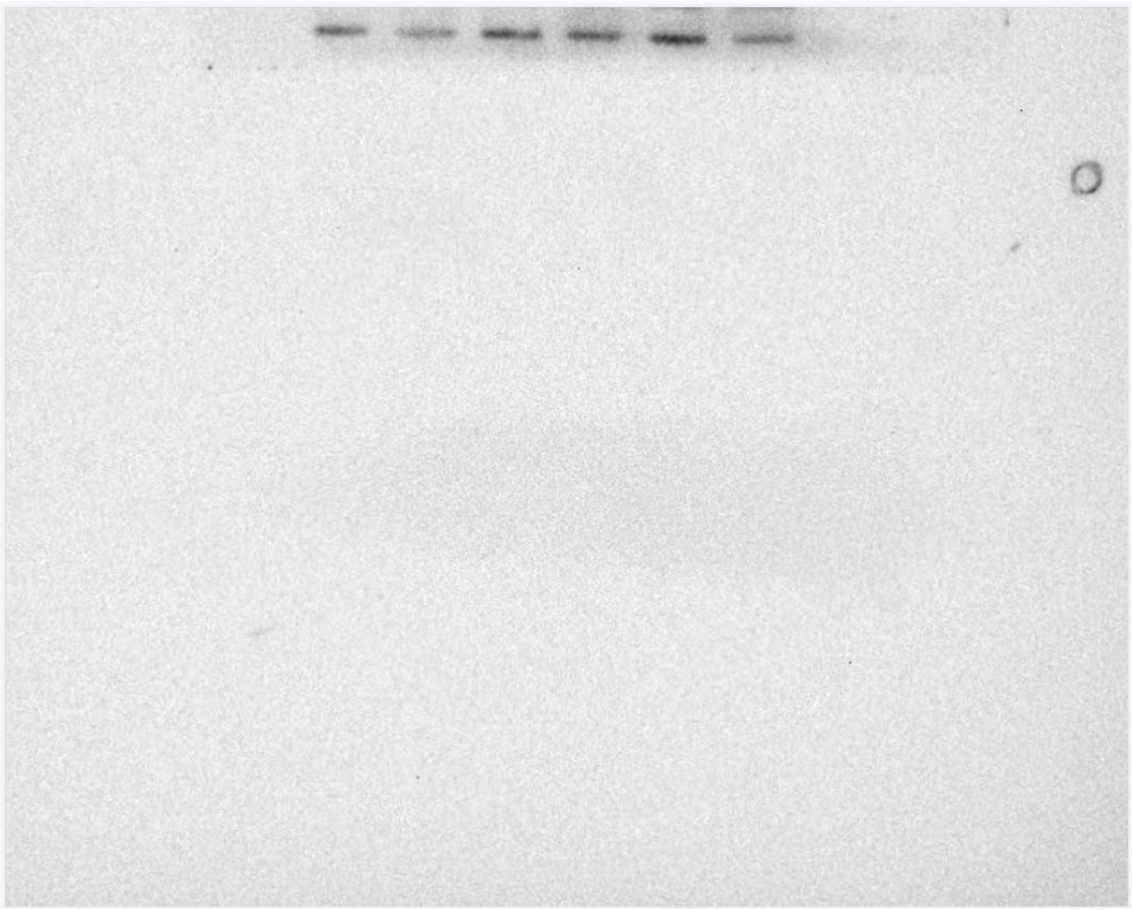

**Figure 3D *CXCL1$^{OE}$***

ABCG2

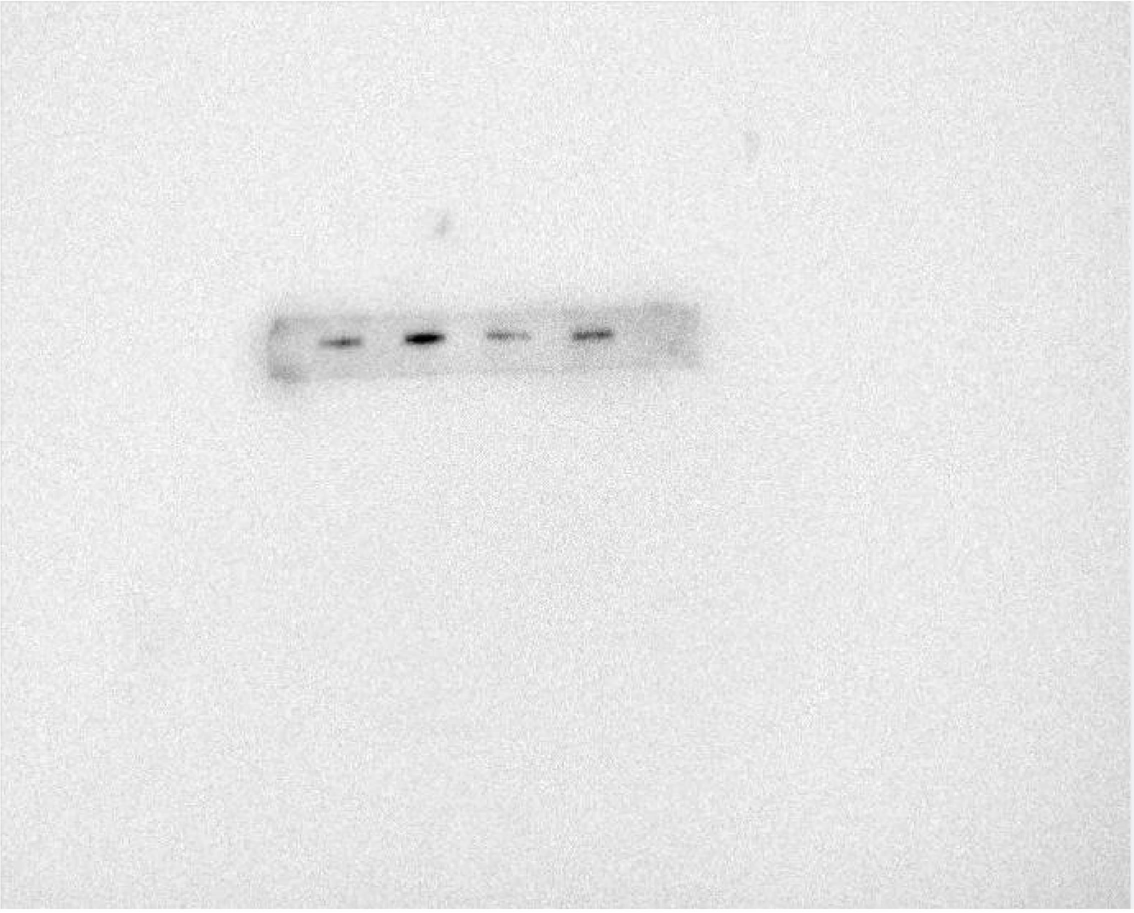

P62

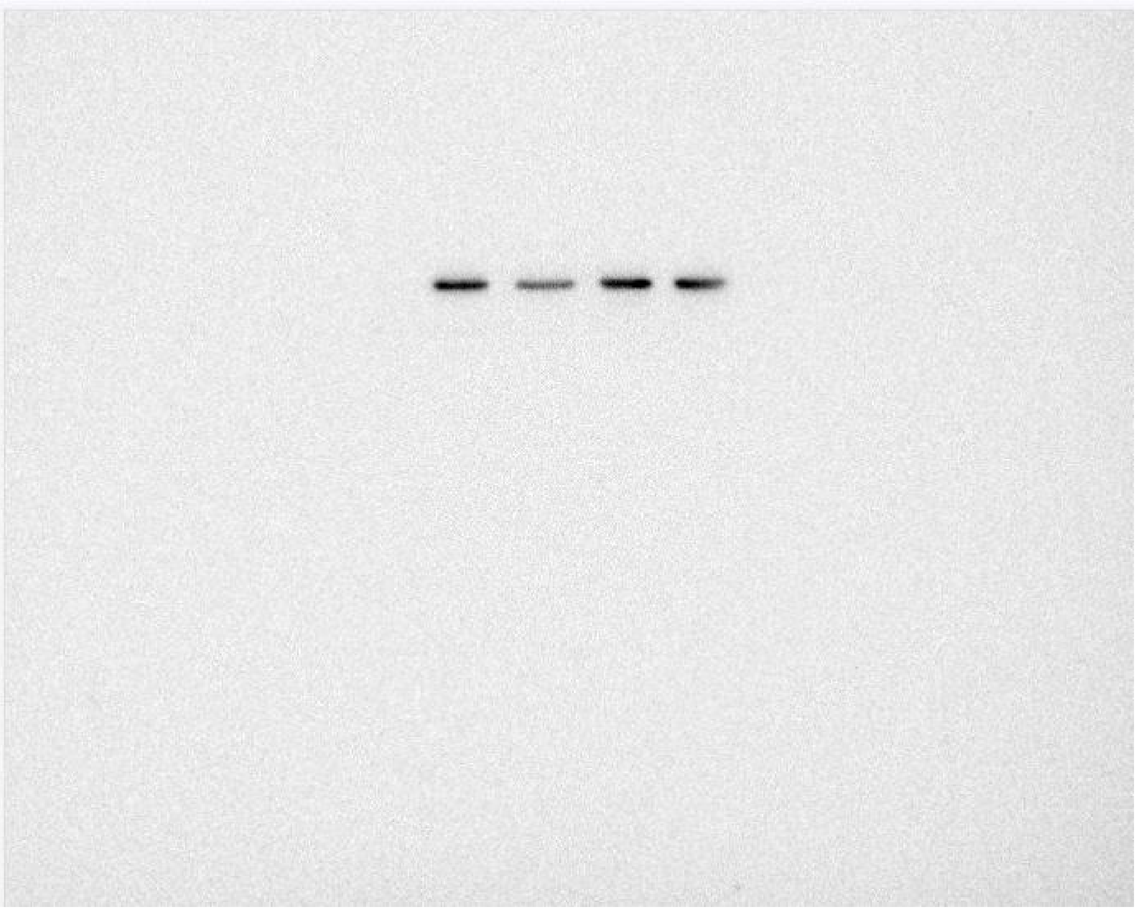

**Figure 3D *CXCL1$^{OE}$***

**β-actin**

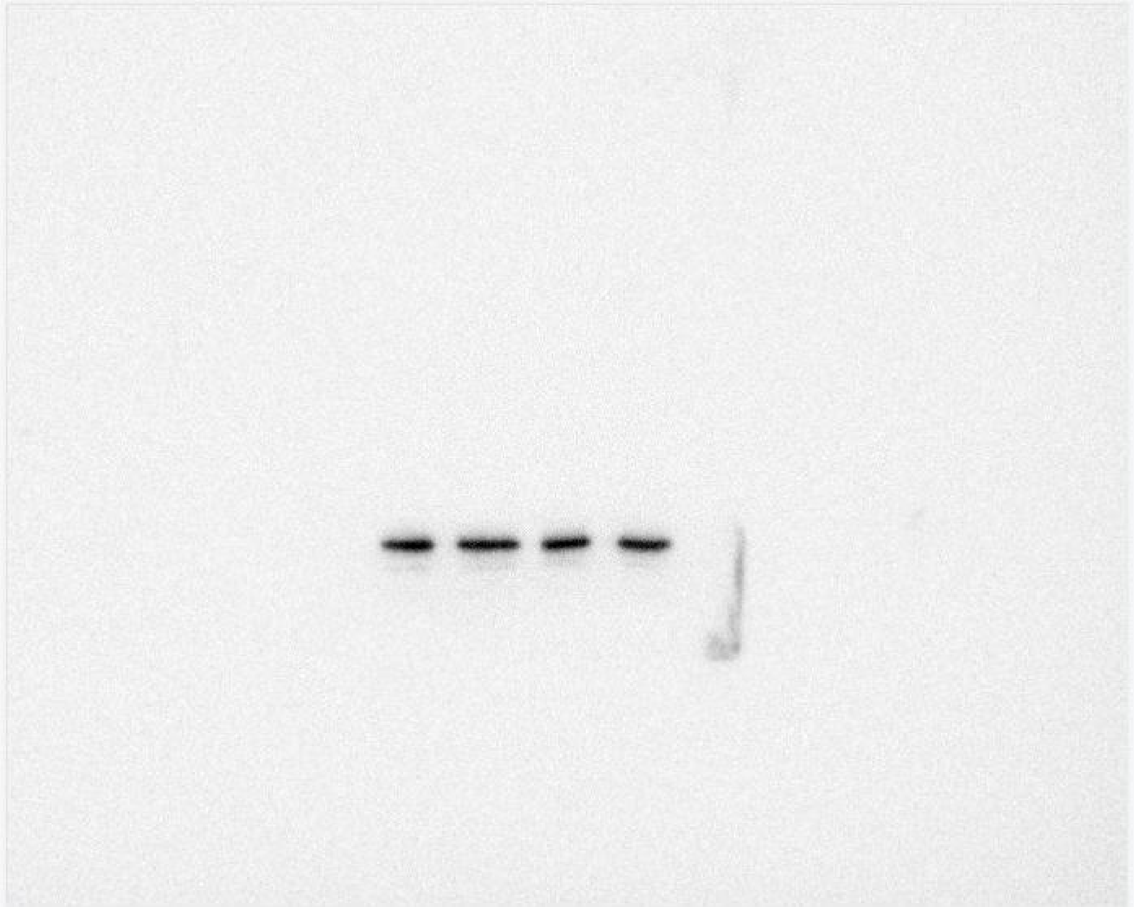

**LC3**

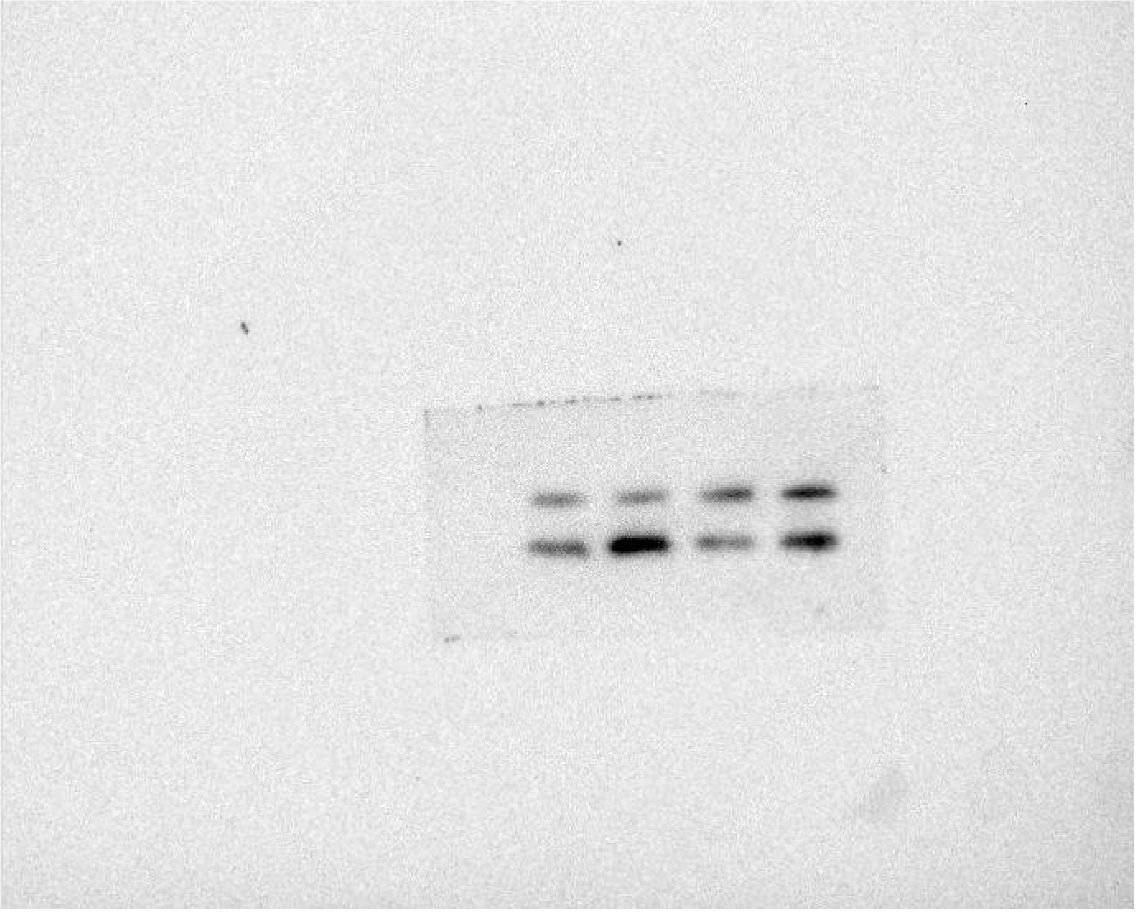

**Figure 3D *CXCL1*$^{KO}$**

ABCG2

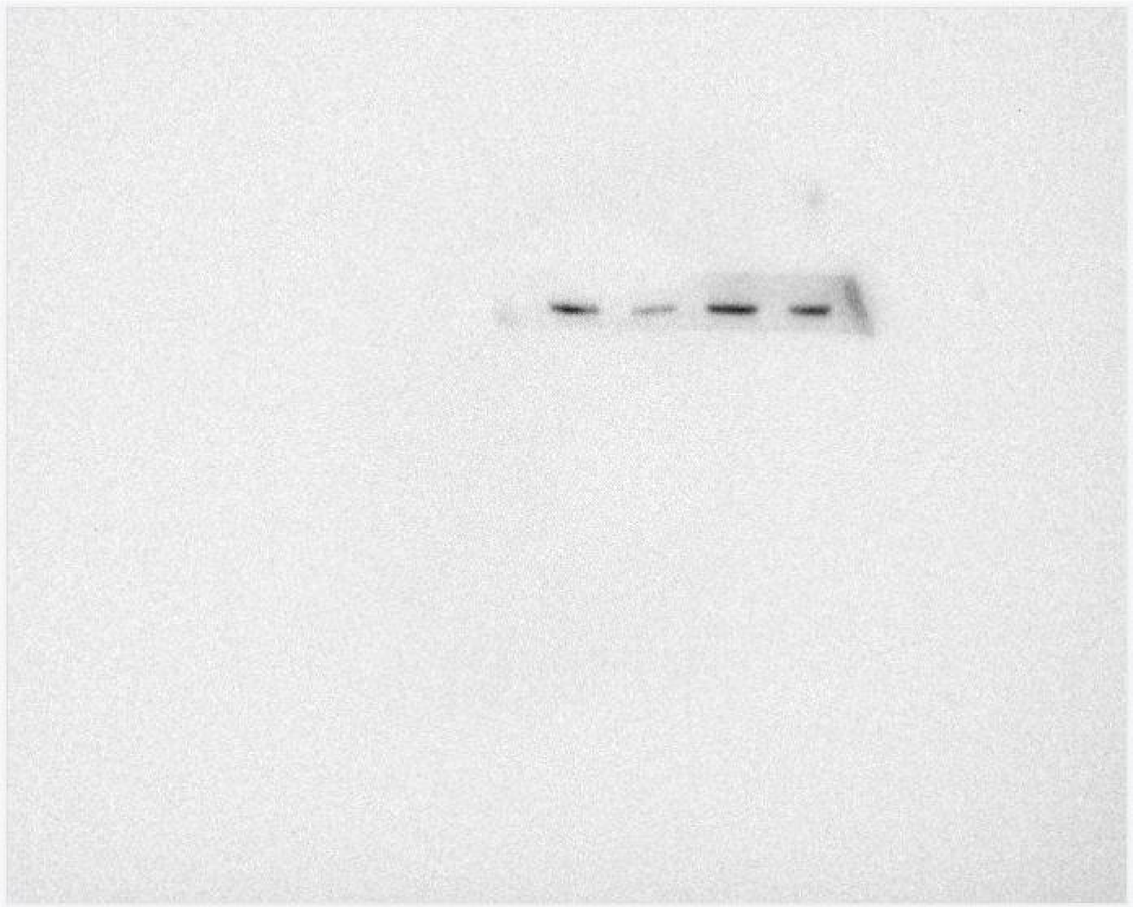

P62

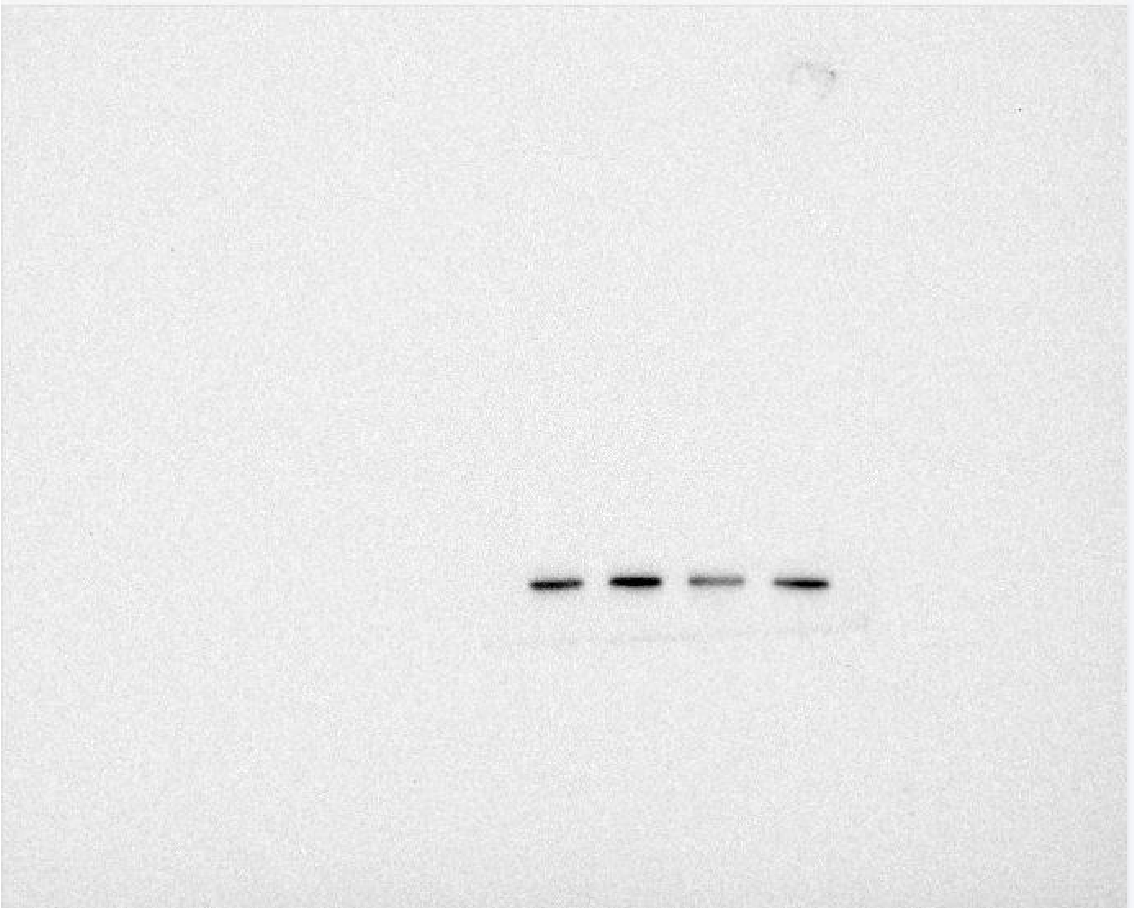

**Figure 3D *CXCL1*$^{KO}$**

β-actin

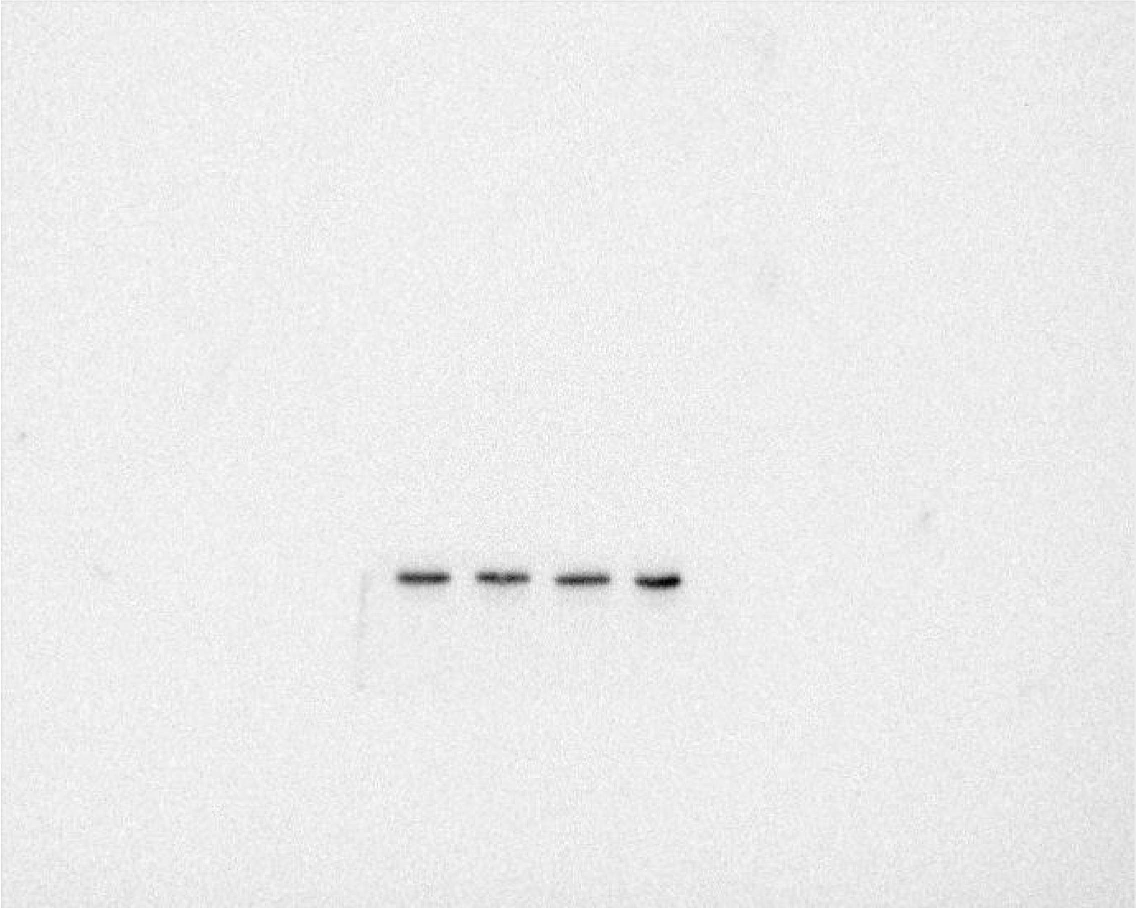

LC3

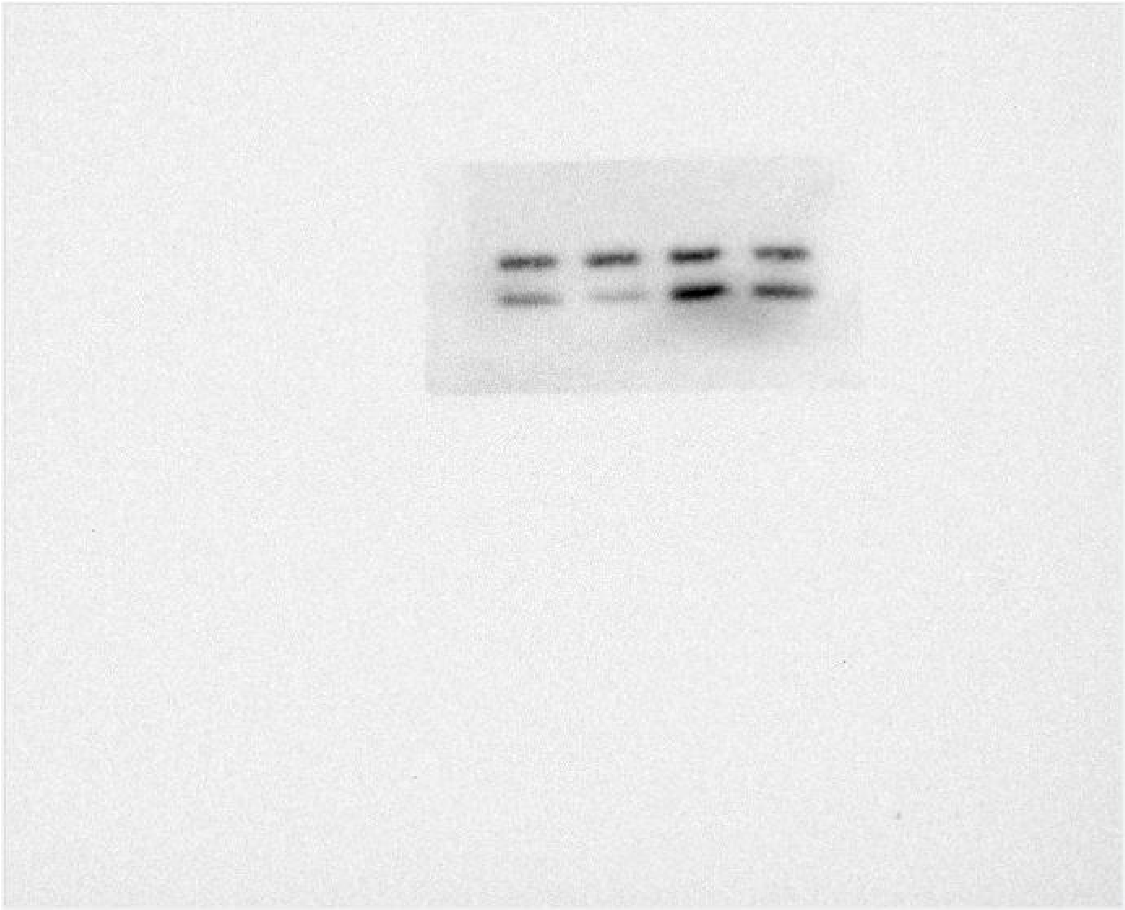

**Figure 4A**

ABCG2

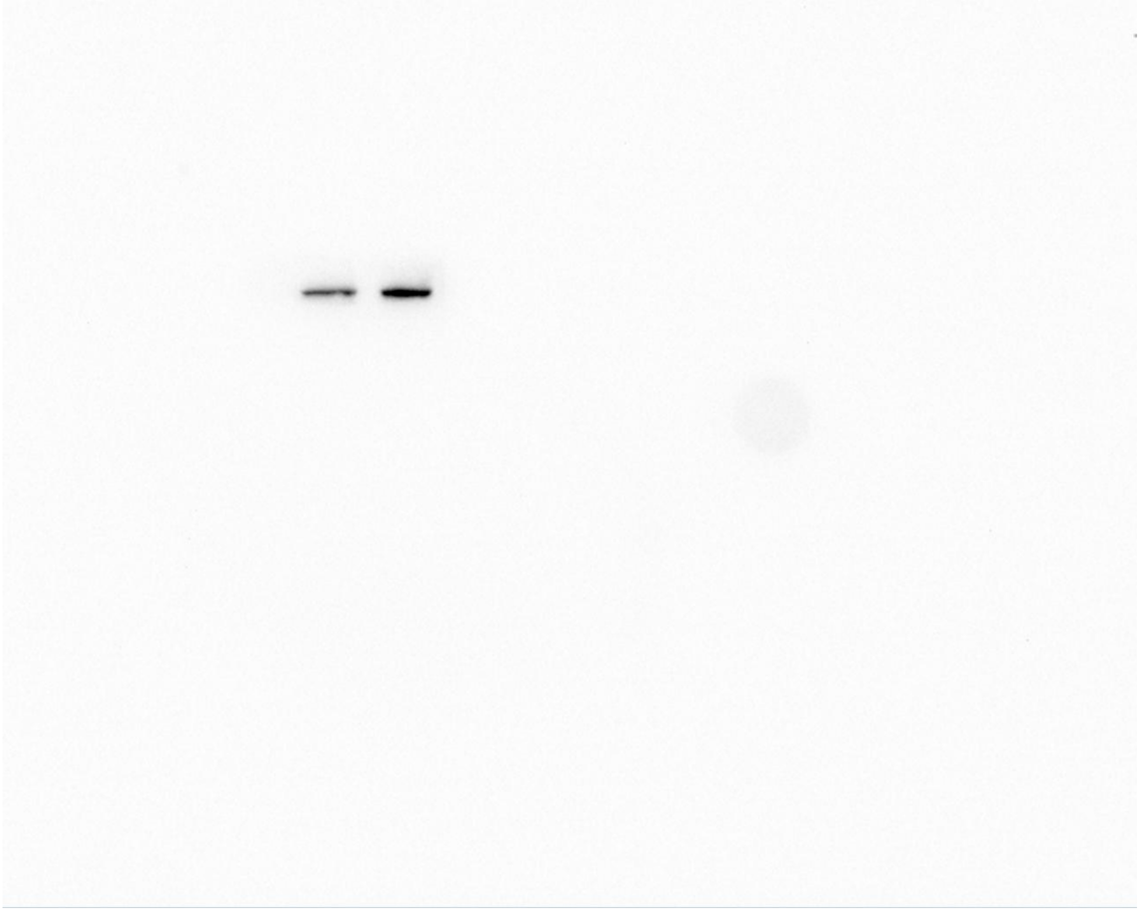

P62

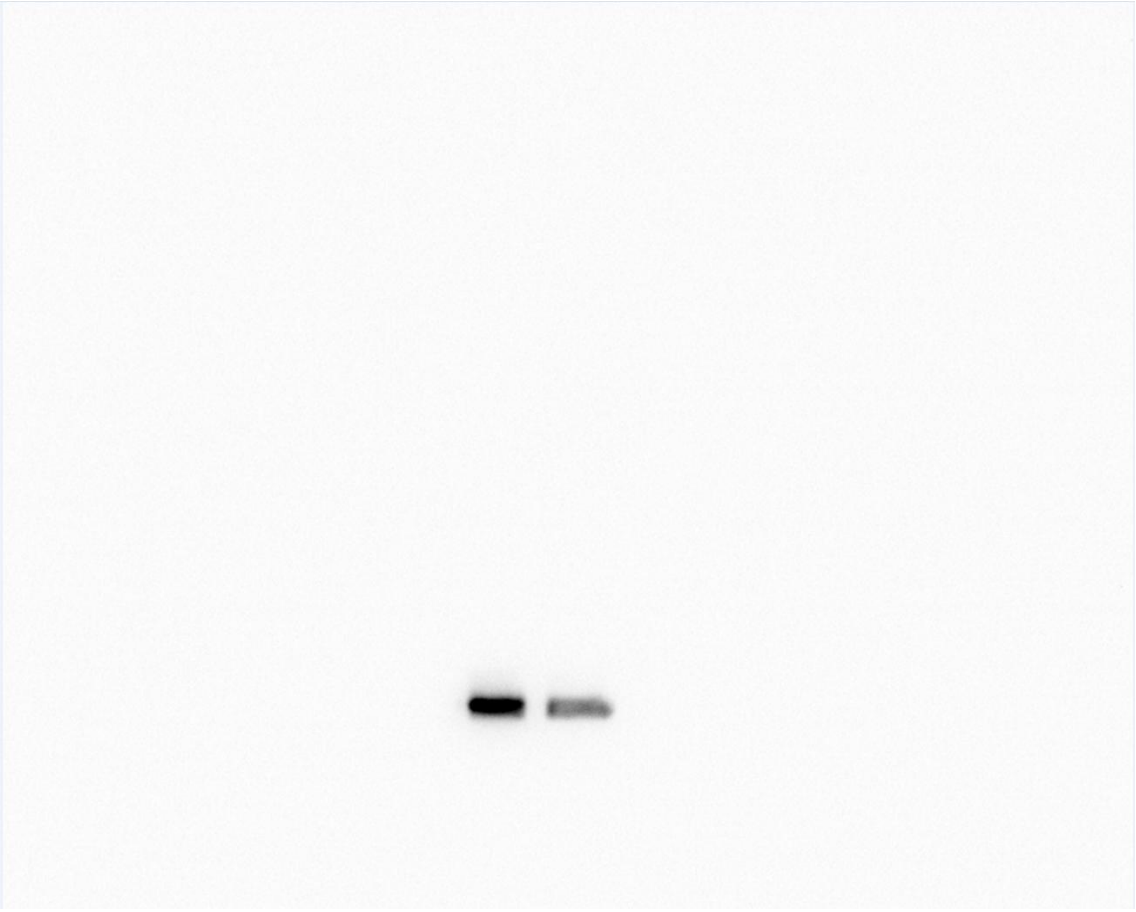

**Figure 4A**

β-actin

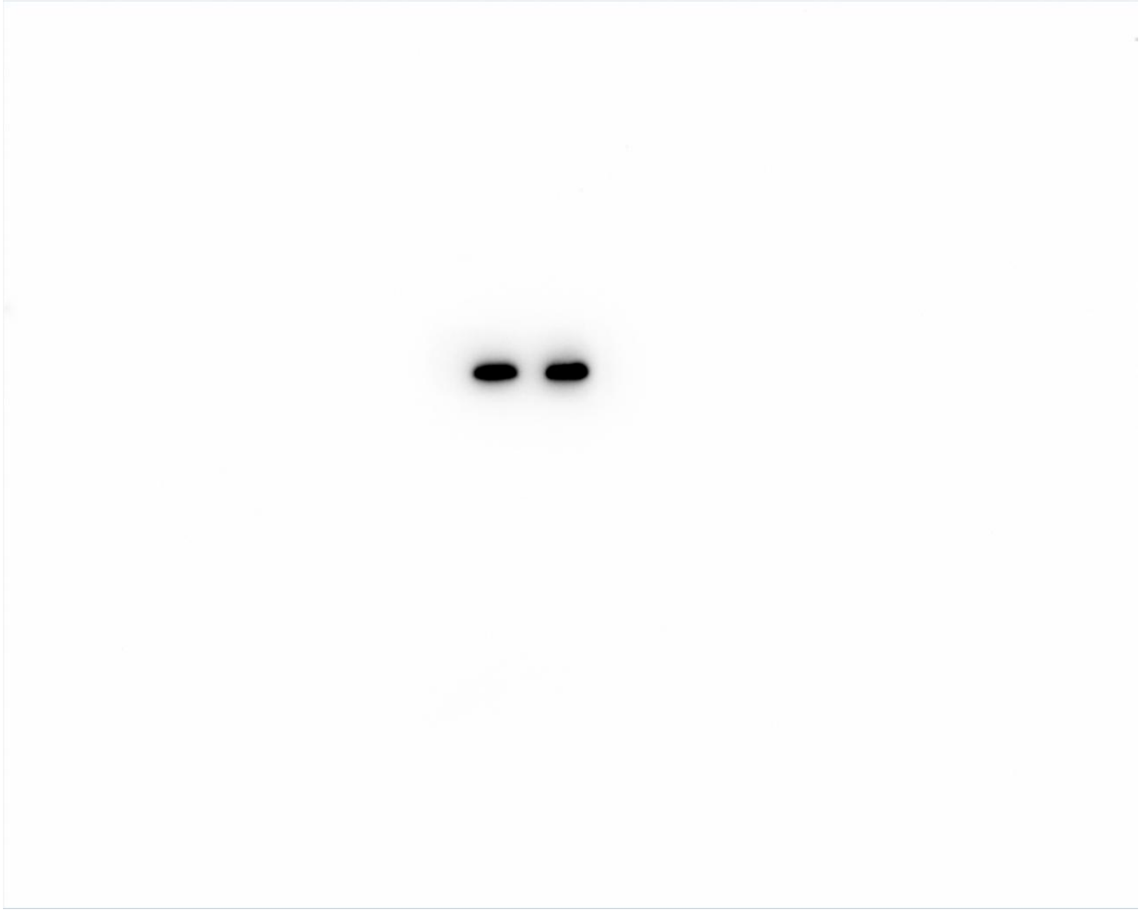

LC-3

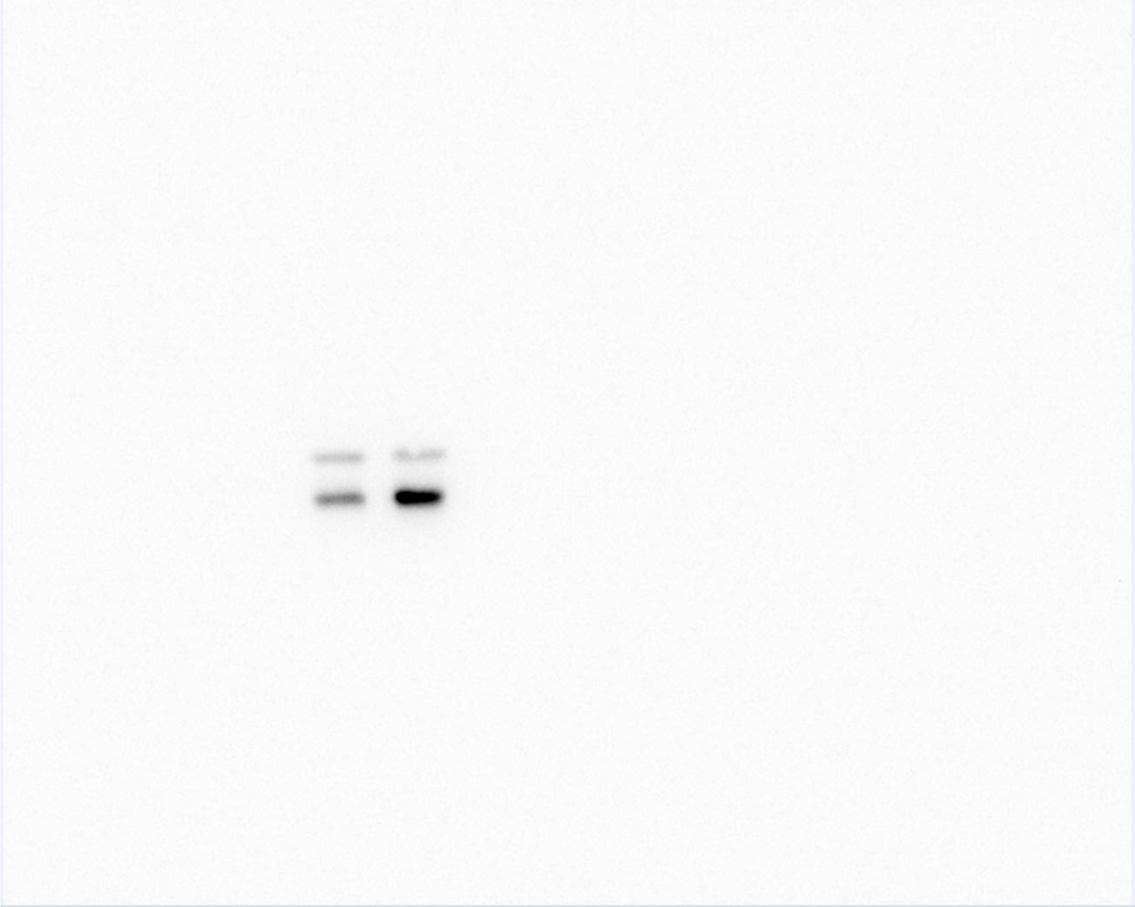

**Figure 4A**

**CXCL1**

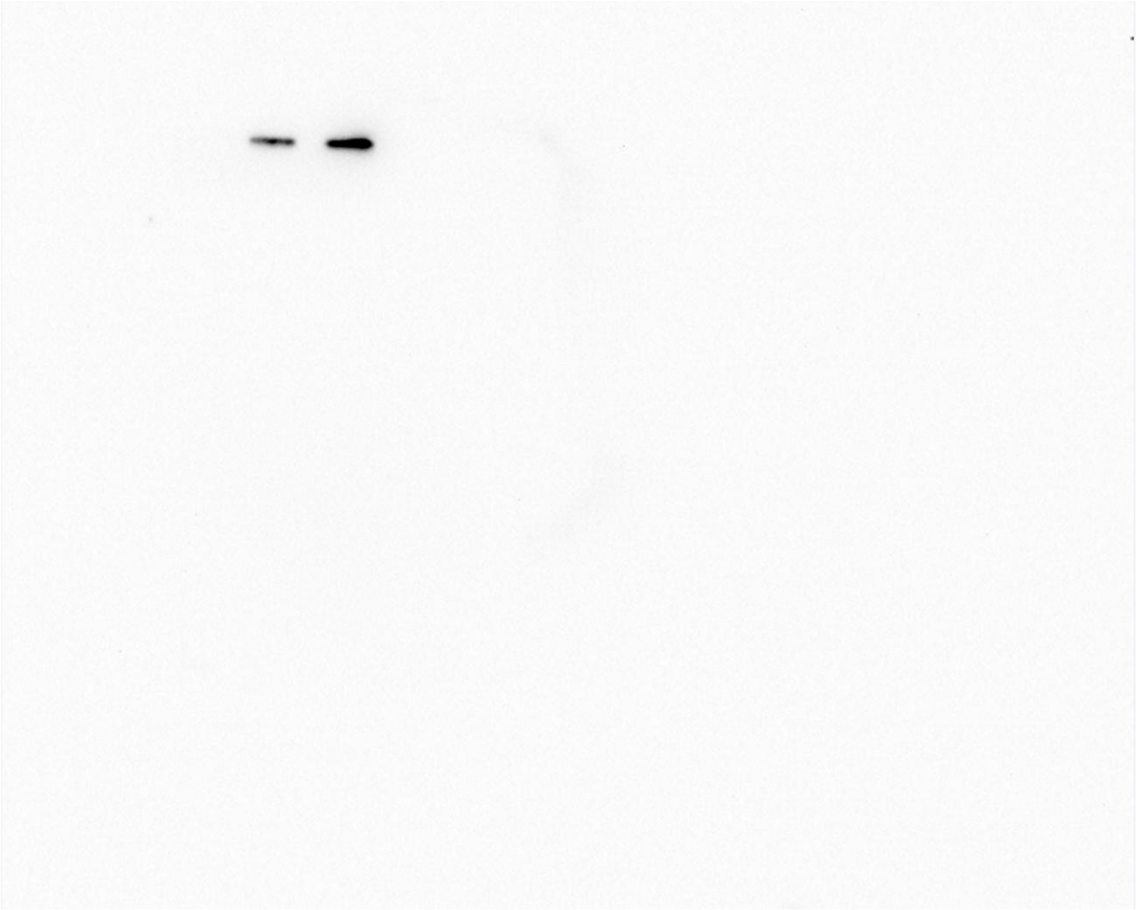

**Figure 4E**

ABCG2

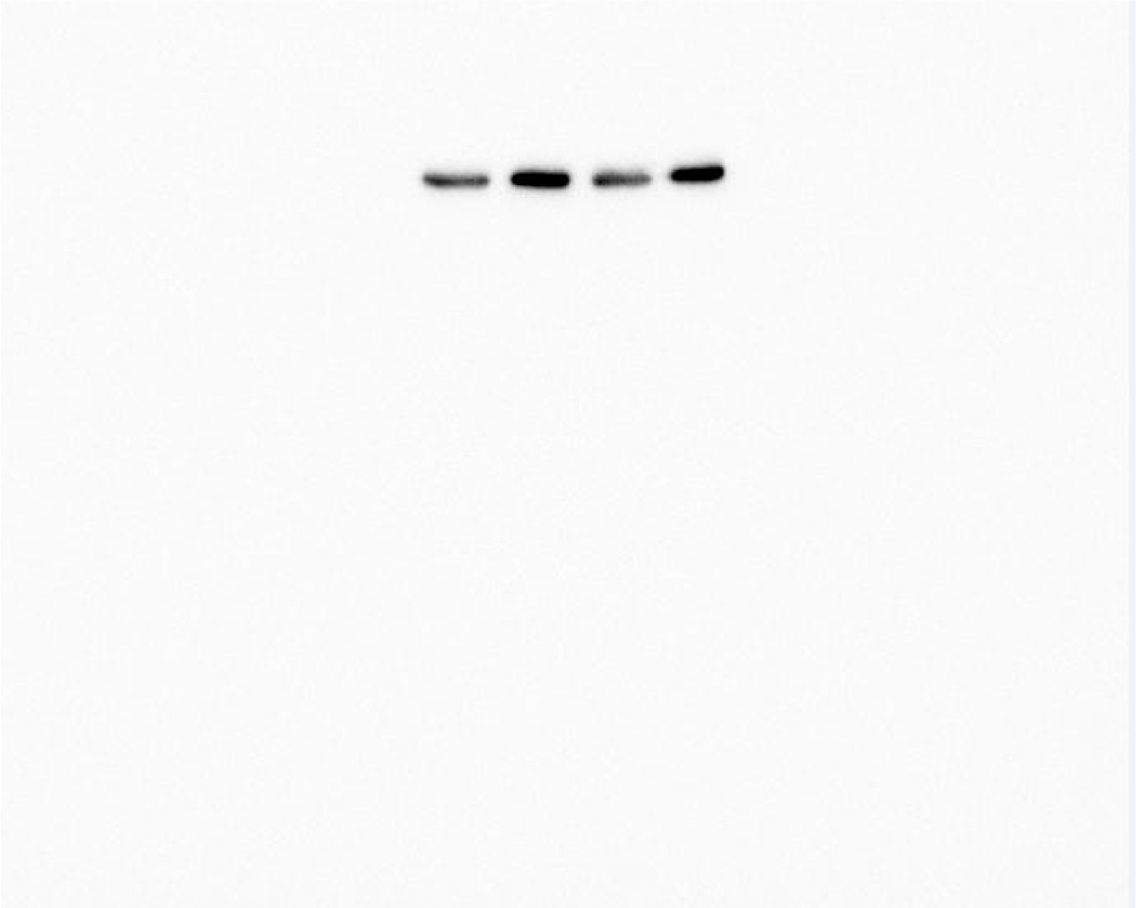

P62

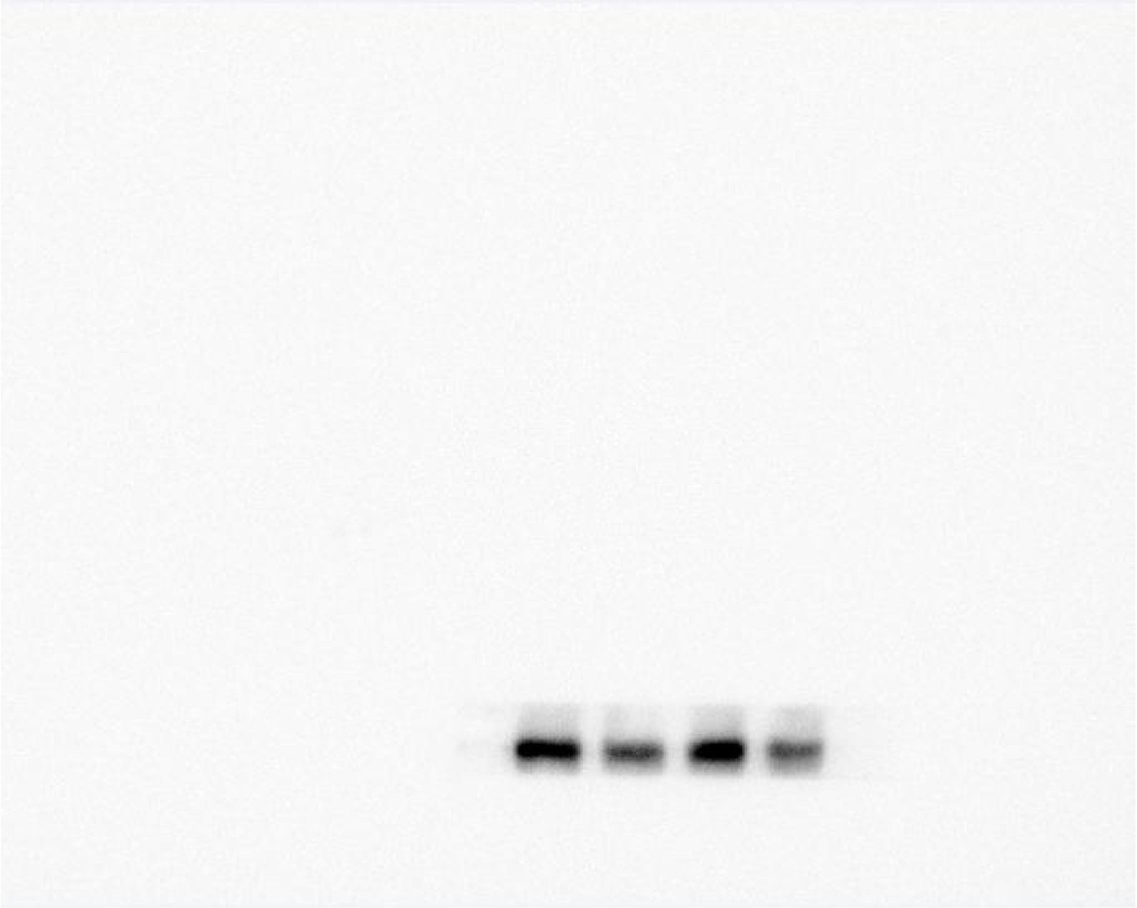

**Figure 4E**

β-actin

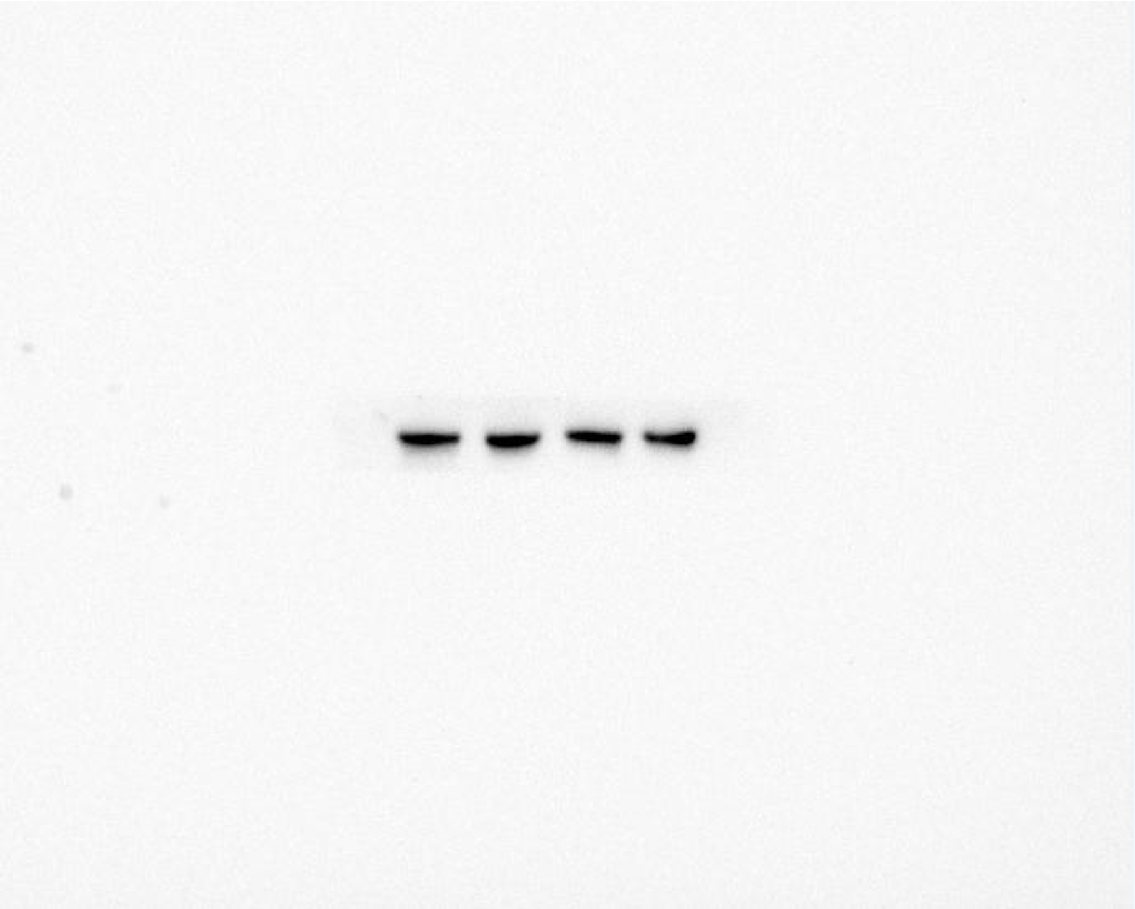

LC3

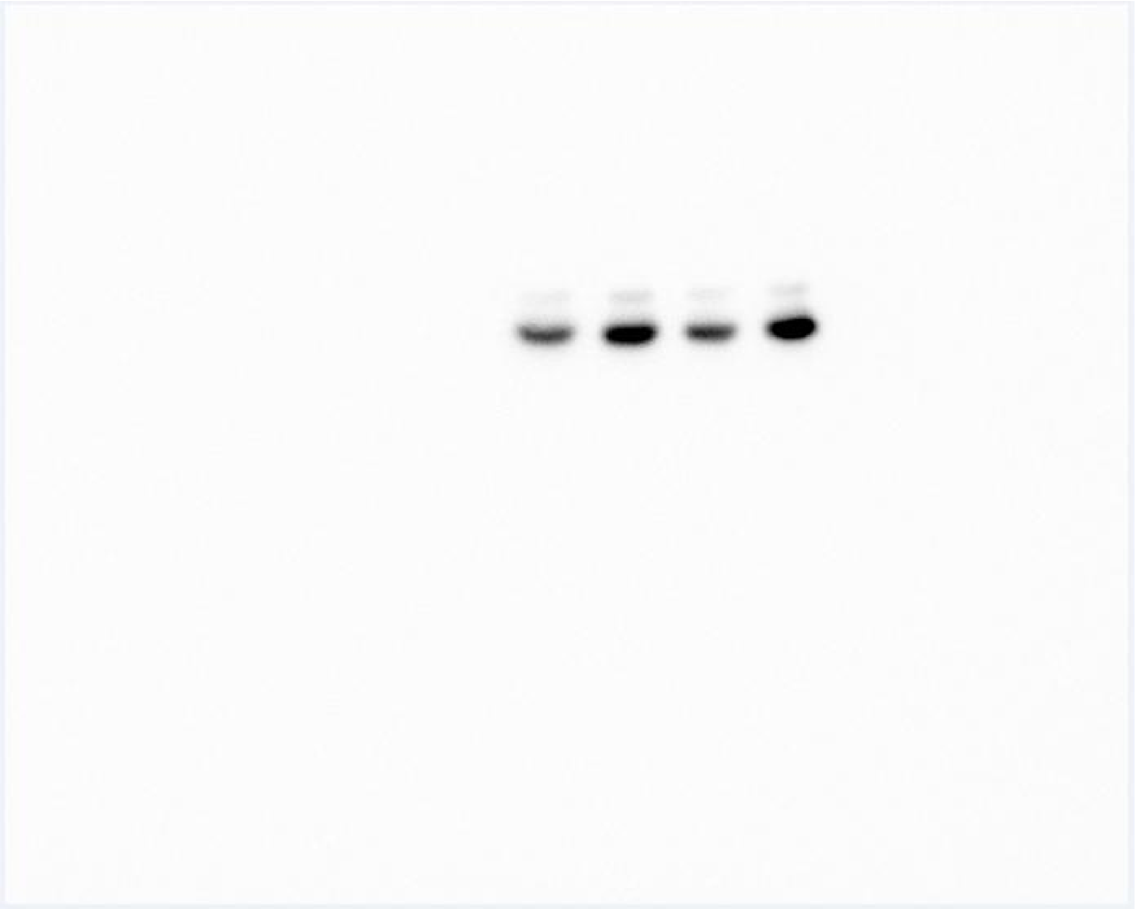

# Figure 4E

CXCL1

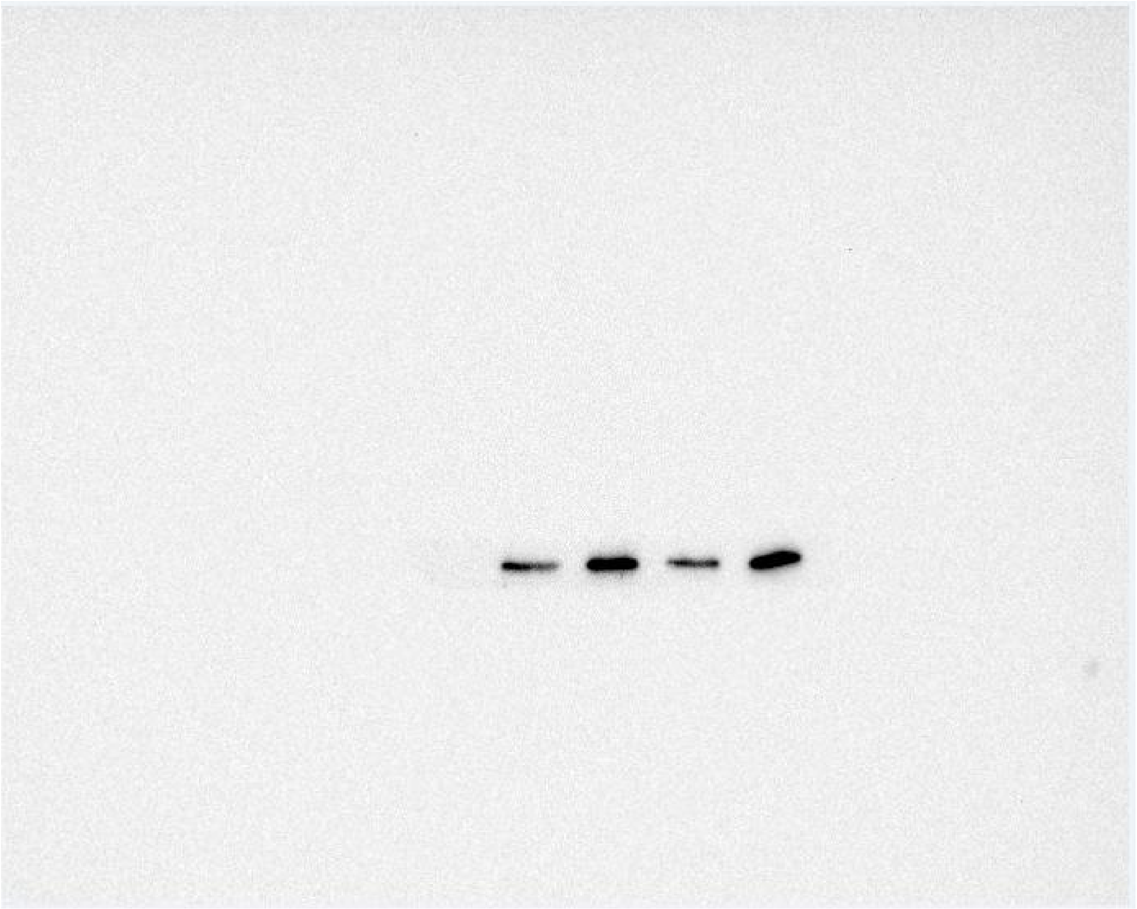

Figure 5B CXCL1

IGF1R

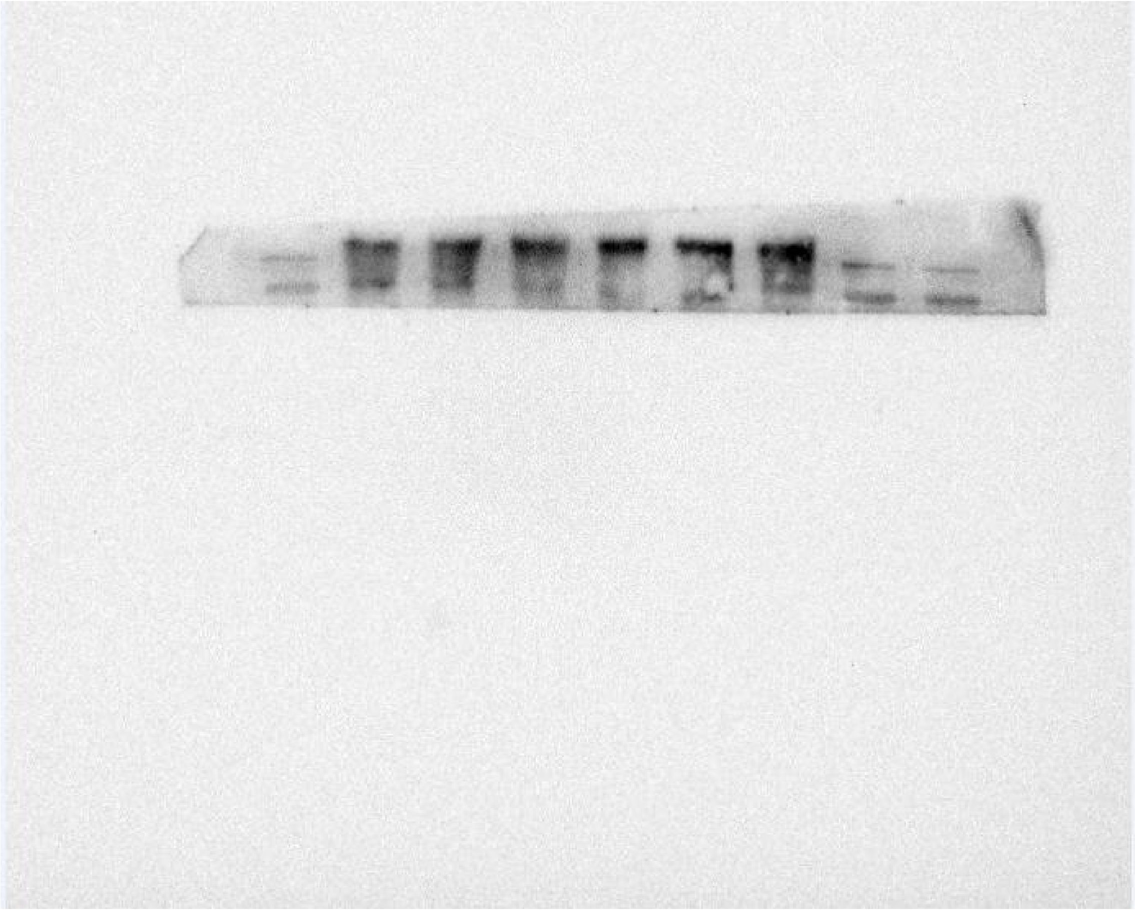

β-actin

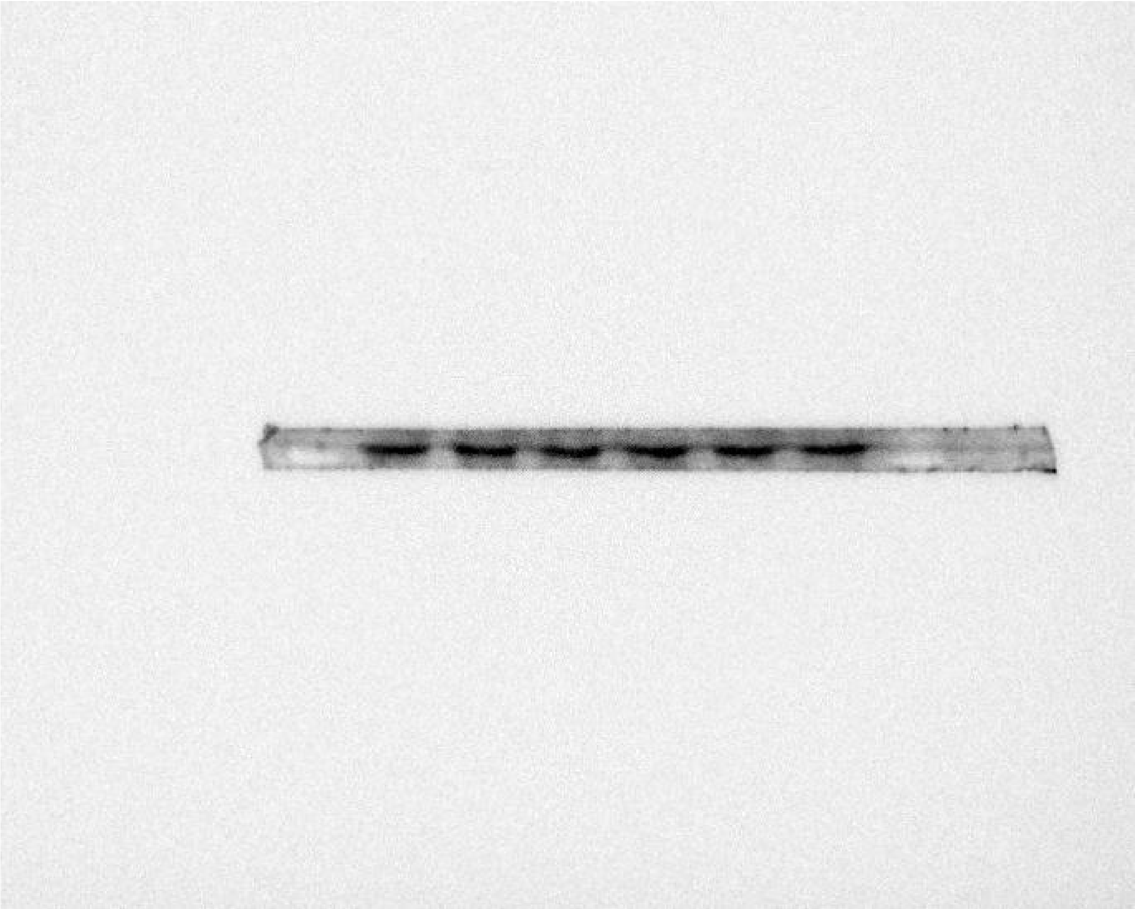

**Figure 5B CXCL1**

IGF1

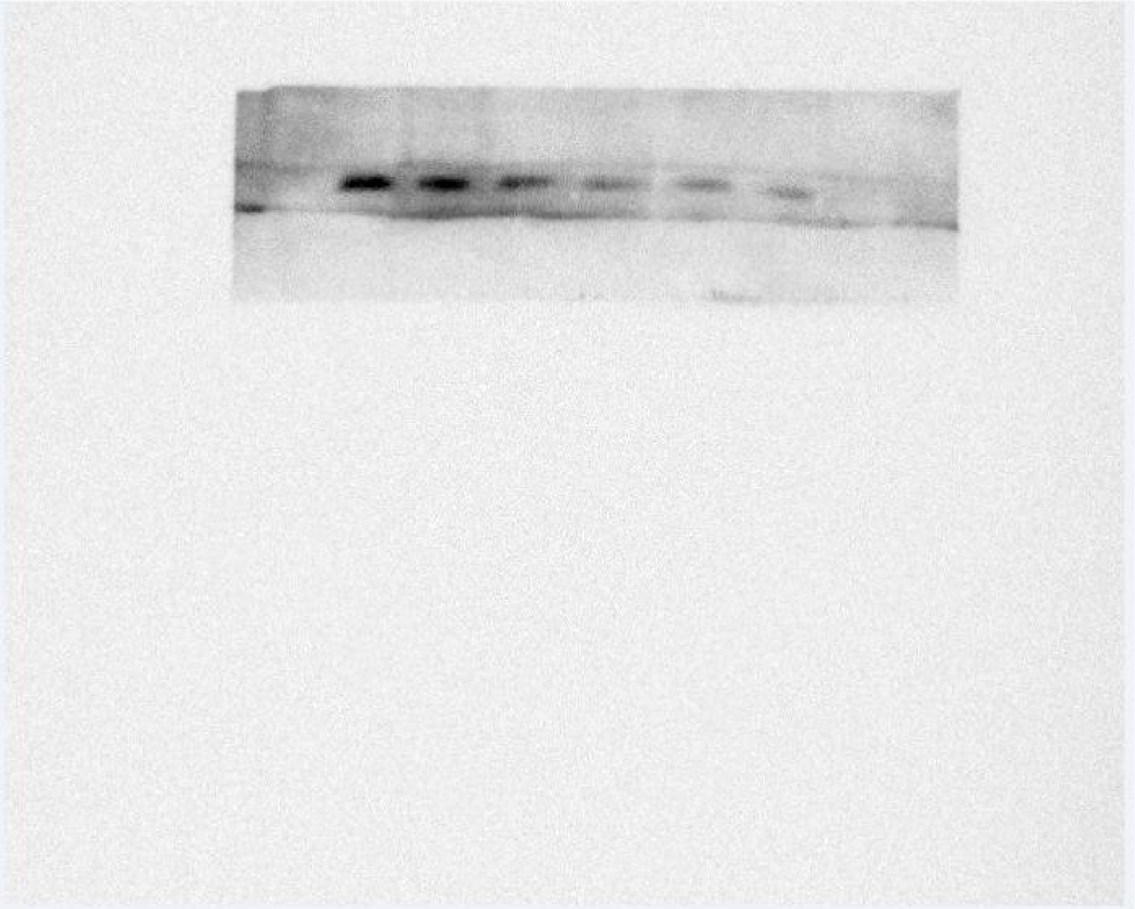

**Figure 5B *CXCL1*$^{OE}$**

**IGF1R**

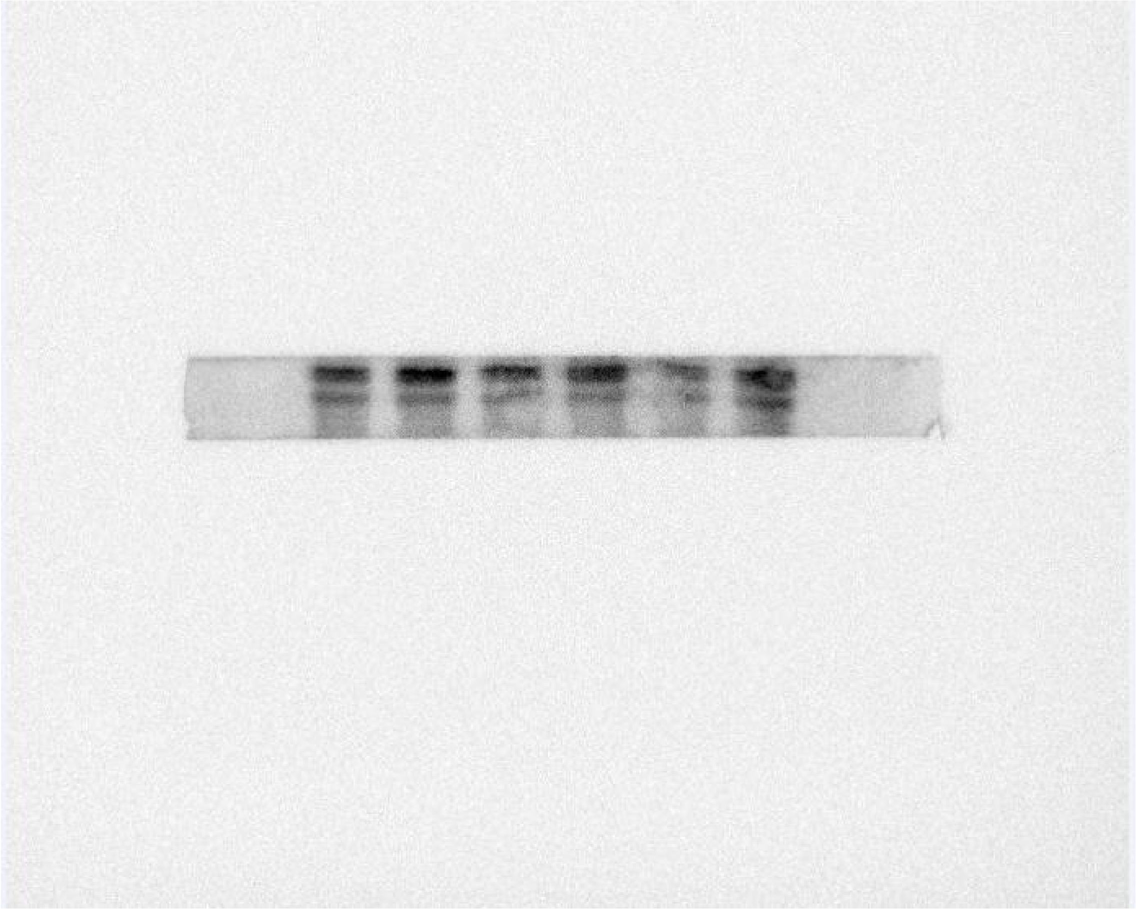

**β-actin**

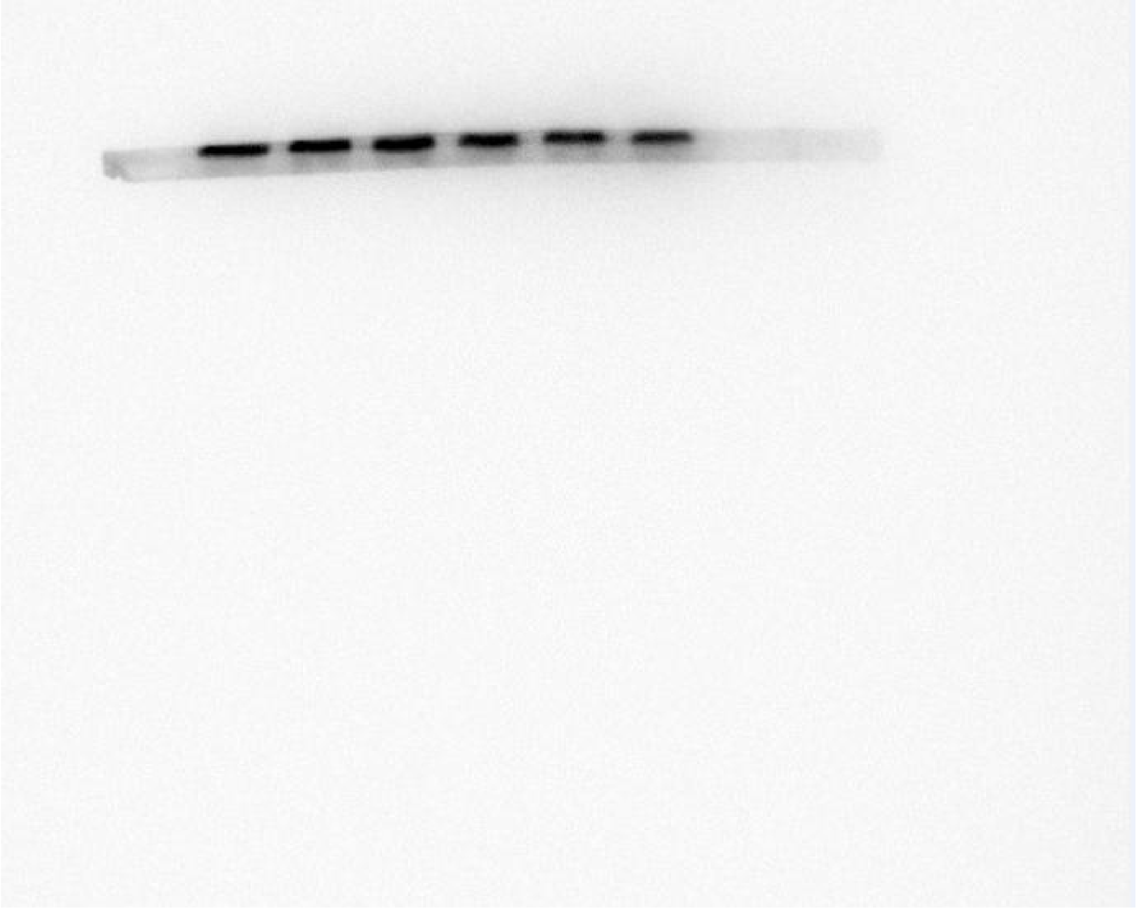

# Figure 5B *CXCL1*$^{OE}$

IGF1

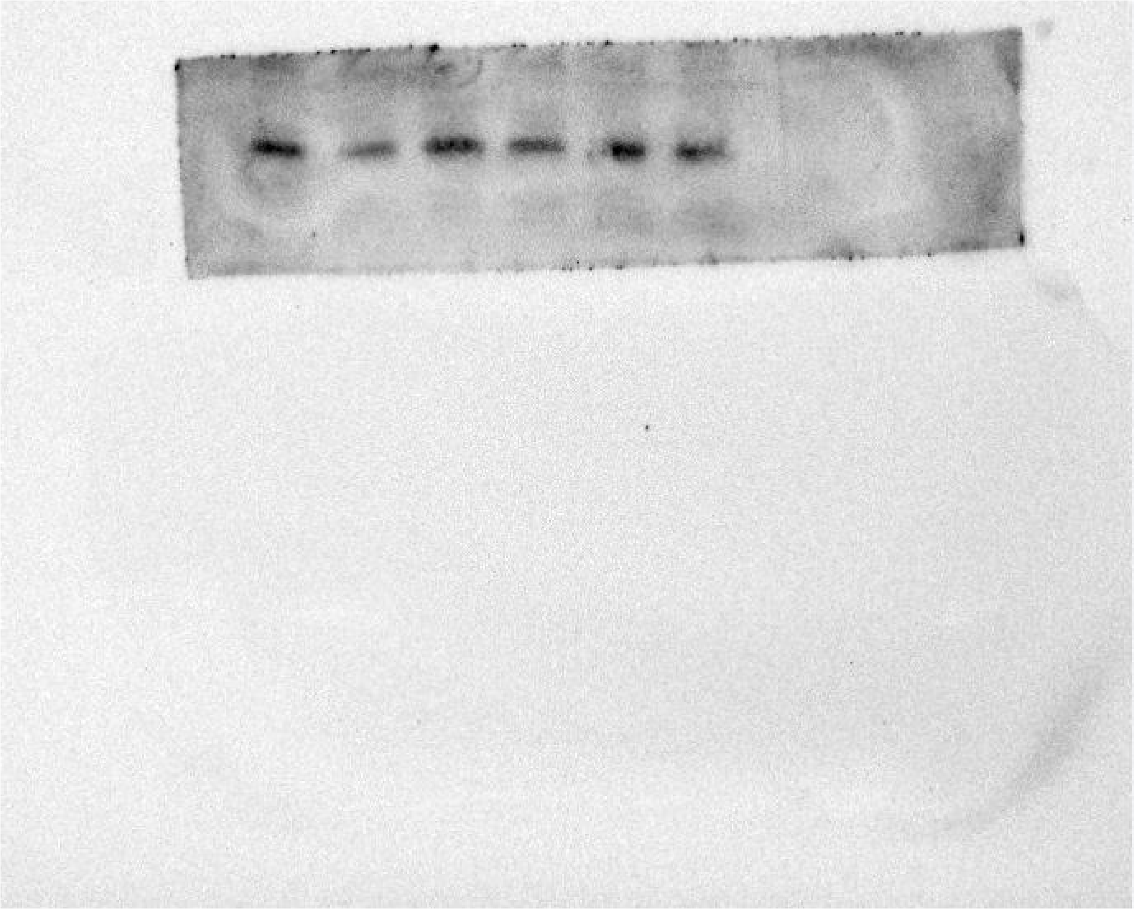

**Figure 5B *CXCL1*$^{KO}$**

**IGF1R**

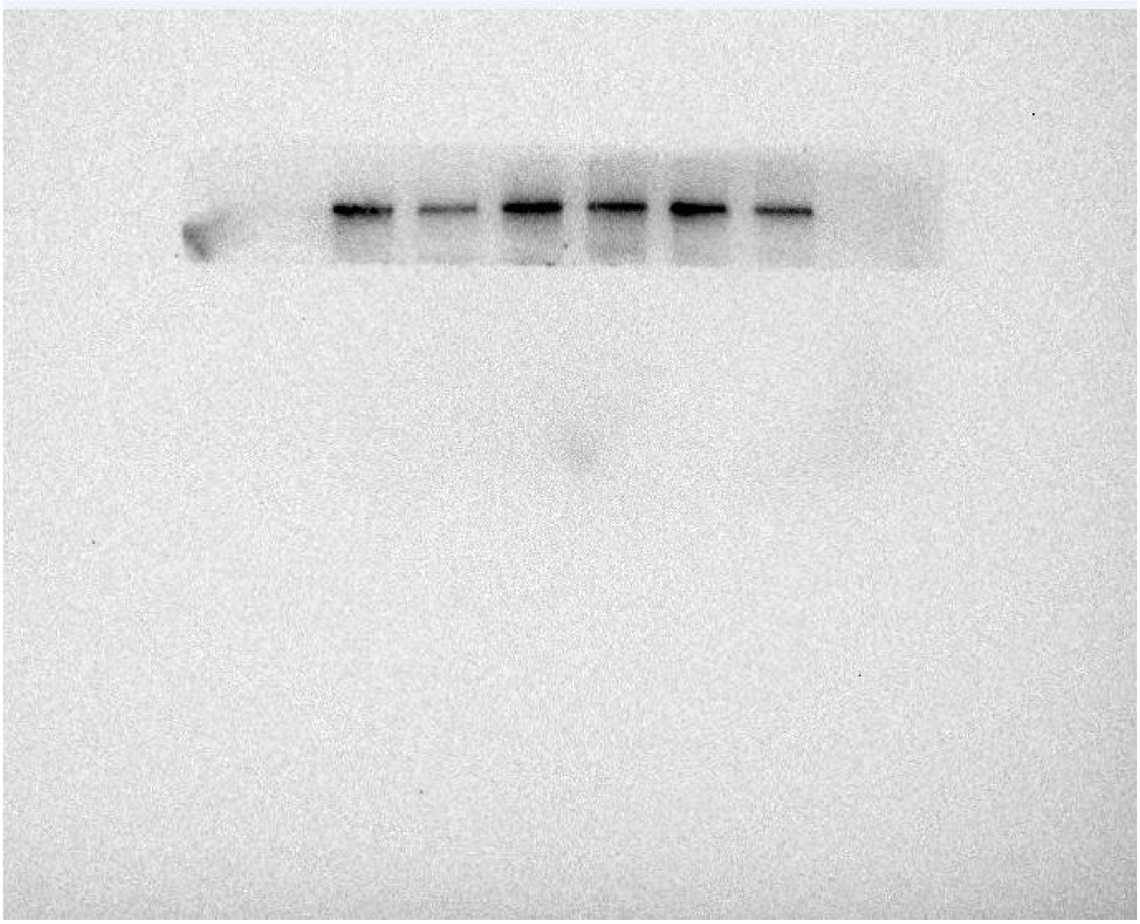

**β-actin**

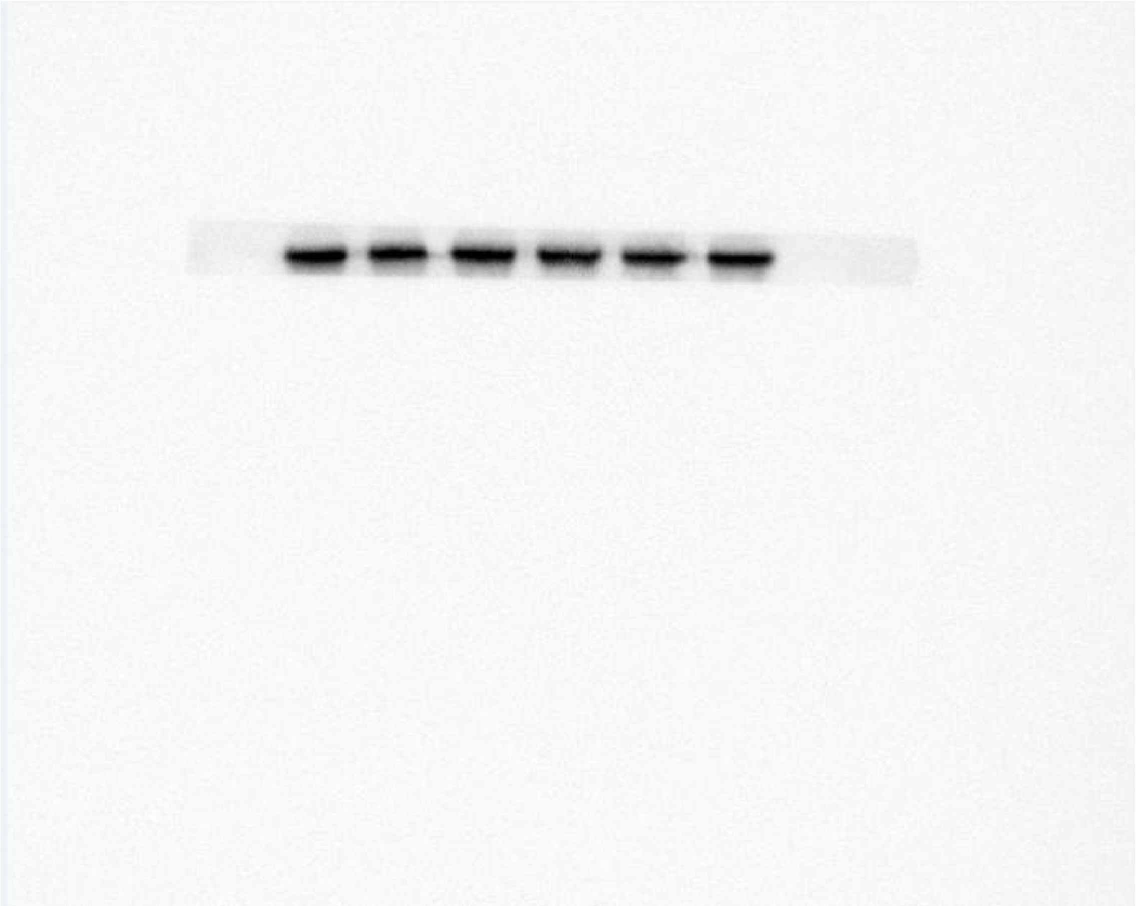

**Figure 5B *CXCL1*$^{KO}$**

IGF1

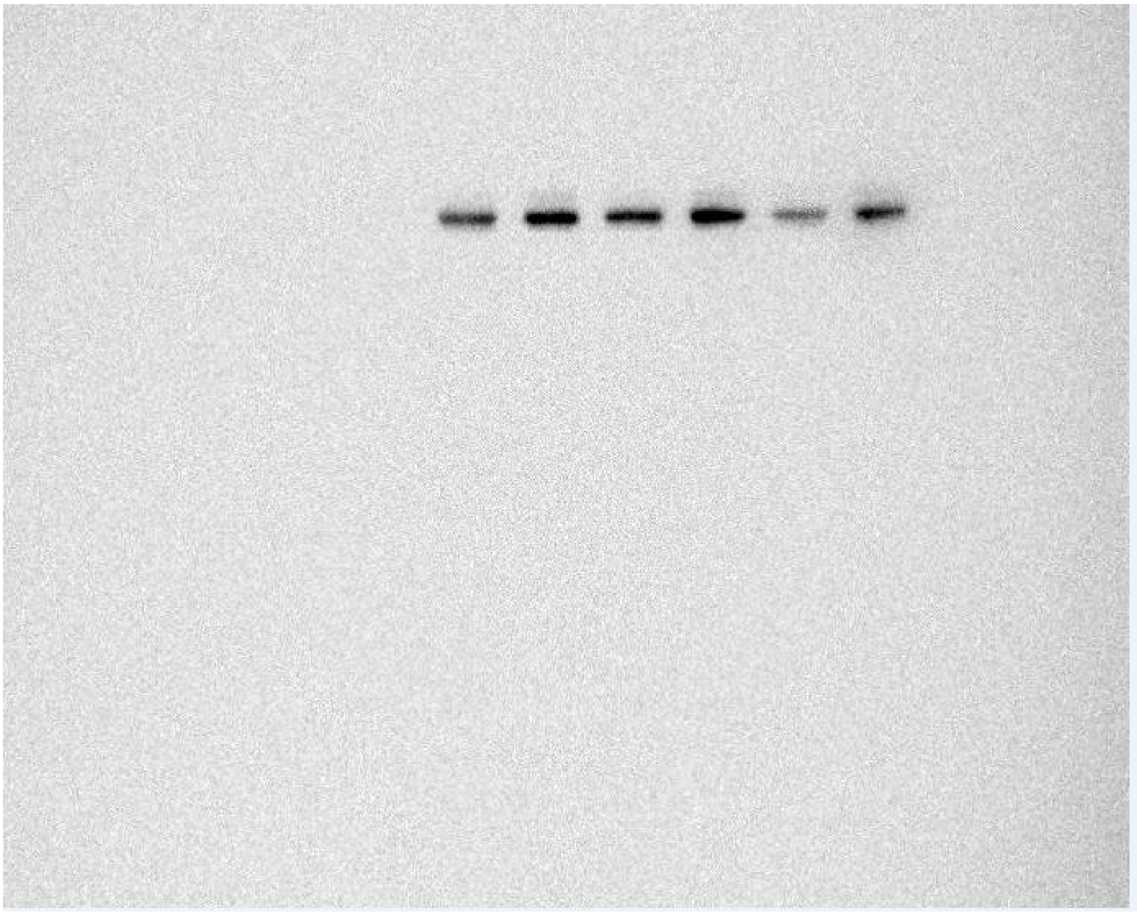

**Figure 5F**

IGF1R

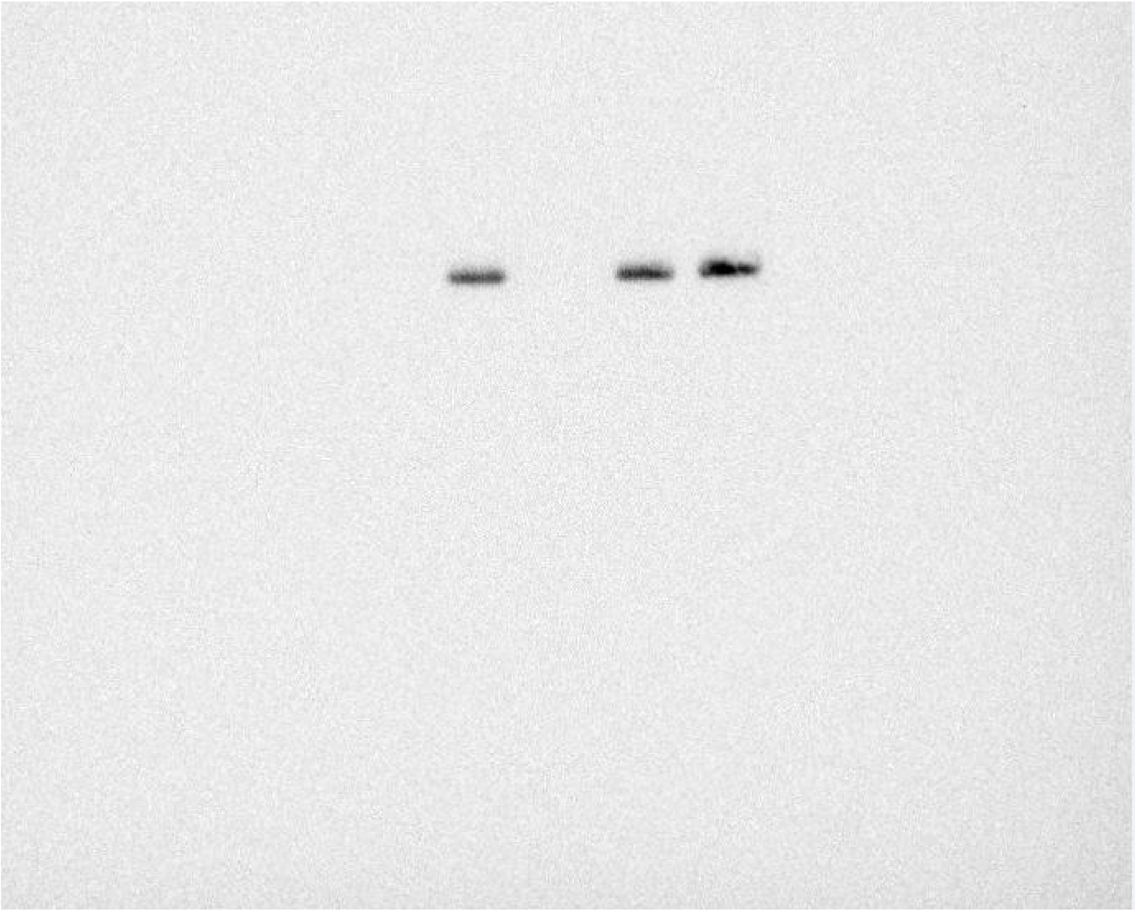

IGF1

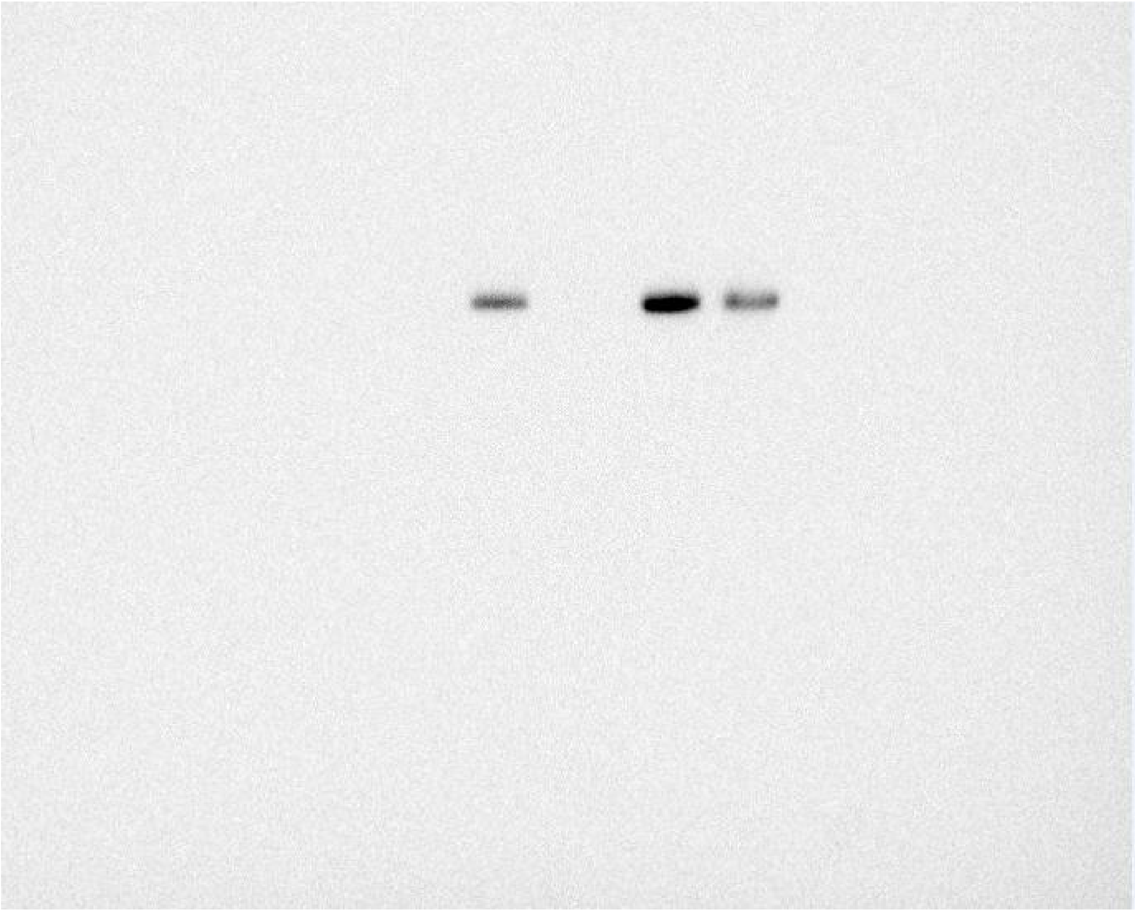

FIG 6A CXCL1

p-STAT3

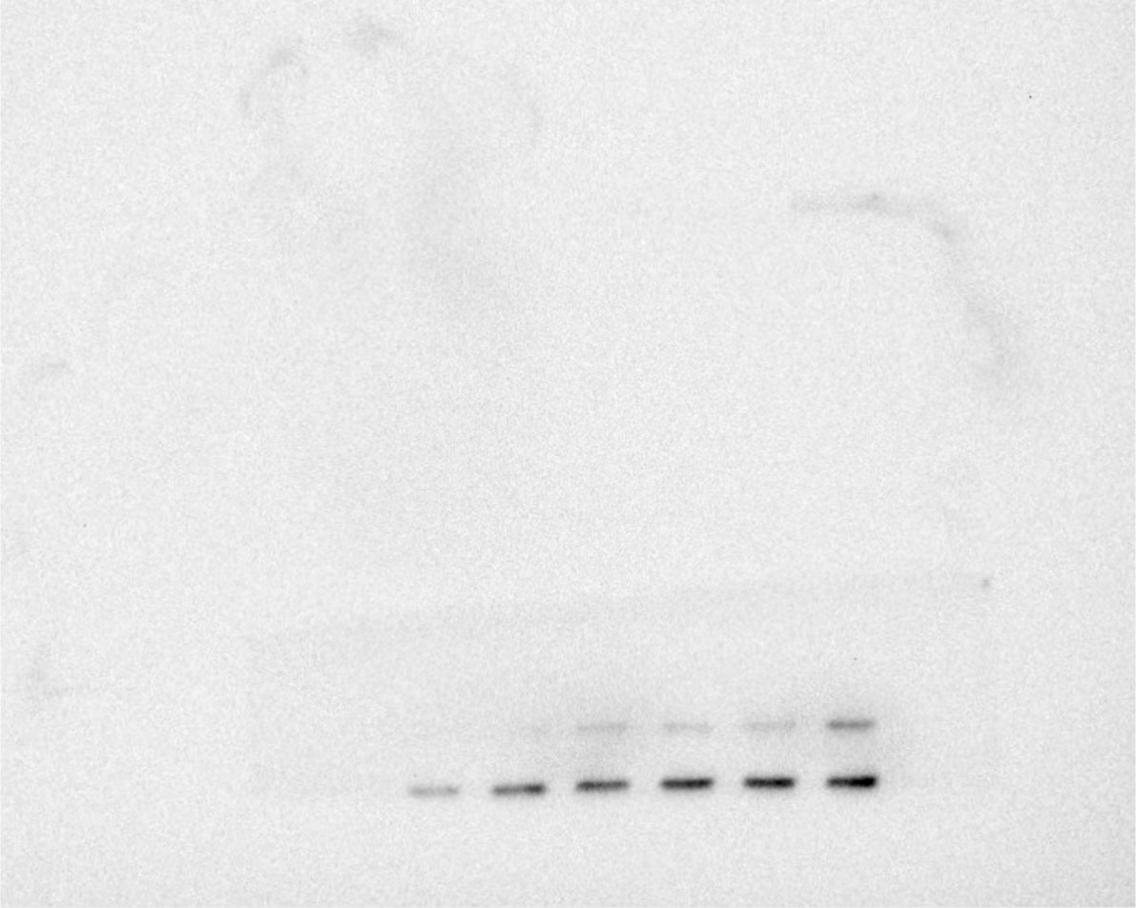

STAT3

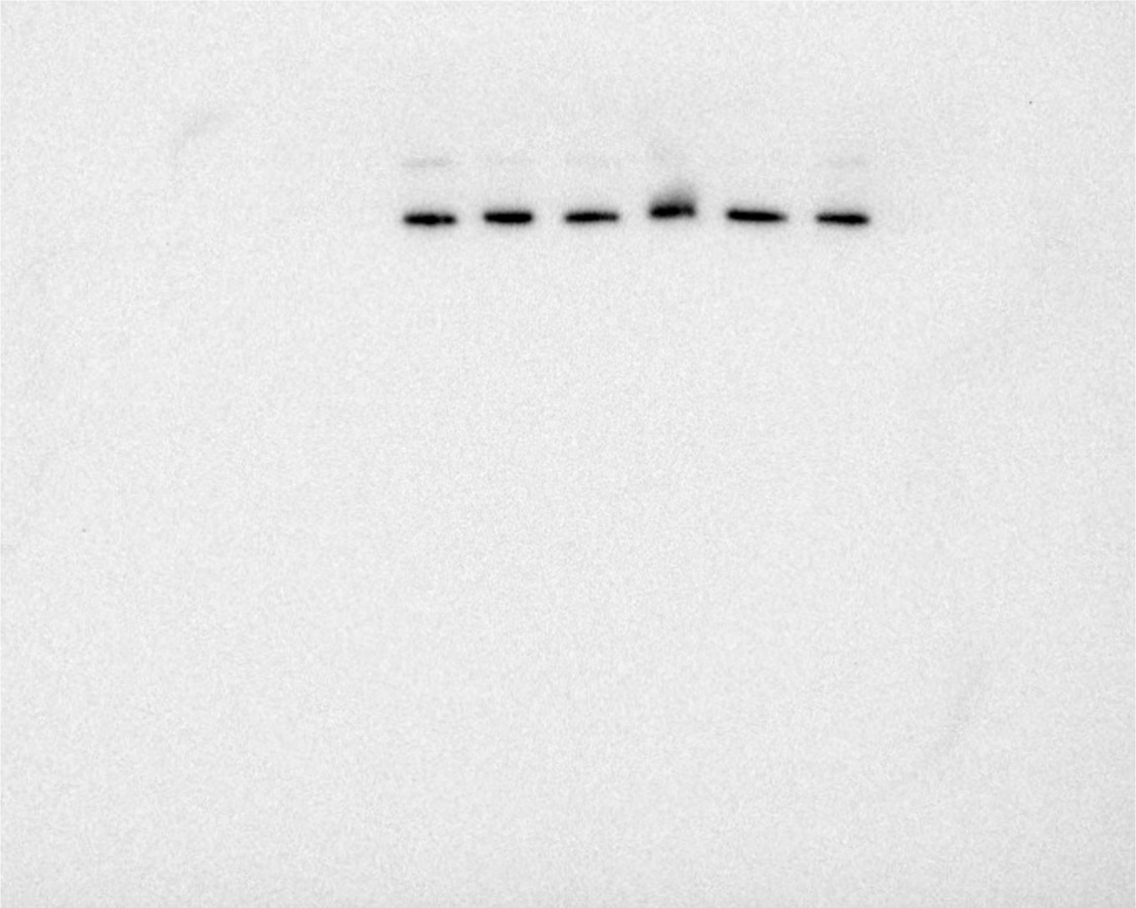

FIG 6A CXCL1

β-actin

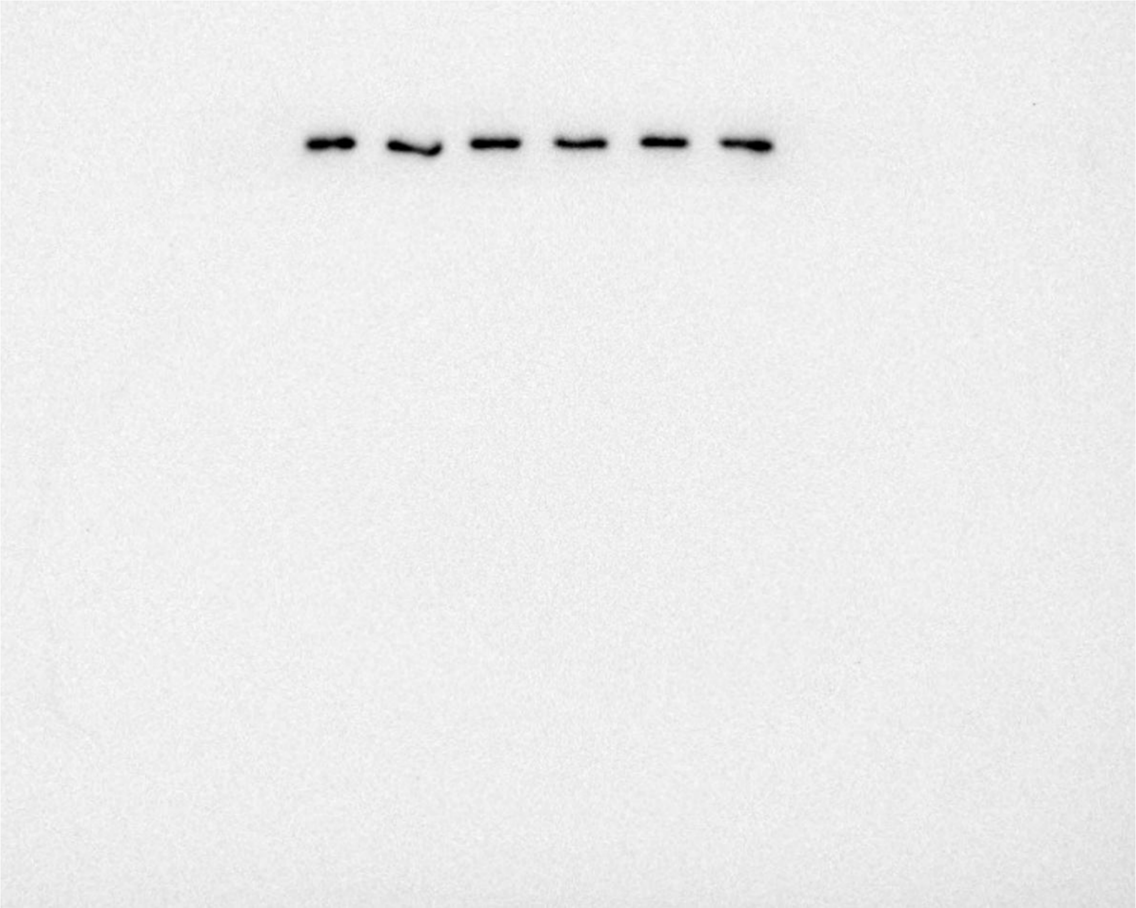

HMGB1

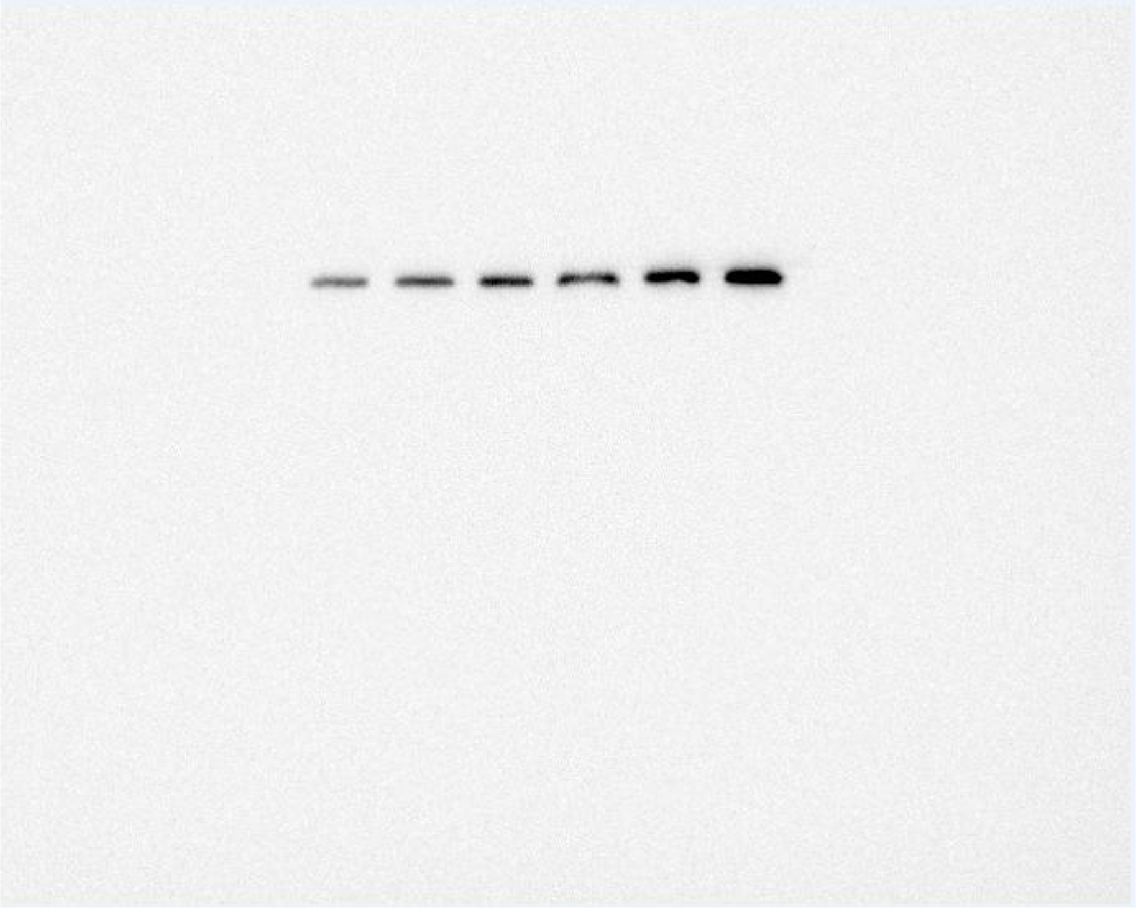

FIG 6A CTS

p-STAT3

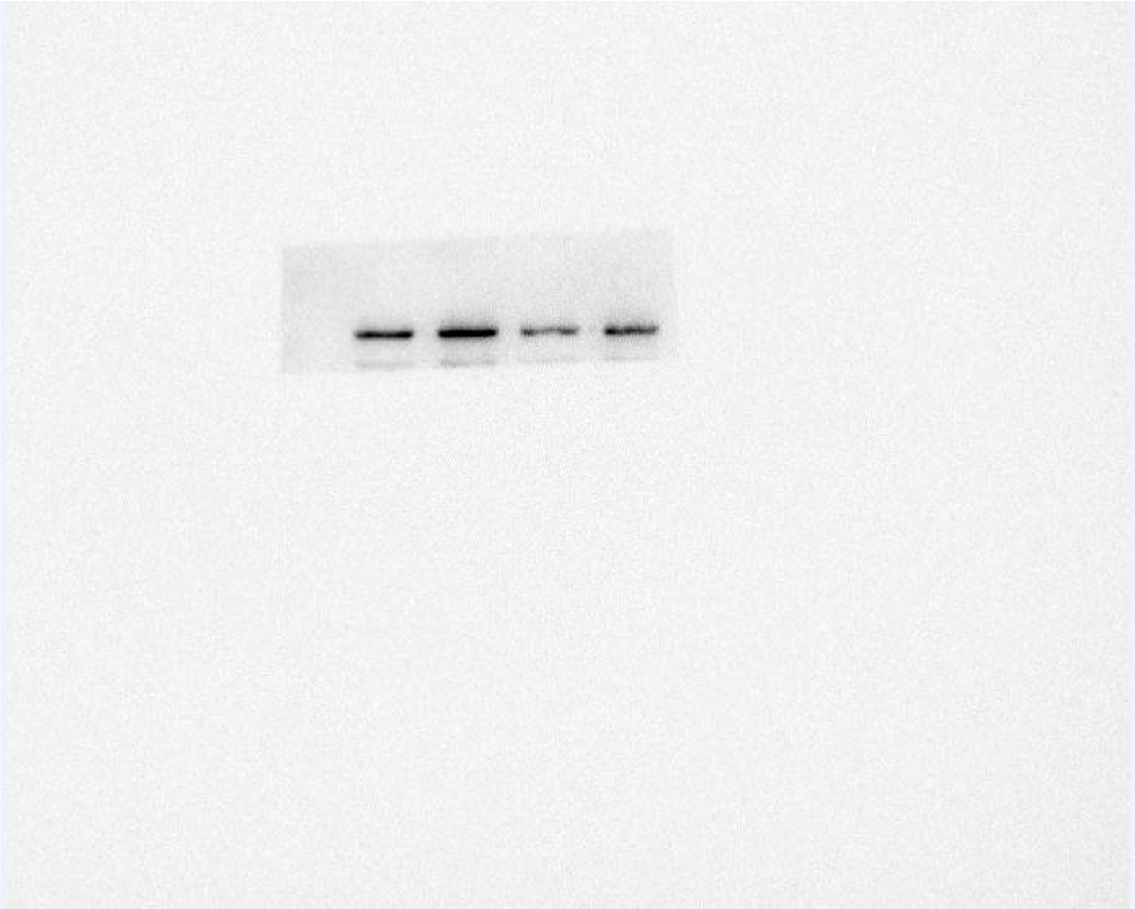

STAT3

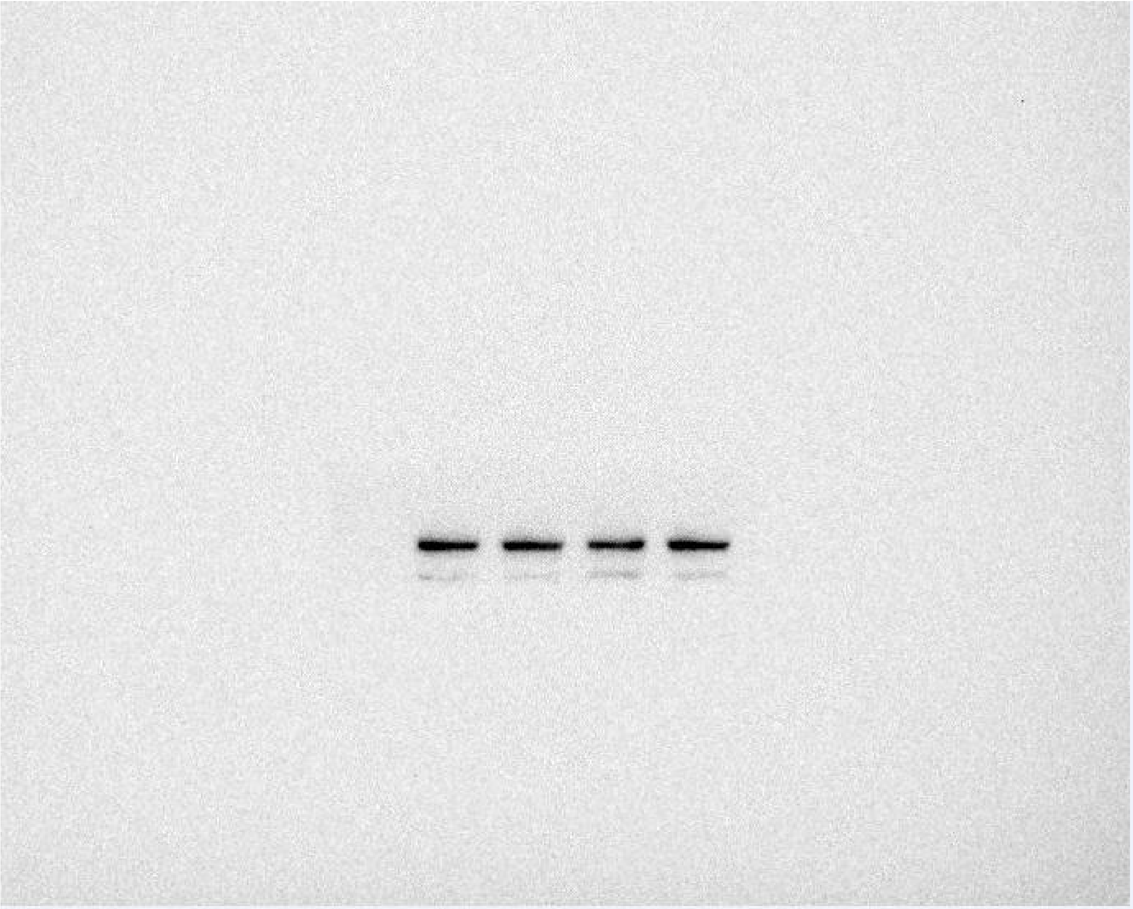

**FIG 6A CTS**

**P62**

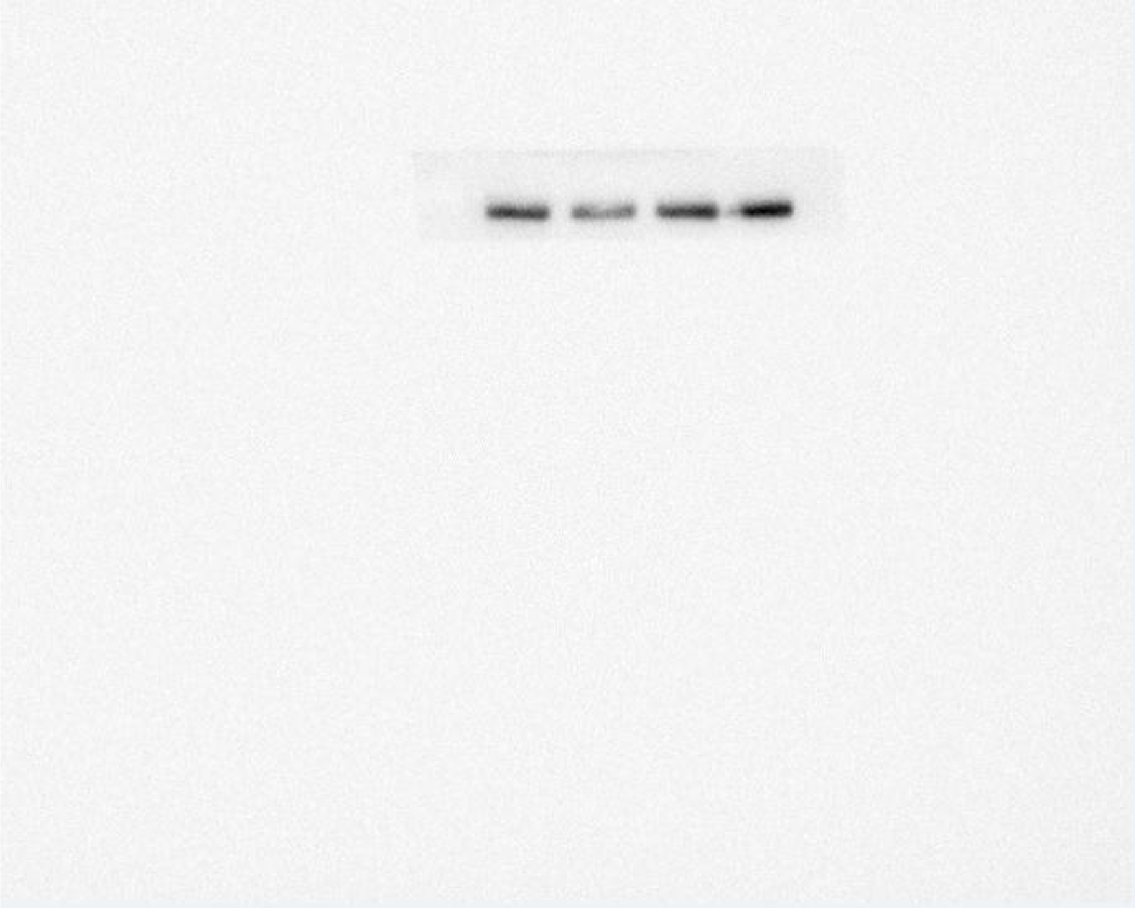

**β-actin**

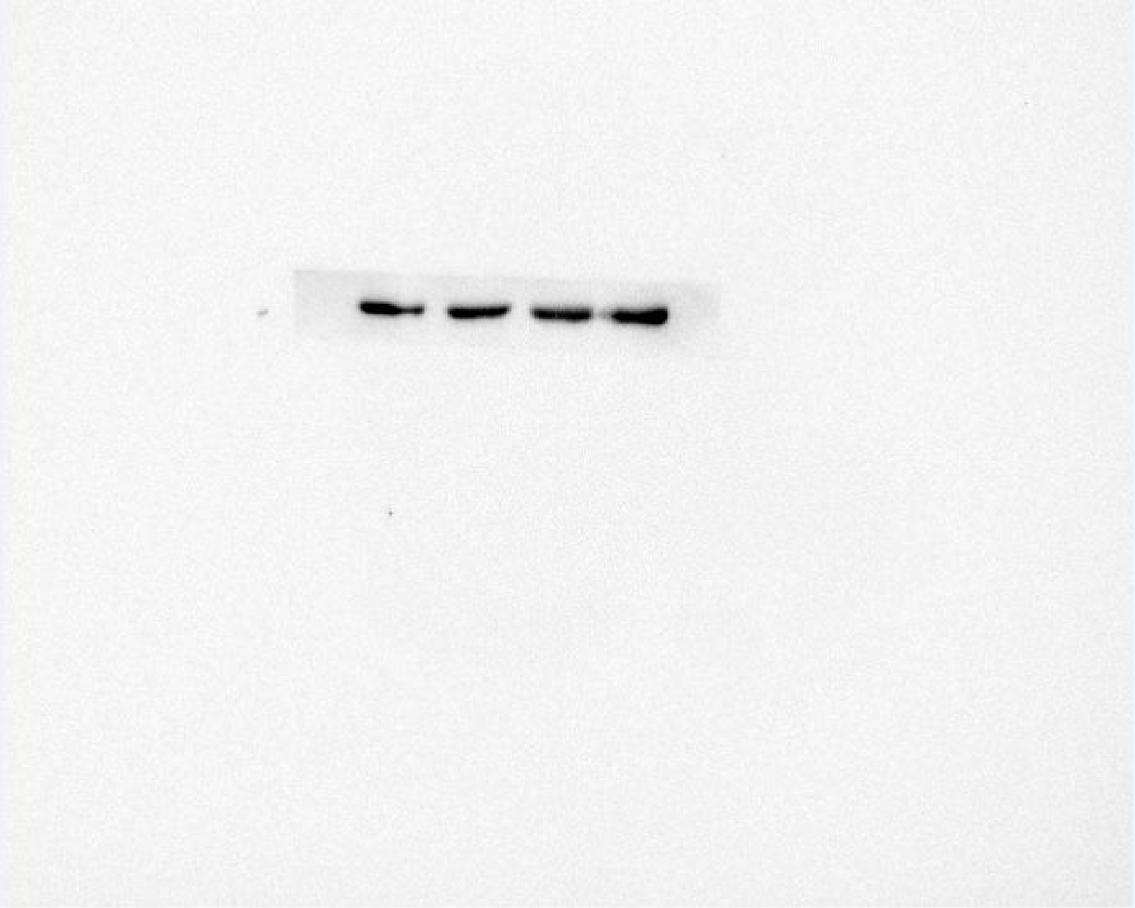

FIG 6A CTS

HMGB1

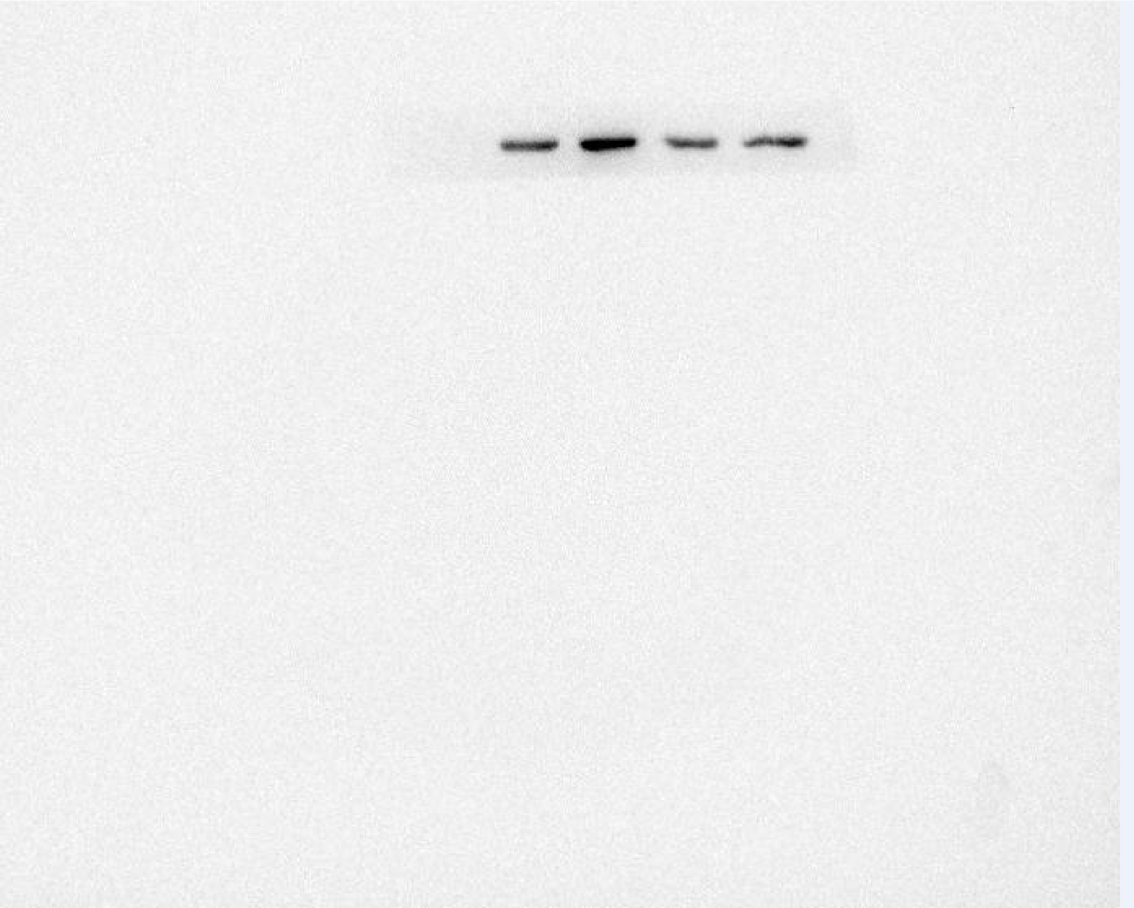

LC3

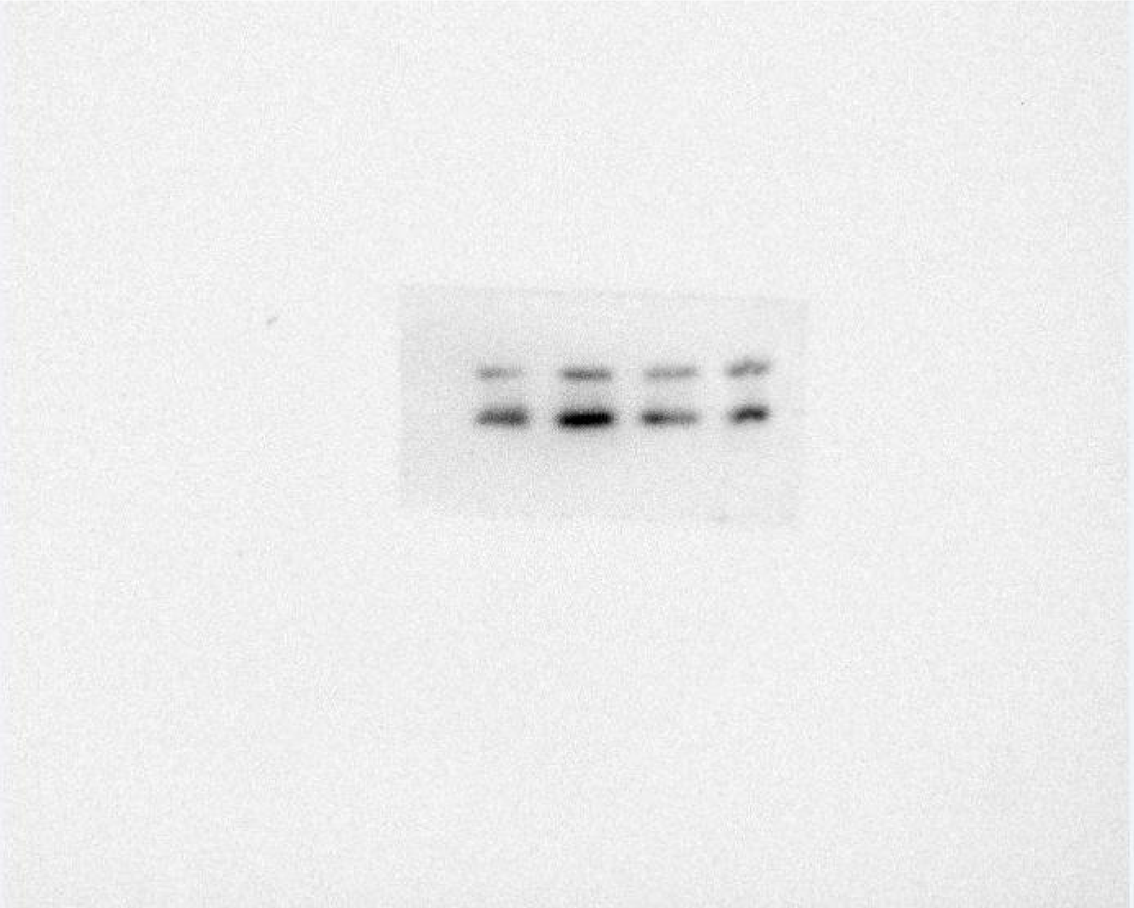

FIG 6B

IGF1R

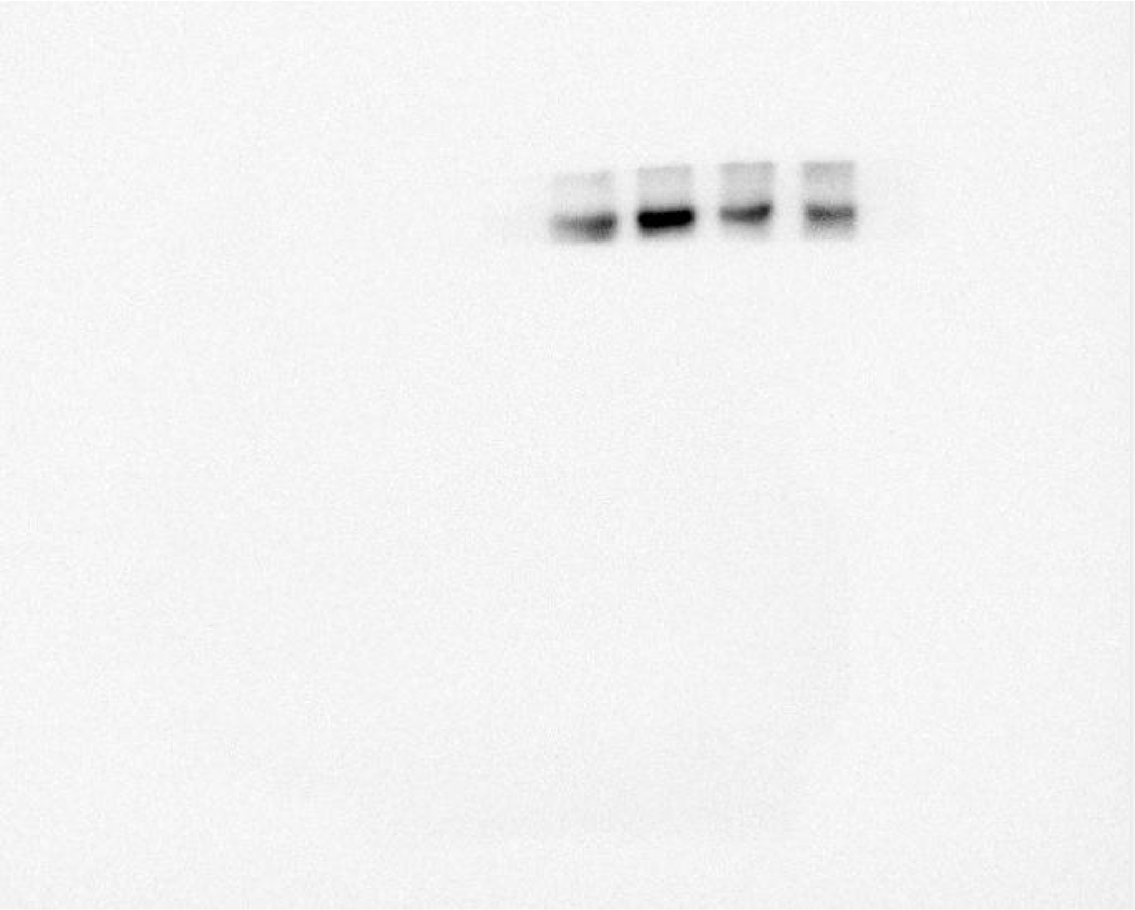

p-STAT3

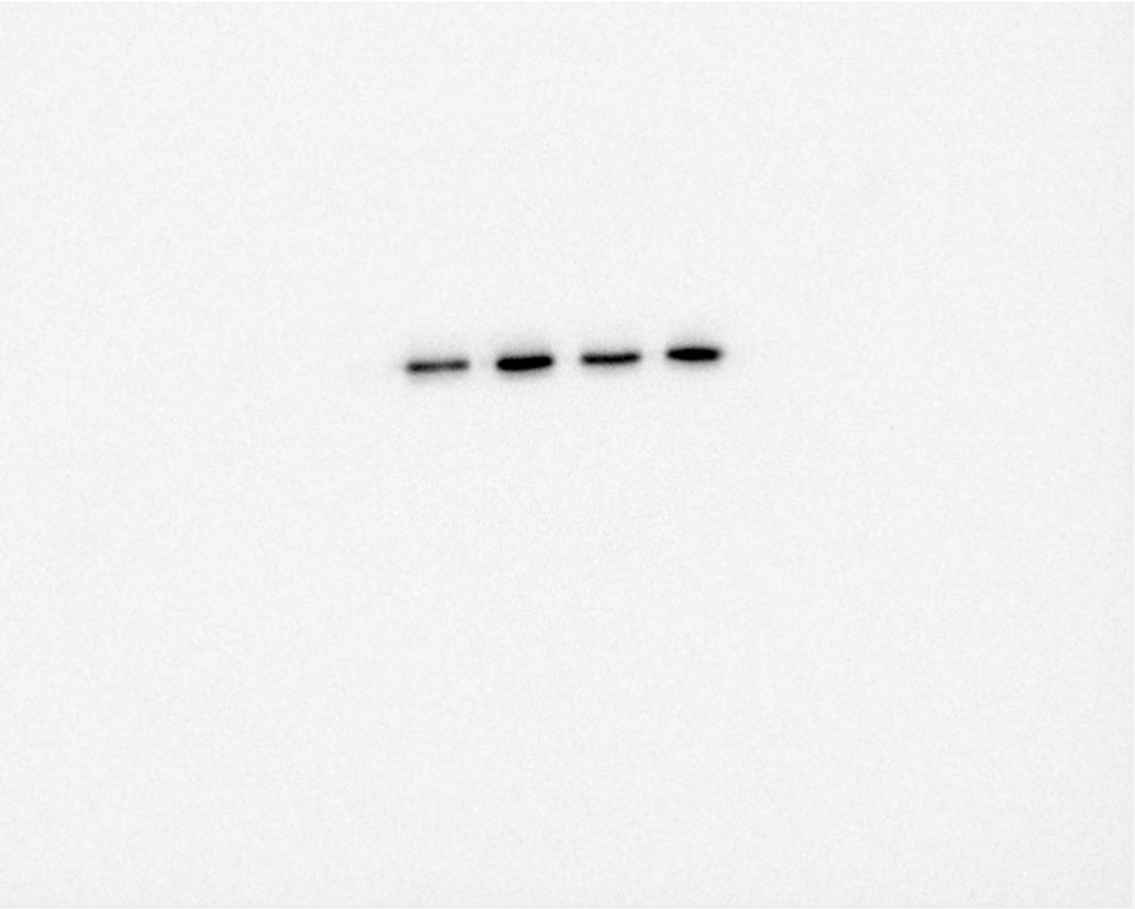

FIG 6B

STAT3

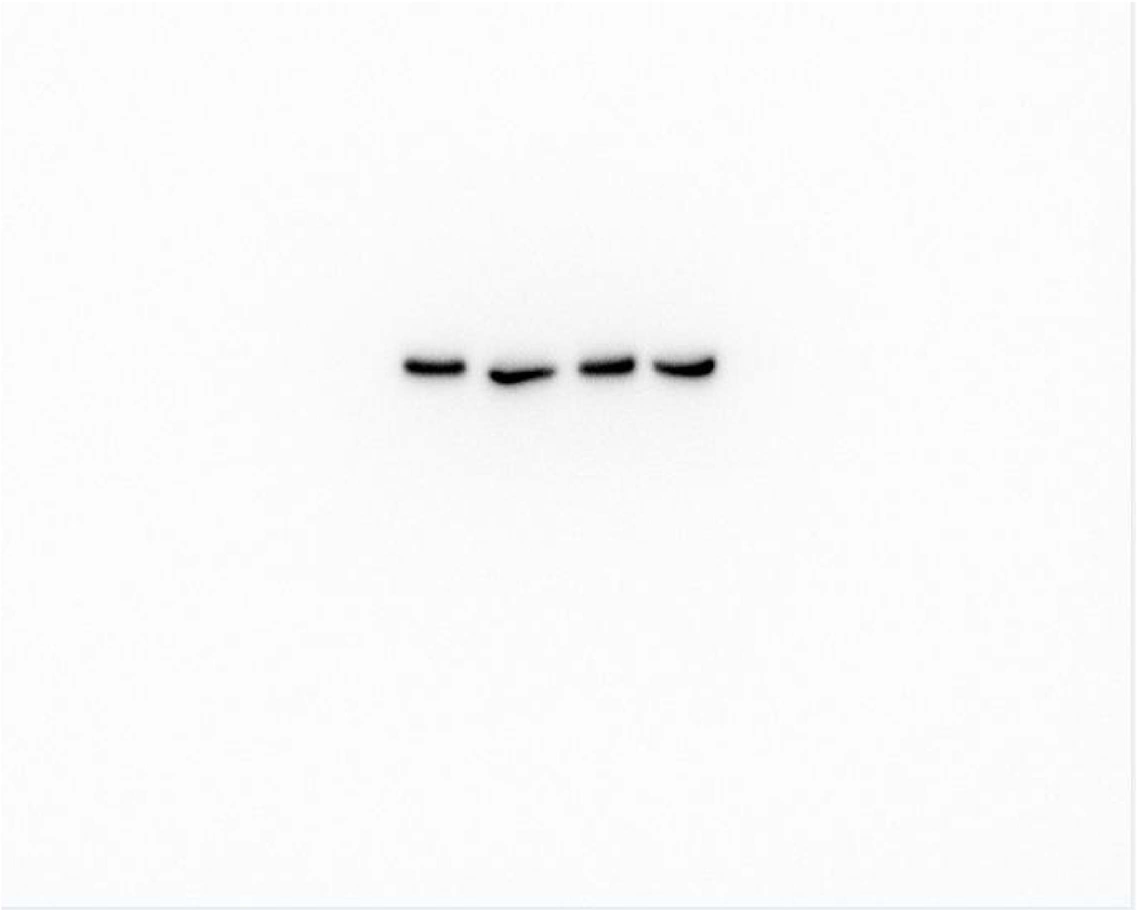

β-actin

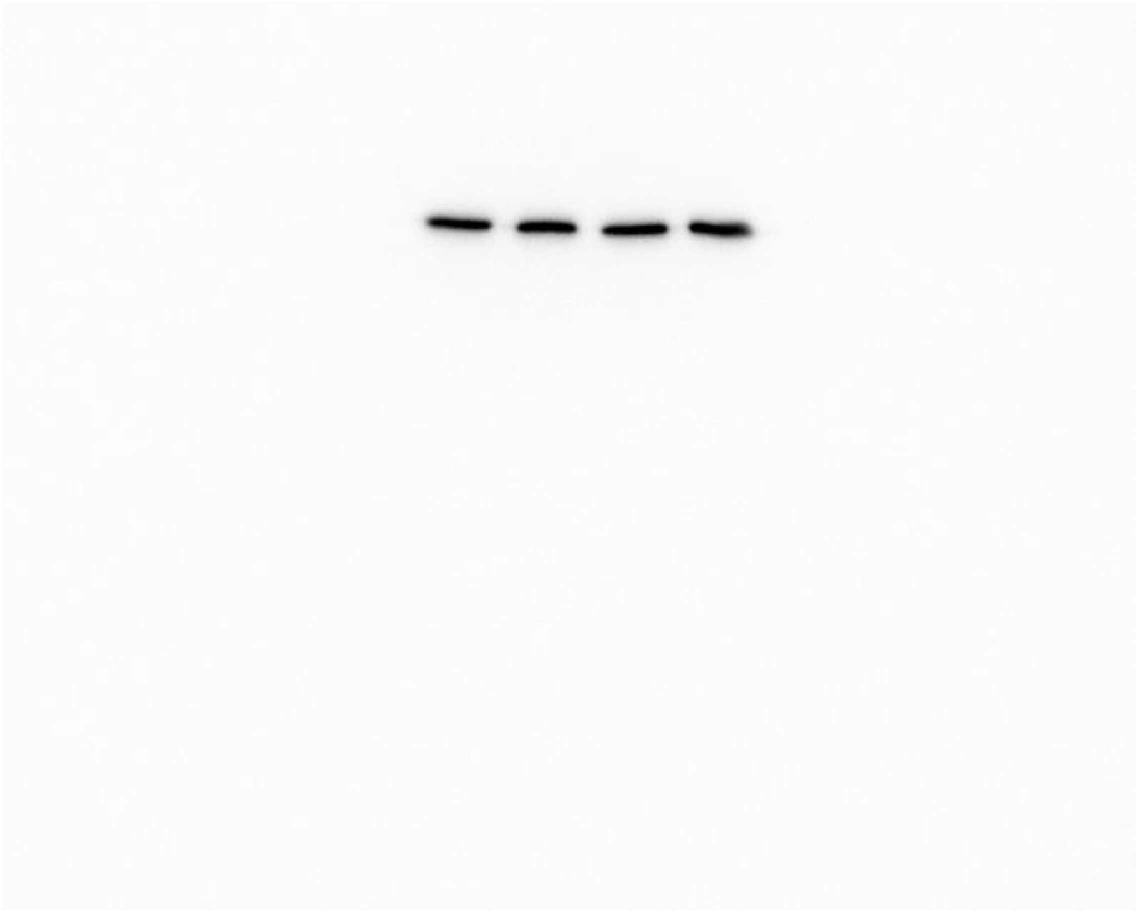

**FIG 6B**

**HMGB1**

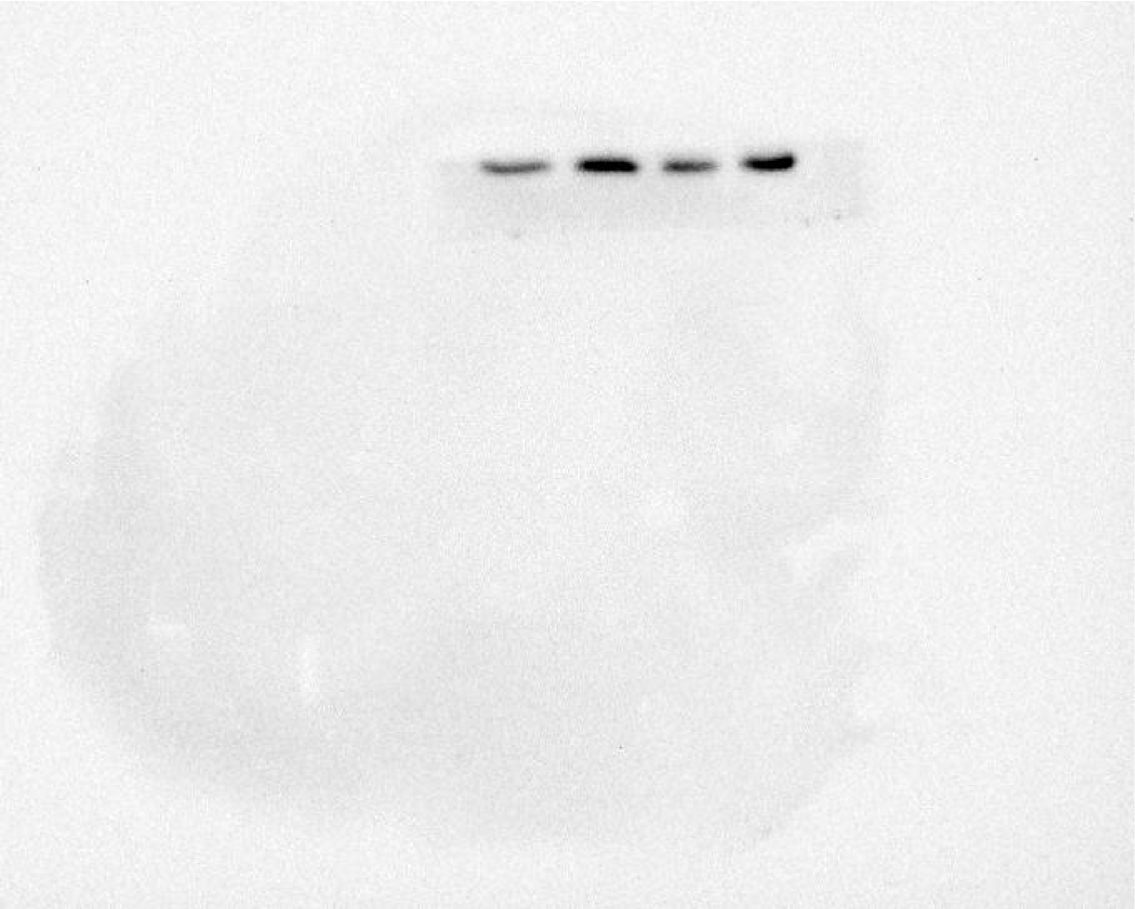

Supplementary Figure 1A

IGF1R

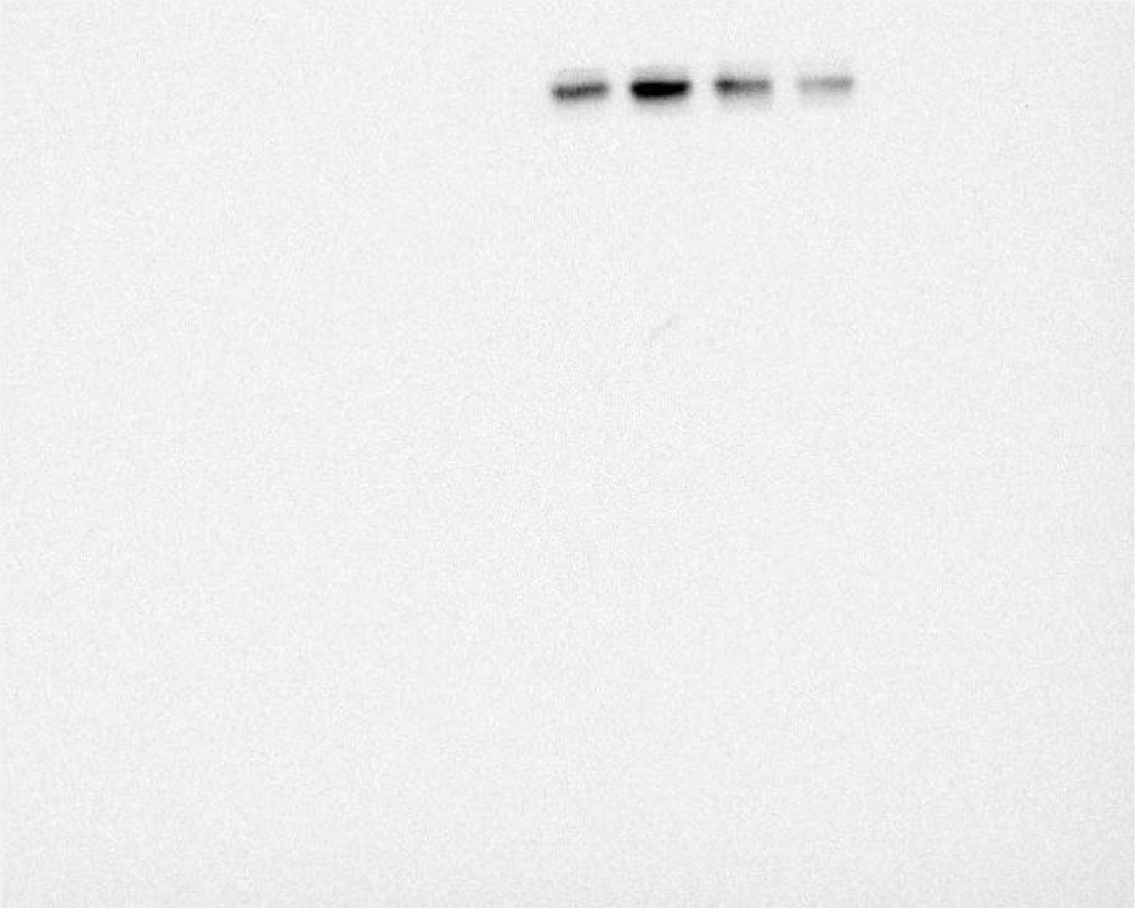

β-actin

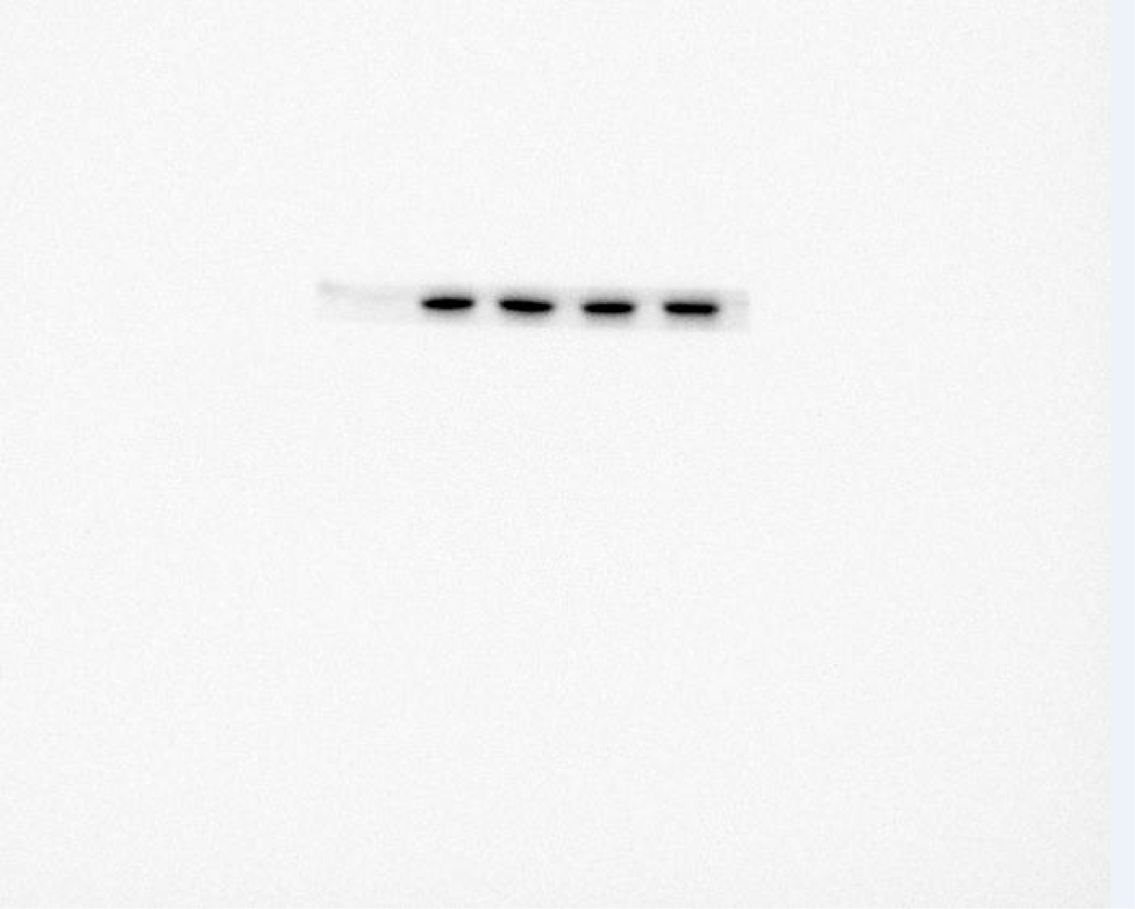

**Supplementary Figure 1A**

CXCL1

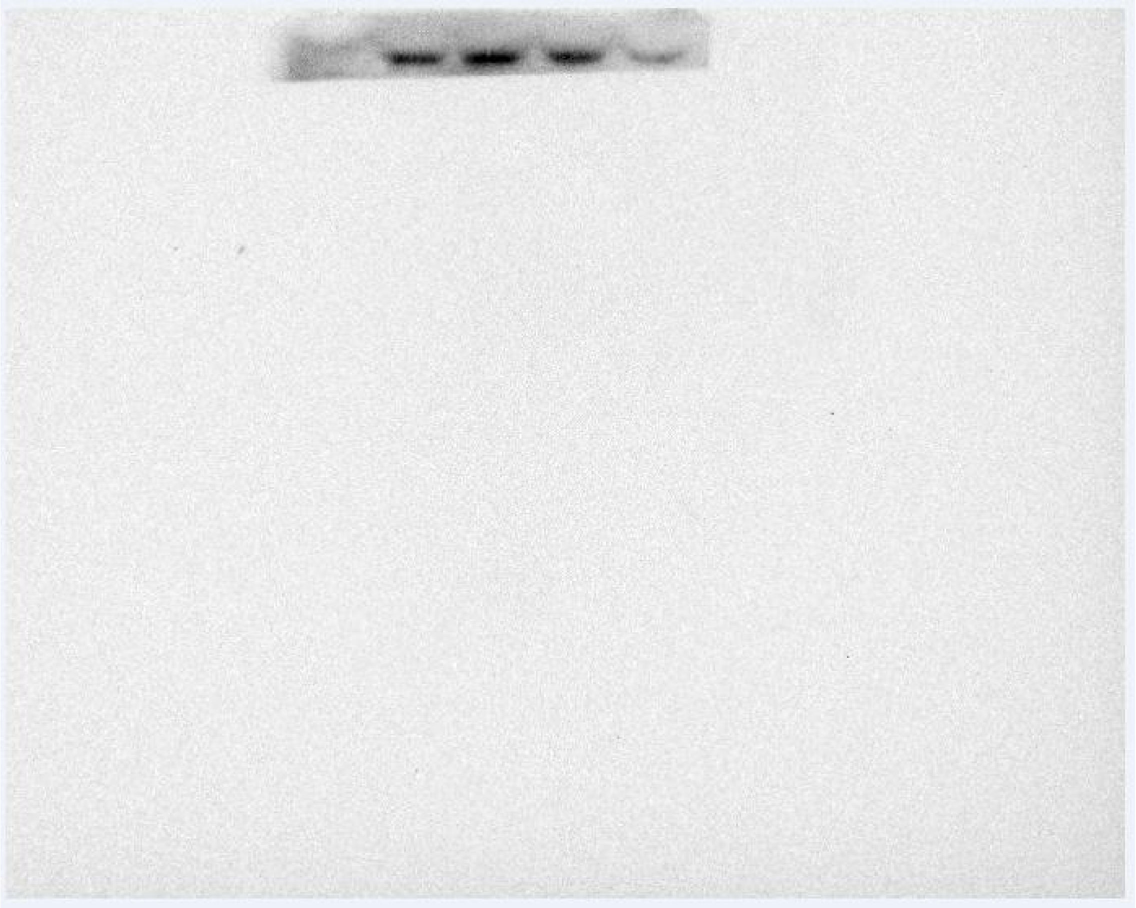

Ubiquitin

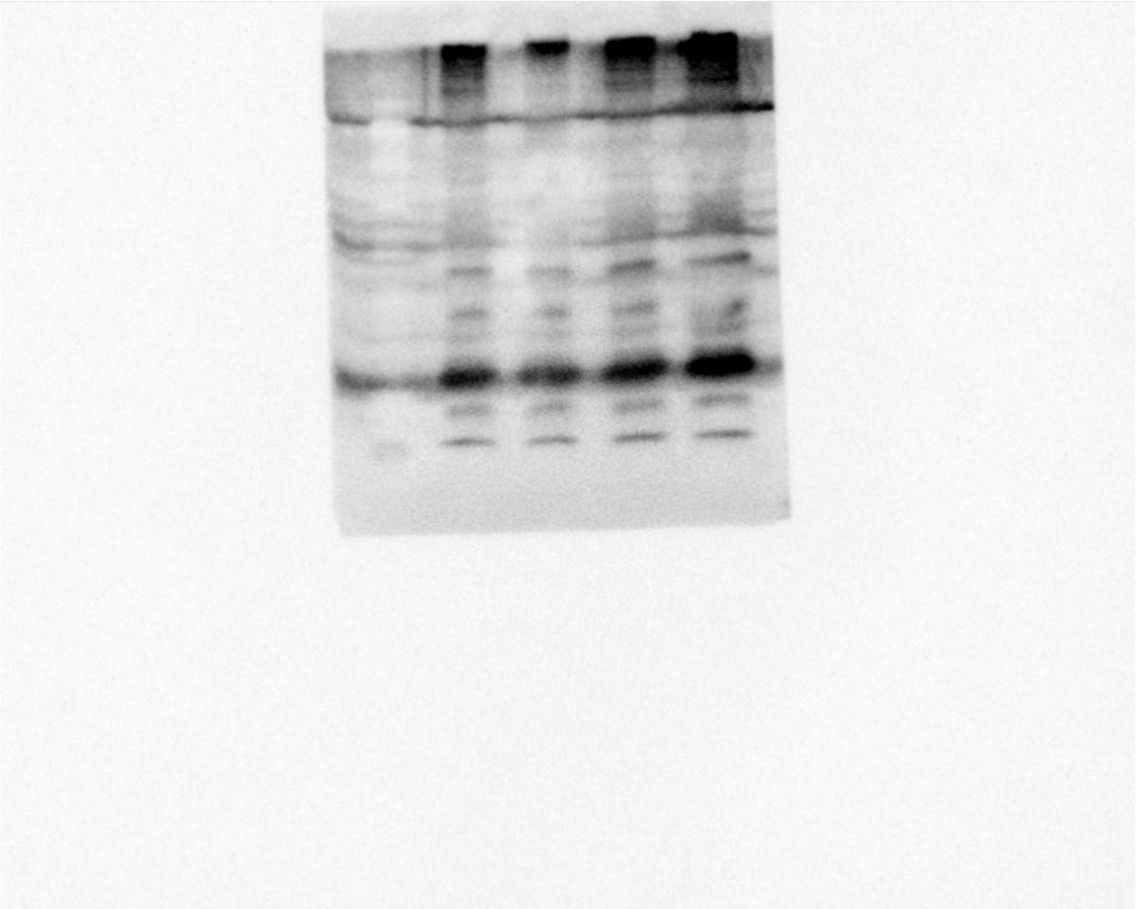

Supplementary Figure 1B *CXCL*$^{OE}$

IGF1R

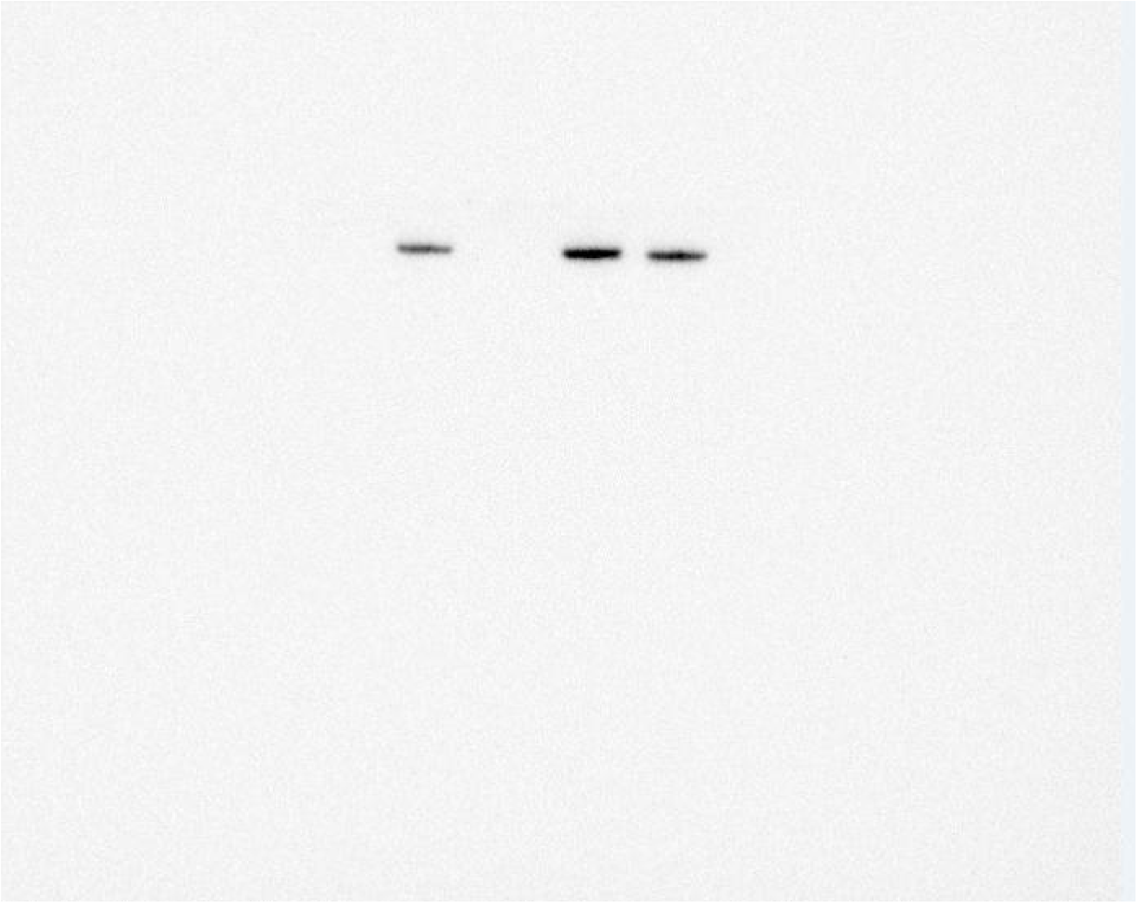

VHL

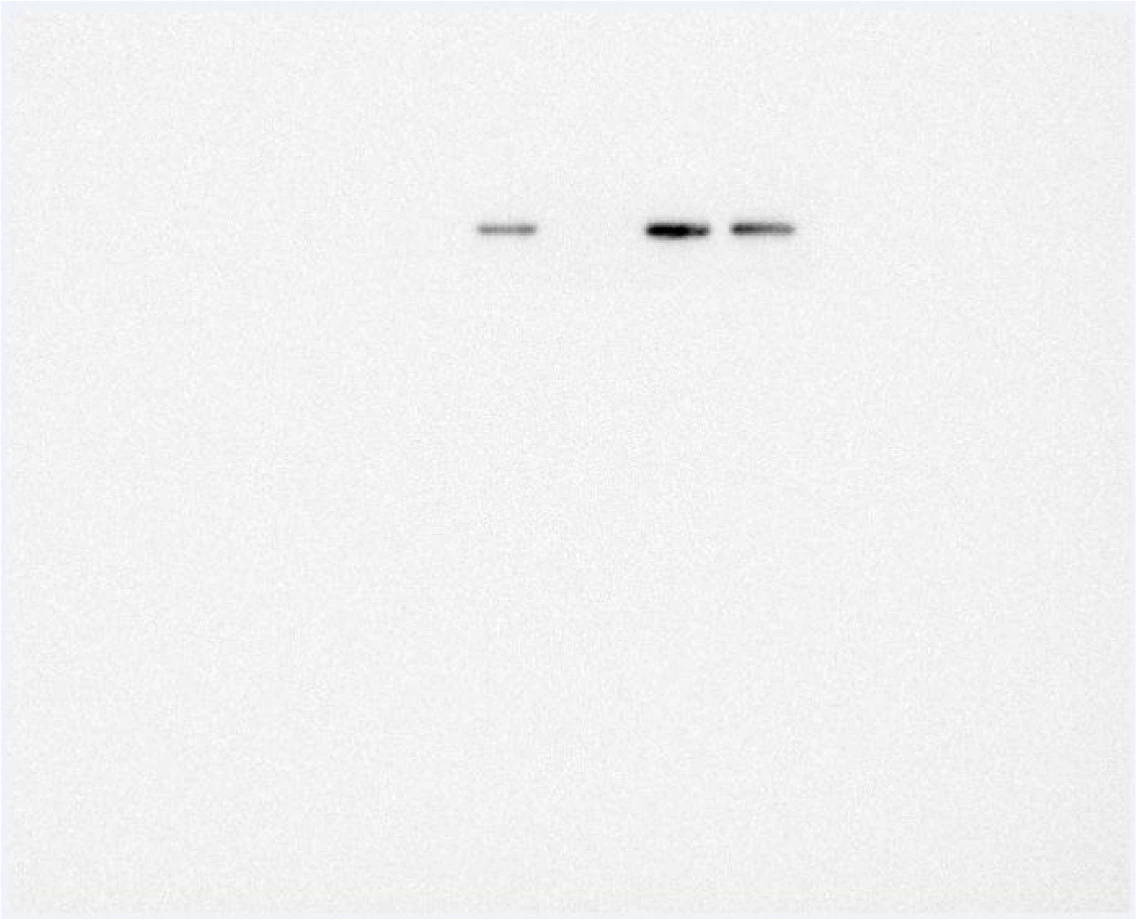

**Supplementary Figure 1B *CXCL*$^{KO}$**

IGF1R

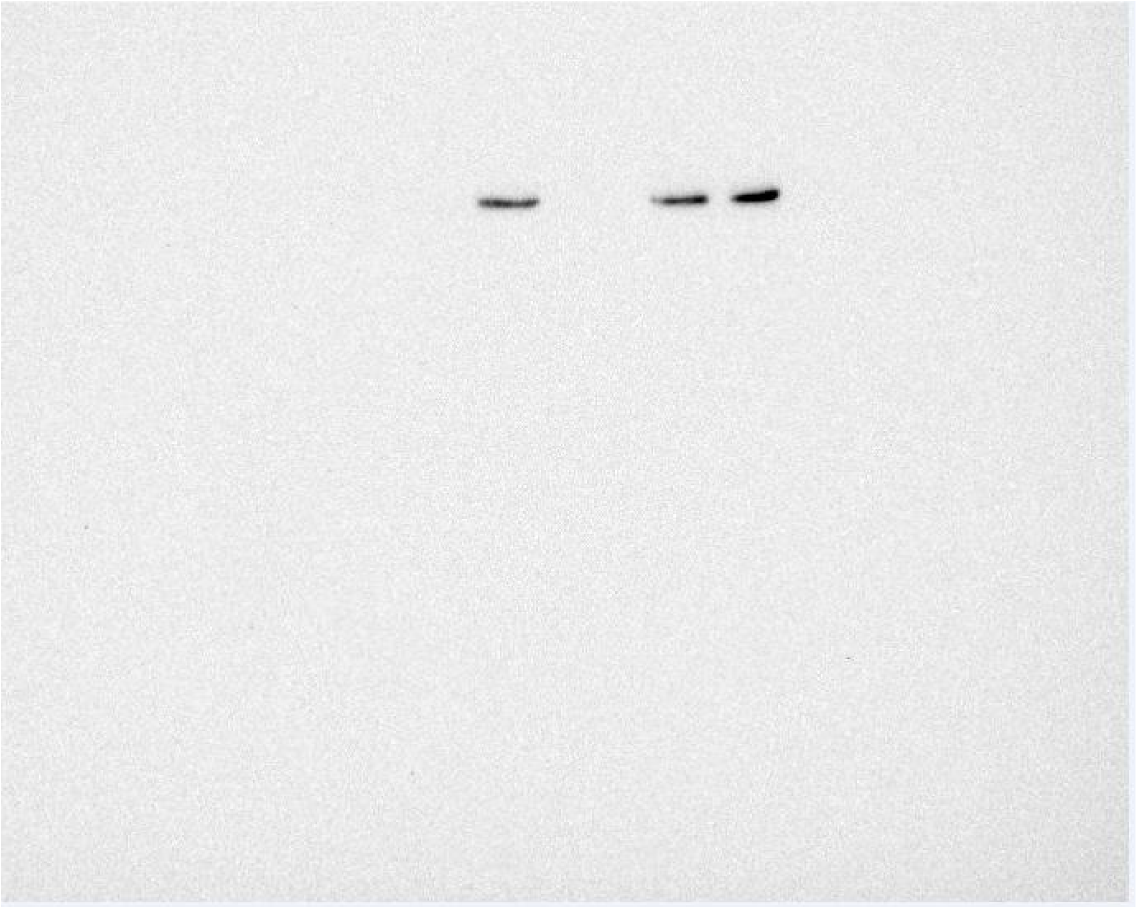

VHL

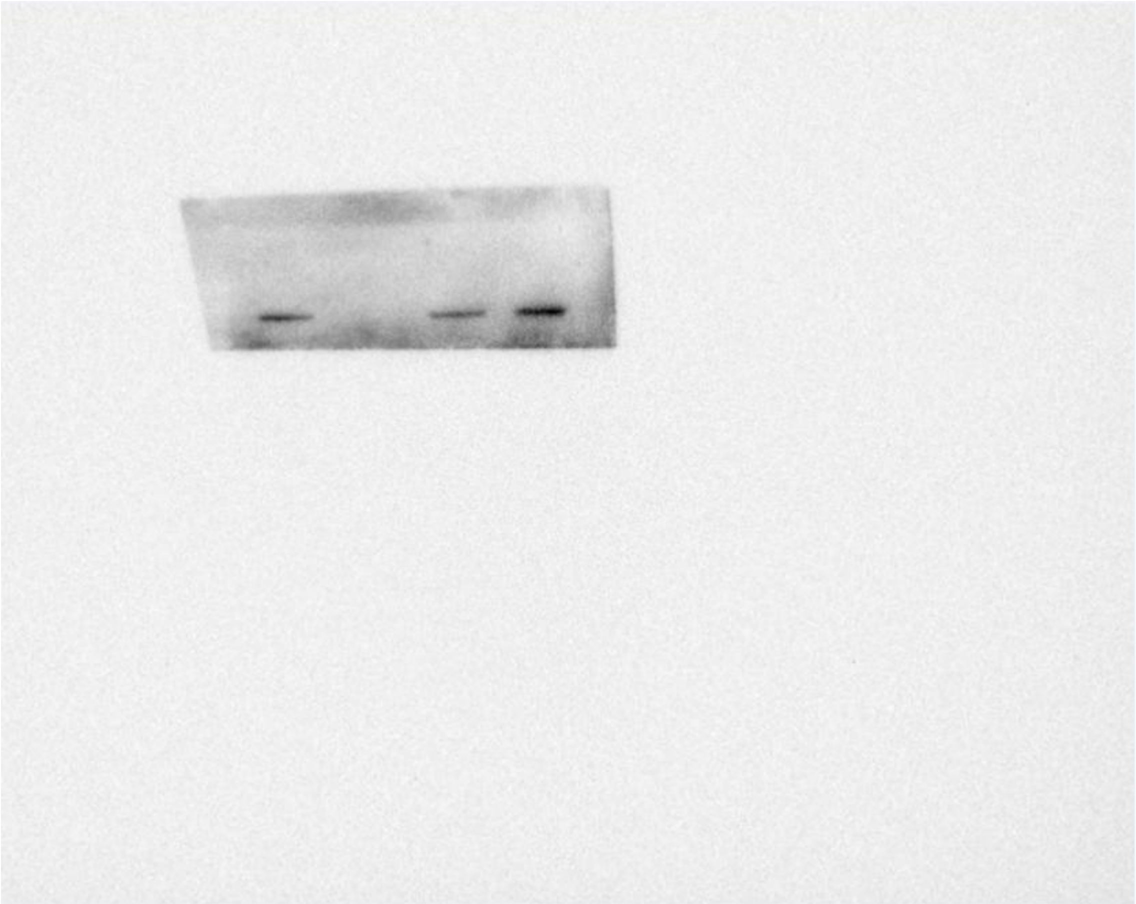

Supplementary Figure 1C

IGF1R

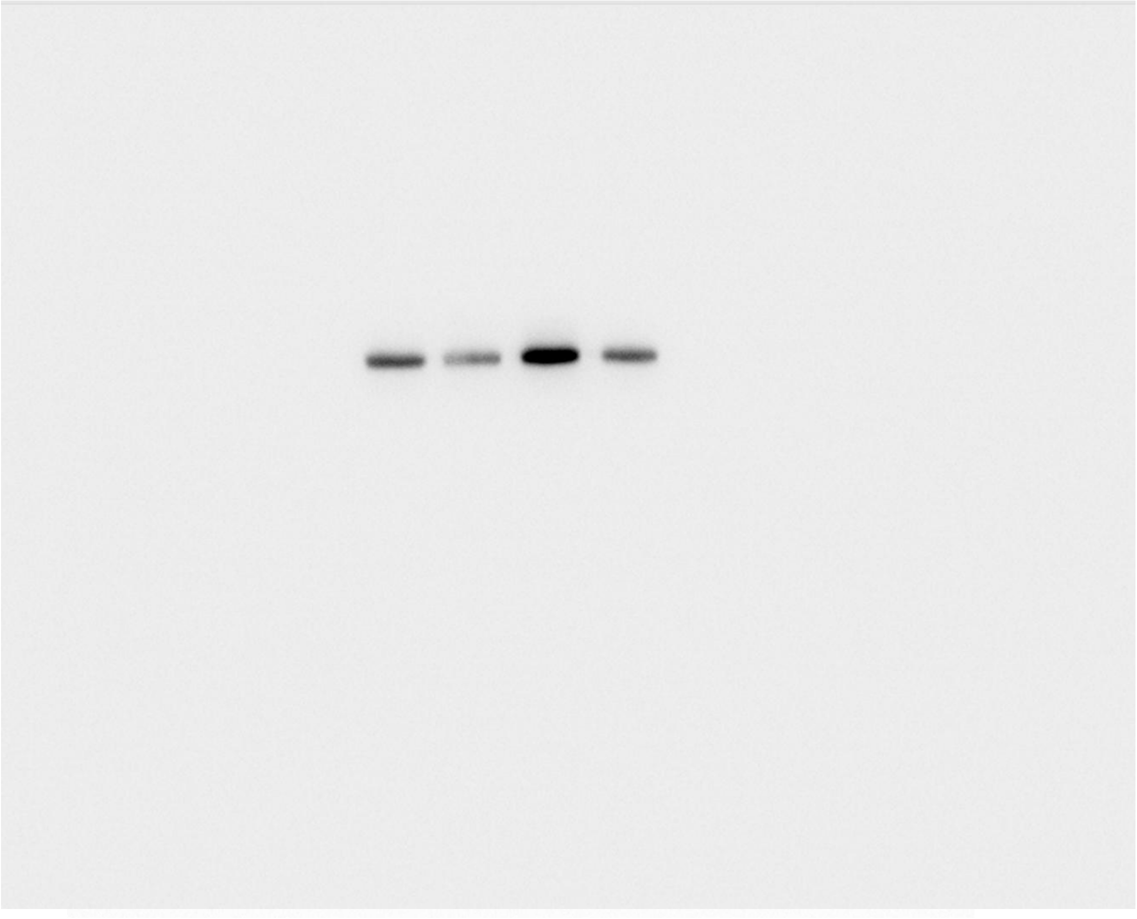

β-actin

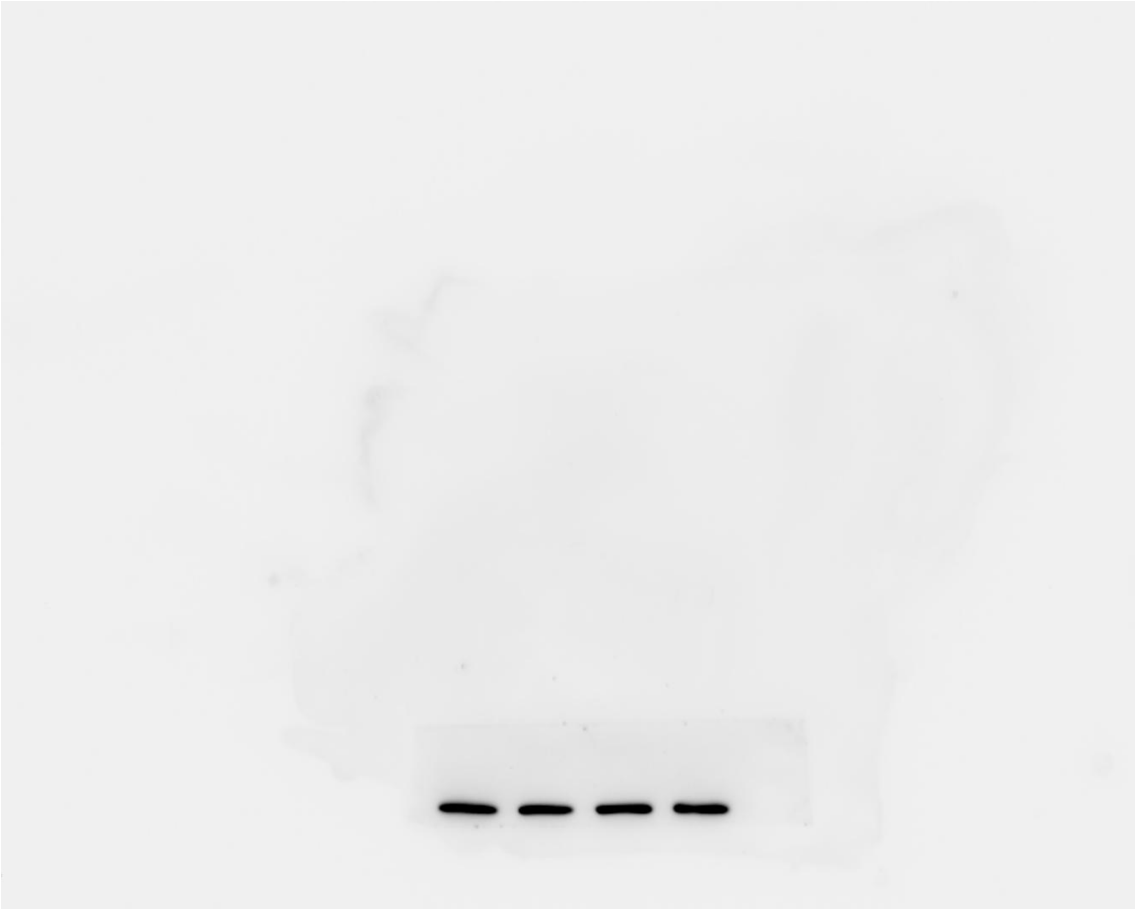

# Supplementary Figure 1C

VHL

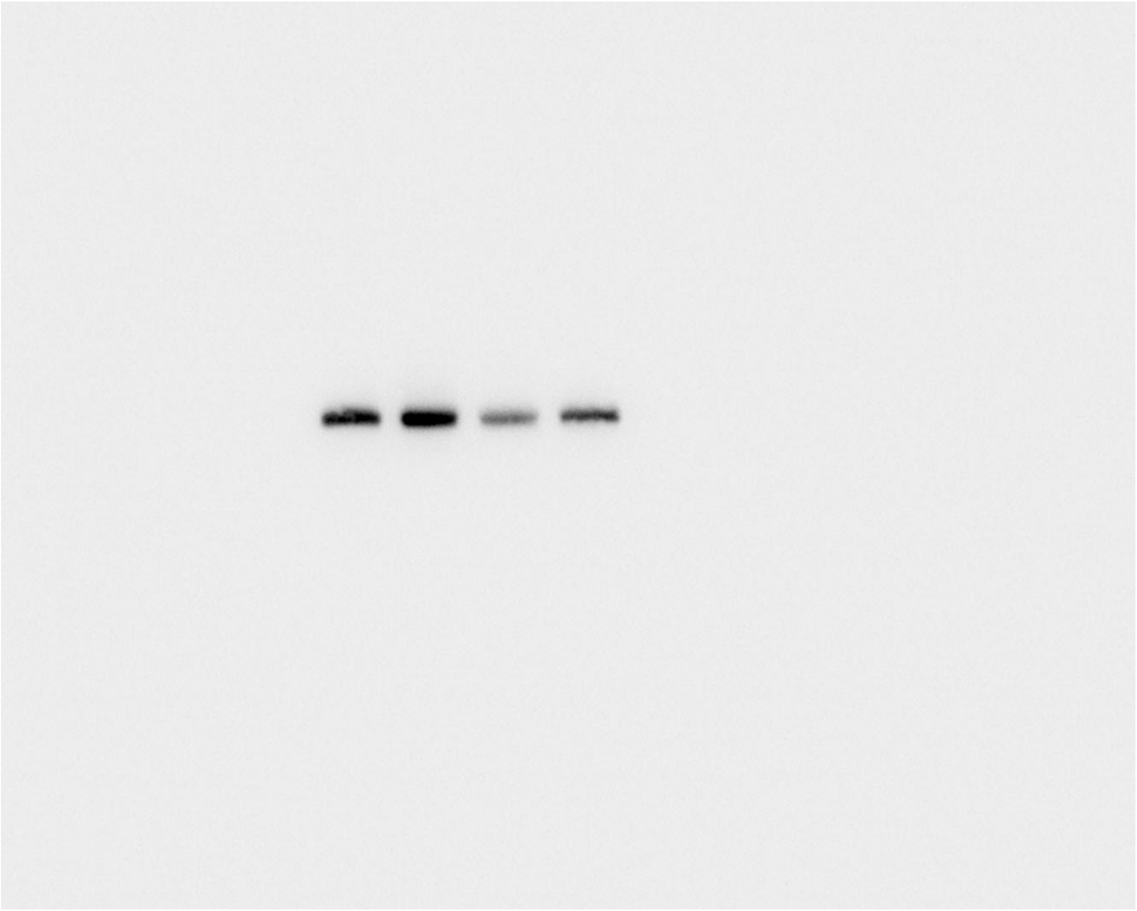

CXCL1

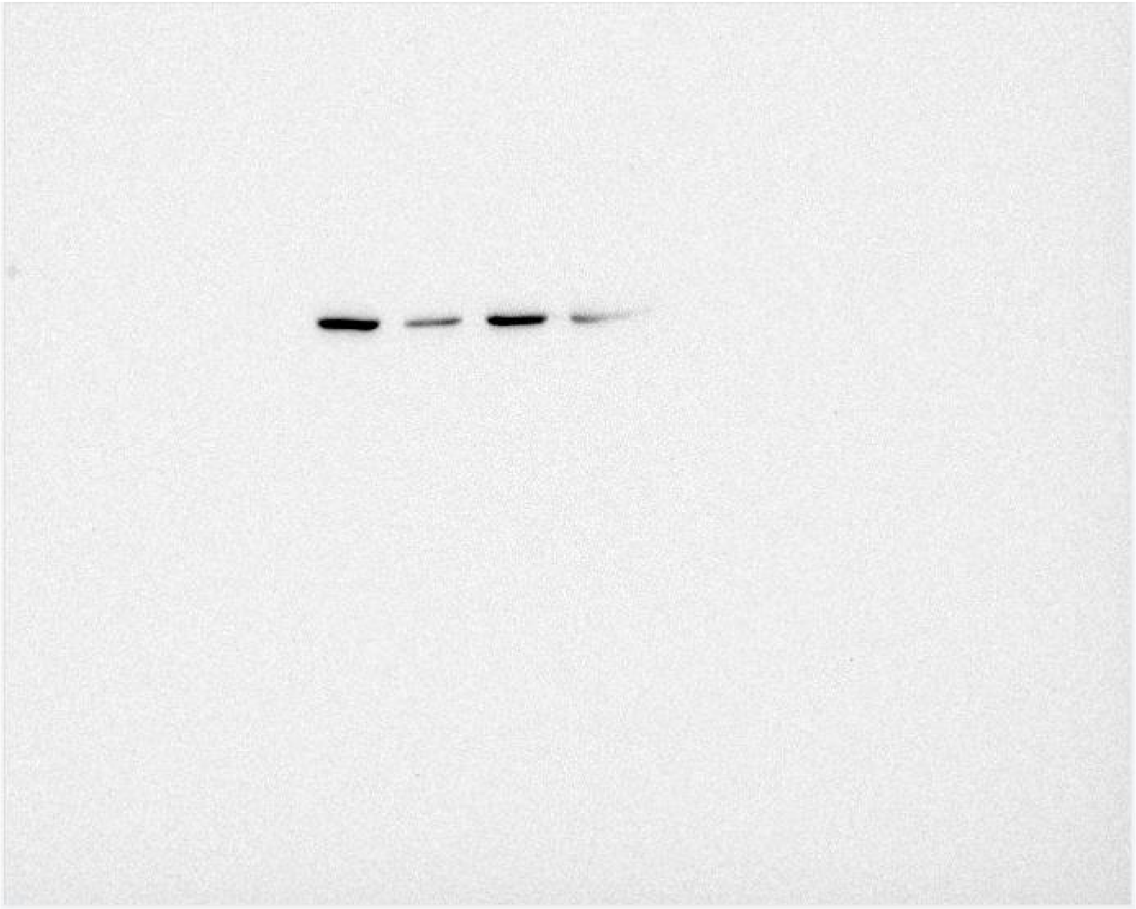

**Supplementary Figure 1C**

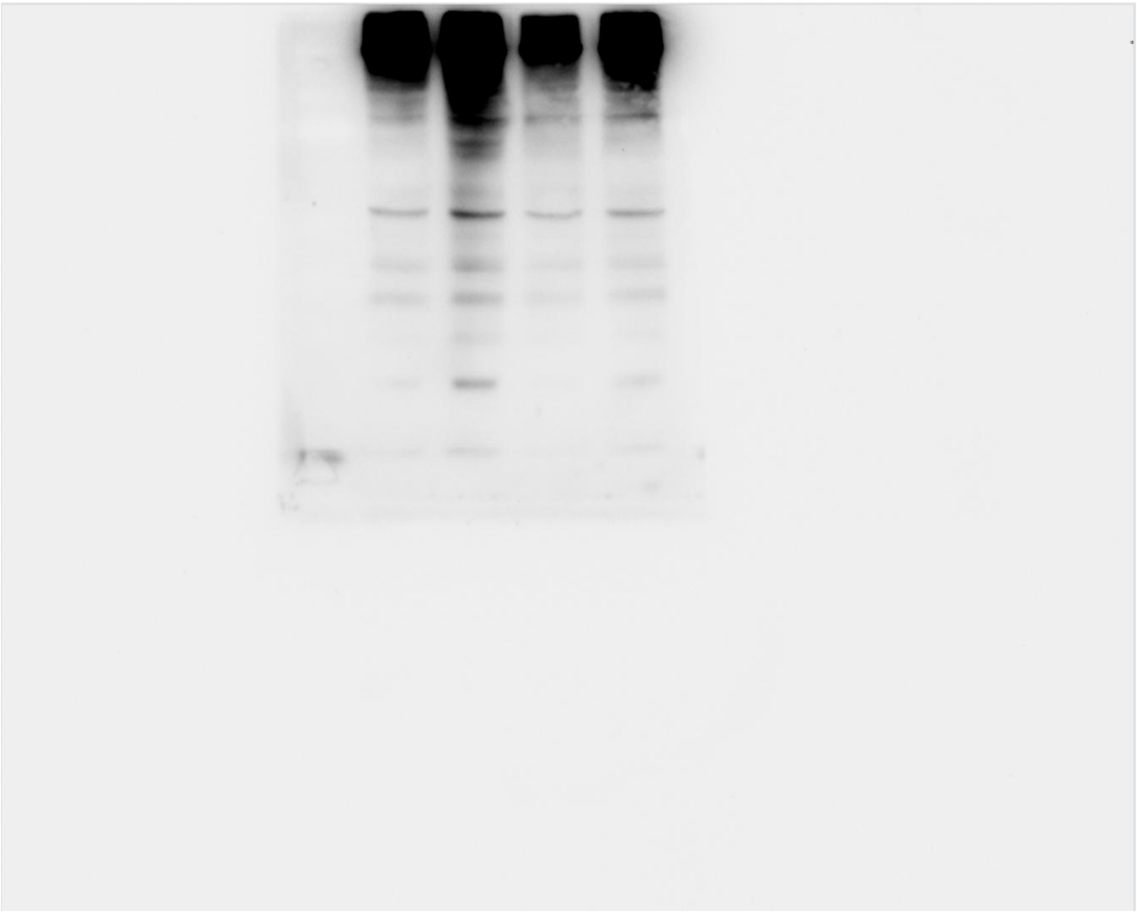

Supplement: Supplementary file 3 — Original images of blot [file 41419_2024_7123_MOESM3_ESM.pdf]
